# A Comprehensive Database for DNA Adductomics

**DOI:** 10.3389/fchem.2022.908572

**Published:** 2022-05-27

**Authors:** Giorgia La Barbera, Katrine Dalmo Nommesen, Catalina Cuparencu, Jan Stanstrup, Lars Ove Dragsted

**Affiliations:** Department of Nutrition Exercise and Sports, University of Copenhagen, Frederiksberg, Denmark

**Keywords:** DNA adduct, database, mass spectrometry, toxicology, carcinogenesis, identification

## Abstract

The exposure of human DNA to genotoxic compounds induces the formation of covalent DNA adducts, which may contribute to the initiation of carcinogenesis. Liquid chromatography (LC) coupled with high-resolution mass spectrometry (HRMS) is a powerful tool for DNA adductomics, a new research field aiming at screening known and unknown DNA adducts in biological samples. The lack of databases and bioinformatics tool in this field limits the applicability of DNA adductomics. Establishing a comprehensive database will make the identification process faster and more efficient and will provide new insight into the occurrence of DNA modification from a wide range of genotoxicants. In this paper, we present a four-step approach used to compile and curate a database for the annotation of DNA adducts in biological samples. The first step included a literature search, selecting only DNA adducts that were unequivocally identified by either comparison with reference standards or with nuclear magnetic resonance (NMR), and tentatively identified by tandem HRMS/MS. The second step consisted in harmonizing structures, molecular formulas, and names, for building a systematic database of 279 DNA adducts. The source, the study design and the technique used for DNA adduct identification were reported. The third step consisted in implementing the database with 303 new potential DNA adducts coming from different combinations of genotoxicants with nucleobases, and reporting monoisotopic masses, chemical formulas, .cdxml files, .mol files, SMILES, InChI, InChIKey and IUPAC nomenclature. In the fourth step, a preliminary spectral library was built by acquiring experimental MS/MS spectra of 15 reference standards, generating *in silico* MS/MS fragments for all the adducts, and reporting both experimental and predicted fragments into interactive web datatables. The database, including 582 entries, is publicly available (https://gitlab.com/nexs-metabolomics/projects/dna_adductomics_database). This database is a powerful tool for the annotation of DNA adducts measured in (HR)MS. The inclusion of metadata indicating the source of DNA adducts, the study design and technique used, allows for prioritization of the DNA adducts of interests and/or to enhance the annotation confidence. DNA adducts identification can be further improved by integrating the present database with the generation of authentic MS/MS spectra, and with user-friendly bioinformatics tools.

## 1 Introduction

Systems toxicology covers the totality of exposures negatively affecting a living organism. It integrates the exposure to toxic chemicals that enter the human body from exogenous sources such as pollutants, food, and drugs, with the exposure to harmful endogenous chemicals derived from cellular metabolism or other processes, including inflammation, oxidative stress, microbial metabolism, and infection. Exposures of many kinds and biological responses of an organism can nowadays be investigated in great detail thanks to the development of new powerful technologies such as metabolomics (Scalbert et al., 2014), proteomics and transcriptomics, (Heijne et al., 2005). The exposure of human DNA to genotoxic compounds induces the formation of covalent DNA adducts which, if not repaired, can lead to gene mutation, possibly initiating the process of carcinogenesis. Given the broadness of the chemical exposome, DNA adducts derive from a large number of compounds, with a wide variety of chemical properties, e.g., polycyclic aromatic hydrocarbons (PAH), nitrosamines and other alkylating agents (Ma et al., 2019), heterocyclic aromatic amines (HAA) (Turesky and Le Marchand, 2011), reactive oxygen species (ROS) (Yu et al., 2016) and many others (Hemeryck and Vanhaecke, 2016). However, many unknown DNA adducts are likely to exist and could help to identify new carcinogenic compounds and their sources. The measurement of DNA adducts is of fundamental importance in assessing the potential carcinogenic effects of toxic compounds from diet and environment and in understanding their mechanisms of action, ultimately leading to the development of new diagnostic, preventive and therapeutic approaches towards cancer. DNA adductomics is a new -omics science that covers the comprehensive measurement of DNA adducts, being a promising technique in systems toxicology (Balbo et al., 2014).

Over the past 30 years, several analytical methods have been developed to measure DNA adducts. While immunochemical methods and ^32^P-postlabeling techniques have been used extensively, liquid chromatography coupled with mass spectrometry (LC-MS) has become the gold standard, as it provides additional information about the chemical identity of the adducts. These analyses are usually targeting one or a few DNA adducts per assay, while failing to provide a global picture of the “DNA-adductome,” i.e., the totality of DNA adducts present in a biological sample. An untargeted approach, on the other hand, would allow the simultaneous profiling of thousands of adducts covering the adductome and in turn enabling the identification of unknown DNA adducts. The relatively recent development of routine high-resolution mass spectrometry (HRMS) allows the identification of unknown adducts with a high confidence level due to high mass accuracy. Therefore, ultra-high performance (UHP)LC-HRMS holds promise to open new horizons in the screening of both known and unknown DNA adducts (Hemeryck et al., 2016; Villalta and Balbo, 2017; Guo and Turesky, 2019).

A considerable bottleneck in the application of HRMS to detect DNA adducts is the assignment of identities to the thousands of features that are detected in a typical untargeted HRMS analysis. Although this has been a challenge also in the application of untargeted metabolomics, open-source MS-based databases and spectral libraries have been a great help for the community to annotate and interpret metabolomics data. Such resources include the Kyoto Encyclopedia of Genes and Genomes (KEGG) (Kanehisa and Goto, 2000), METLIN (Guijas et al., 2018), Human Metabolome Database (HMDB) (Wishart et al., 2022), and MassBank (Horai et al., 2010). However, these databases either do not include DNA adducts or report only a few of them. The application of DNA adductomics is therefore hindered by the lack of available databases, mass spectral libraries and software for identification of DNA adducts; such tools are highly needed for the application of DNA adductomics in future studies. In previous papers, a few databases including either diet-related DNA adducts (Hemeryck et al., 2015), DNA adducts deriving from alkylation, lipid peroxidation products (LPO) and ROS (Carrà et al., 2019a), or bulky hydrophobic DNA adducts (Guo et al., 2017), have been reported. This represents substantial work, but some of these databases are limited to specific classes of genotoxicants, some lack details on the structures and analytical techniques used for identification, and some lack information about the design of the experiment performed. The lack of a comprehensive database including all the different classes of DNA adducts with uniform consensus on names and molecular formulas, limits the application of DNA adductomics, as already highlighted recently (Guo et al., 2020). In this context, the goal of our work is to start, develop and curate a freely available, comprehensive, DNA adduct database, which can be further developed by other contributors with new DNA adducts and completed with generated MS/MS spectra, ultimately resulting in the creation of a proper spectral library for the DNA adductomics community.

With this work, we start off the DNA adduct database by compiling existing databases and existing data retrieved from an extensive literature search. We performed data curation and standardization of the DNA adducts in terms of names, molecular formulas, structures and sources. We reported DNA adducts together with the technique used for identification, the experimental study design and relation to known genotoxicants and sources. We implemented the DNA adduct database with further possible DNA adducts deriving from potential genotoxicants and their combination with all nucleobases. Finally, we included *in-silico* MS/MS fragments for all the adducts and experimental MS/MS fragments for available reference standards. Such a database can be used to compare the measured mass with exact masses of known and suspected DNA adducts and to compare MS fragments with *in silico* generated fragments and experimental MS/MS spectra, representing a valuable tool for supporting the annotation of DNA adducts in untargeted approaches. In addition it provides metadata and information on the plausibility of DNA adducts in terms of source, detection technique and experiment, enhancing the annotation confidence of DNA adducts.

## 2 Materials and Methods

### 2.1 Literature Search

Two preliminary searches were performed in PubMed and Scopus to retrieve: 1) existing DNA adduct databases and 2) reviews of genotoxicant classes that can form DNA adducts. For the first preliminary search a combination of the search terms (Database OR Screening) and (DNA adduct) were used, whereas for the latter the search terms used were (Cancer OR Carcinogenesis) AND (DNA adduct) AND (Review). The two searches were limited to articles published between 2010 and 2020, in English language. A systematic literature search was then performed addressing DNA adducts reported in connection to the following *genotoxicant classes/sources*: mycotoxins, pyrrolizidine alkaloids, aromatic amines (AA), furans, N-nitroso compounds (NOC), PAHs, acrylamide, aldehydes, alcohol, tobacco, pollution, heated foods, meat/red meat/processed meat, and herbs and spices. Individual searches were performed for each genotoxicant class/source among all types of publications, except reviews, in English language. The search terms included: (DNA adduct OR thymine OR thymidine OR cytosine OR deoxycytidine OR guanine OR deoxyguanosine OR adenine OR deoxyadenosine) AND *genotoxicant class/source* AND (Cancer OR Carcinogenesis) AND NOT (Review). The publications were screened for title and abstract, and if they fulfilled the following specific inclusion criteria, they were kept: a) *in vitro* studies, where carcinogens were incubated with either nucleobases, deoxynucleosides, DNA from Calf Thymus and others, or with cells; b) *in vivo* studies, where animals were dosed with carcinogens and DNA from target tissues analysed; c) human studies where both target tissue and surrogates were analysed. Publications including cyclic adducts (Yu et al., 2016), cross-linked adducts (Stornetta et al., 2015; Hu et al., 2019) and phosphate adducts (Ma et al., 2018) were excluded for this first version of the database. A manual secondary search investigating the reference lists of included publications was added. After removal of duplicates, the full text of the articles was read.

### 2.2 DNA Adduct Database Curation and Harmonization

The DNA adducts resulting from the literature search were included in the database if their structure was confirmed with either an authentic reference standard or with nuclear magnetic resonance (NMR). DNA adducts were also included if identified by HRMS coupled with tandem MS/MS, and were labeled in the database as “tentatively identified”. Not all references resulting from the literature search were referenced in the database. Instead, for each DNA adduct, we prioritized publications from c) human studies > b) *in vivo* > a) *in vitro*, and we prioritized MS-based techniques over others. For example, if one adduct was reported in all types of studies, we only referenced two to three papers from b) and c). The names, molecular formulas and structures of the DNA adducts were checked across referenced publications and harmonized. Each DNA adduct was reported with a unique name and abbreviation; for some adducts an alternate name was also provided as commonly used in the literature. A detailed description of rules applied here for the adducts names is given in the Supplementary Material (Supplementary Table S1) The structure of each DNA adduct, was drawn and its corresponding molecular formula and monoisotopic mass calculated using ChemDraw v.19.1.0.8 (Perkin Elmer Informatics, Waltham, MA). All the DNA adducts were reported in their deoxynucleoside form (nucleobase plus deoxyribose -dR), regardless of how they were reported in the original paper. The structures of the DNA adducts in which the modification occurred on the N3 and N7 of dG, the N1, N3, or N7 of dA, and the N3 of dC were shown as positively charged. Positional isomers have been distinguished and considered as separate entries in the database. Diastereoisomers and enantiomers have not been distinguished in order to avoid an excessive number of interchangeable entries. Additional details per DNA adduct covered in the database include: 1) source and causative genotoxicant, 2) experimental conditions or biological sample analysed, 3) technique used for detection and identification, and 4) the corresponding publication. The source and causative genotoxicant were reported based on the corresponding publication and/or other publications, making sure that different literature sources were in agreement, whereas experimental conditions and technique used for identification are specific to the primary publication. One unique entry was associated to each DNA adduct and they were ordered according to their chemical class. The DNA adduct database with metadata (The DNA adduct database in Word format) was uploaded in.docx format in a freely accessible online repository (https://gitlab.com/nexs-metabolomics/projects/dna_adductomics_database).

### 2.3 Implementation of the DNA Adduct Database

A simplified version of the DNA adduct database was converted to an .xlsx file. The file includes information on name, abbreviation, alternative name, source of the DNA adduct, molecular formula and monoisotopic mass of reported DNA adducts. Additional “suspected” DNA adducts deriving from all the possible theoretical combinations of potential genotoxicants with the four nucleobases were added to the database; their structures and molecular formula were drawn and calculated using ChemDraw v.19.1.0.8. The charged monoisotopic masses [M + H]^+^ or [M]^+^ and [M + H-dR]^+^ or [M-dR]^+^ were also calculated for each DNA adduct. The database was then implemented to provide searchable data with computer readable identifiers. In particular, the ChemDraw files (.cdxml) were converted into.mol files and IUPAC nomenclature using ChemScript (PerkinElmer Informatics). The.mol files were subsequently converted to .SDF, SMILES, InChI, InChIKey using Open Babel 3.1.1 (O’Boyle et al., 2011). IUPAC nomenclature, SMILES, InChI, InChIKey were included in the .xlsx file. Hyperlinks to the .cdxml, the .mol files and the reference (for the adducts found in the literature), were included in the .xlsx file. Lastly, the .xlsx, .cdxml, and .mol, have been zipped and uploaded in the repository (The DNA adduct database in Excel format).

### 2.4 Building a Preliminary Spectral Library

#### 2.4.1 Chemicals and Materials

Milli-Q ultra-pure water (Merck Life Sciences, Søborg, Denmark), methanol optima LC/MS grade from Thermo Fisher Scientific (Waltham, MA), and ammonium bicarbonate from Merck (St. Louis, MO.) were used for the UHPLC analysis. The following DNA adducts reference standards, were purchased from Toronto Research Chemicals: 2′-deoxy-N^6^-methyladenosine (N^6^-Me-dA); 5-methyl-2′-deoxycytidine (5-Me-dC); O^6^-methyl-2′-deoxyguanosine (O^6^-Me-dG); N3-methylthymidine (3-Me-dT); N^4^,5-dimethyldeoxycytidine (N^4^,5-DiMe-dC); N^2^-ethyl-2′-deoxyguanosine (N^2^-ethyl-dG); N^6^-(2-hydroxyethyl)-2′-deoxyadenosine (N^6^-(2-OH-ethyl)-dA); 8-oxo-2′deoxyguanosine (8-oxo-dG); etheno-2′-deoxy-β-D-adenosine (1,N^6^-ε-dA); 3,N^4^-etheno-2′-deoxycytidine (3,N^4^-ε-dC); 3-(2-deoxy-β-D-erythro-pentofuranosyl)pyrimido [1,2-a]purin-10(3H)-one (M1-dG); 3-(2-Deoxy-β-D-erythro-pentofuranosyl)-3,5-dihydropyrimido [1,2-a]purine-6,10-dione (6-Oxo-M1-dG); γ-Hydroxy-1,N2-propano-2′-deoxyguanosine (1,N^2^-γ-OH-P-dG) (Acr-1I-dG); N-(2′-deoxyguanosin-8-yl)-4-aminobiphenyl (8-ABP-dG); and N2-(deoxyguanosin-8-yl)-2-amino-3,8-dimethylimidazo [4,5-f] quinoxaline (8-MeIQx-dG). Stock solutions of the DNA adduct standards were dissolved at 1 or 0.5 mg ml^−1^ in methanol, or a mixture of water and methanol. The working solutions were diluted with water to 100 ng ml^−1^.

#### 2.4.2 Acquisition of Tandem Mass Spectrometry Spectra

The analysis of the DNA adduct standards was performed on an H class Acquity UHPLC coupled to a Vion-IMS-qTOF (Waters, Milford, MA) *via* ESI source. The UHPLC system was equipped with a quaternary pump and an autosampler thermostated at 10°C. A C18 HSS T3 column (100 × 2.1 mm, 1.8 μm particle size) (Waters) was used at 0.4 ml min^−1^, at 50°C and with the following gradient: 0–1 min (5% B), 1–21 min (0–99% B), followed by a 2 min wash at 99% B and 2 min equilibration at 5% B where A) was H_2_O with 10 mM NH_4_HCO_3_ and B) MeOH with 10 mM NH_4_HCO_3_. The tuning parameters of the Vion-IMS-qTOF were: capillary voltage 0.5 kV; sampling cone voltage 20 V; source temperature 110°C; desolvation temperature 600°C; desolvation gas 800 (L/h); collision energy 6 eV; cone gas 50 (L/h). The detector voltage was set to 3000 V. The Vion-IMS-Q-TOF was operated in MS/MS acquisition mode with a scan time 0.4 s, in positive polarity. Three MS/MS spectra were acquired at 20, 40, and 60 eV. The mass spectrometer was externally calibrated using the calibration solution Major Mix (Waters). Lock mass correction was applied continuously during the run by injecting 15 μL min^−1^ of 100 ng ml^−1^ leucine/enkephalin (Waters) every 5 s. Raw data files were acquired by UNIFI software (version 1.9.4.053) (Waters) and they were exported as .xlsx files.

#### 2.4.3 Creation of a Searchable Online DNA Adduct Database

**FIGURE 1:**
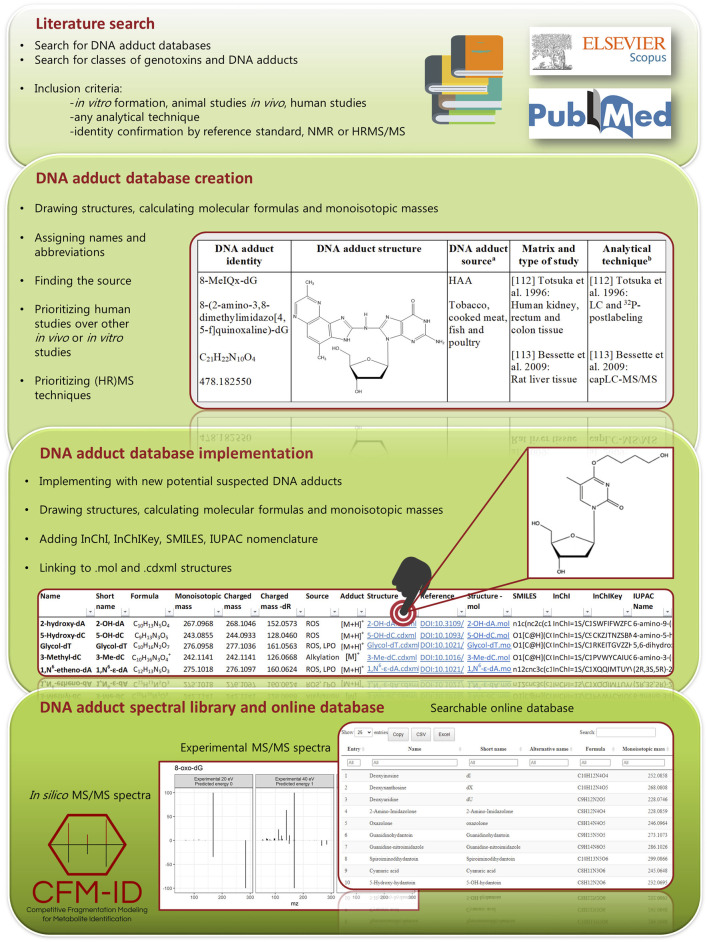
Overview of the steps included in the creation of the DNA adduct database.

The information contained in the .zip file (point 2.3) was converted into a user friendly web-based searchable database to facilitate an easy and straight forward search within all the categories (columns) of the database, i.e., name, short name, alternative name, source, monoisotopic masses, SMILES, InChI, InChIKey,.mol file and .cdxml file. For this, R Project for Statistical Computing software (R Core Team, 2020) version 4.1.1 together with the R package DT (https://github.com/rstudio/DT) and a number of Tidyverse R packages (Wickham et al., 2019) were used to create the interactive and searchable datatable (https://datatables.net) that included all of the above mentioned information [The DNA adduct database (online)]. *In silico* fragments were generated for all the adducts at three energy levels by using the docker image (https://hub.docker.com/r/wishartlab/cfmid) of CFM-ID (Wang et al., 2021), and the fragments above 5% intensity were included in a datatable [The database of predicted fragments (online)]. Finally, a separate datatable with all experimental fragments [The database of experimental fragments (online)] was created. A single entry was created for each fragment ion for improving searchability. An overview of the steps included in the creation of this database is provided in Figure 1.

## 3 Results

### 3.1 Literature Search

The preliminary literature search aimed at retrieving existing DNA adduct databases resulted in 390 hits in Pubmed and 377 in Scopus, of which three articles were selected after reading through title, abstract and text. The three publications (Hemeryck et al., 2015; Guo et al., 2017; Carrà et al., 2019a) were used to build a preliminary DNA adduct database. The second preliminary search, conducted to reveal genotoxicant classes that can form DNA adducts, resulted in 88 review articles in Pubmed and 423 in Scopus. After reading through title and abstract, many genotoxicant classes and sources were found, that were classified in 1) eight larger genotoxicant classes: mycotoxins, pyrrolizidine alkaloids, AA (incl. HAAs), furans (incl. cis-butene-dial, furfuryl alcohol and hydroxymethyl-furfural), NOC (incl. nitrosamines and N-nitroso-pyrrolidine), PAHs, acrylamide, aldehydes (incl. several α,β-unsaturated aldehydes, formaldehyde, acetaldehyde, malondialdehyde, crotonaldehyde, glyoxal, acrolein) and 2) and six larger sources: alcohol, tobacco, pollution (i.e., environmental, industrial and chemical contaminants), heated foods (i.e., charred or grilled meats, cereals and others), meat/red meat/processed meat, and herbs and spices. Additional adducts listed in database arose from secondary searches and include adducts deriving from additional eight genotoxicant classes (incl. ROS, LPO, reactive nitrogen species (RNS), estrogens, alkylating agents, halogenation products, aristolochic acid and alkenylbenzenes) and three sources or processes (incl. endogenous processes, UV irradiation, plant-based foods). An overview of the individual literature searches is given in Supplementary Table S2. In total, 279 DNA adducts have been included in the database. A total of 132 publications have been cited in the database following our decision to include only up to three papers per adduct, prioritizing human studies over *in vivo* over *in vitro* studies.

### 3.2 The DNA Adduct Database

**FIGURE 2:**
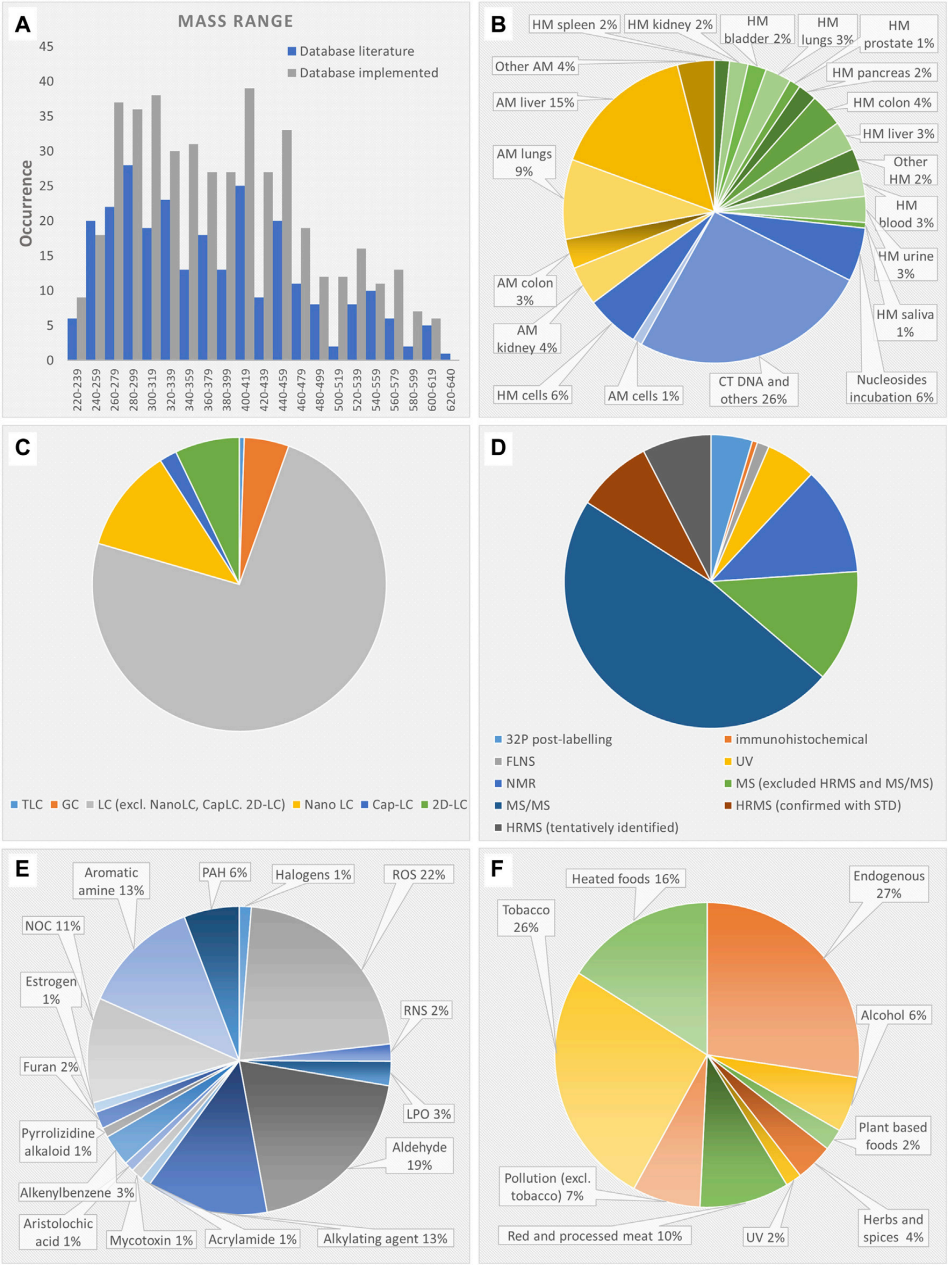
**(A)** mass range distribution of the DNA adduct database before and after implementation with suspected DNA adducts; **(B)** samples where the DNA adducts have been analyzed; **(C)** chromatographic technique used for DNA adduct analysis; **(D)** technique used for DNA adduct identification; **(E)** DNA adduct causative genotoxicants grouped in 16 classes where “aromatic amines” includes heterocyclic aromatic amines, “LPO” excludes aldehydes, i.e., α,β unsaturated aldehydes, malondialdehyde, formaldehyde, glyoxal, acrolein, crotonaldehyde, “NOC” includes N-nitroso pyrrolidine and nitrosamine; **(F)** sources of the causative genotoxicants grouped in nine classes. Abbreviations: LC, liquid chromatography; MS, mass spectrometry; capLC, capillary LC; FLNS, fluorescence spectrometry ; GC, gas chromatography; HRMS, high resolution MS; MS/MS, tandem mass spectrometry; NMR, nuclear magnetic resonance; TLC, thin layer chromatography; HM, human; AM, animal; LPO, lipid peroxidation product; NOC, N-nitroso compounds; PAH, polycyclic aromatic hydrocarbons; RNS, reactive nitrogen species; ROS, reactive oxygen species.

The names of the DNA adducts were found to be heterogeneous across different publications. In addition, many DNA adducts derive from multiple genotoxicants and/or exposures. Therefore, after removal of duplicates and standardization of identities and structures, the final DNA adduct database includes 279 structures. The DNA adduct database with metadata is available in.docx format both in the repository (The DNA adduct database in Word format) and in the Supplementary Material (Supplementary Table S3). The mass range of the DNA adducts reported in the database is between 220 and 640 Da, and the distribution did not change after implementation with suspected DNA adducts, as shown in Figure 2A. Although priority was given to publications deriving from c) human studies > b) *in vivo* animal studies > a) *in vitro* studies, the studies were equally distributed among *in vitro* (38%), *in vivo* (35%) and human studies (27%) as shown in Figure 2B. The majority of *in vitro* studies were performed on DNA from calf thymus and few others (26%). The animal tissues that were studied the most were liver (18%), lung (12%) and colorectal (7%) tissue. The majority of the DNA adducts in humans were found in target tissues (77%), i.e., related to the tissue where cancer develops, rather than surrogate samples (23%) (i.e., blood, urine, saliva). As shown in Figure 2C, most of the adducts were analyzed by using LC (94%) rather than GC or TLC. The advanced 2D-LC, nano-LC, and capillary LC account for 20% of the chromatographic techniques. As shown in Figure 2D, 14% of DNA adducts were identified by UV, fluorescence spectroscopy or immunoanalysis. The ^32^P-postlabeling method was used for 5% of DNA adducts. 12% of DNA adducts were identified with NMR. The majority of DNA adducts (78%) were identified by MS. Only 16% were identified by HRMS coupled to tandem MS/MS. However, half of them were only tentatively identified since they were not confirmed with reference standards. As shown in Figure 2E, the genotoxicants responsible of adduct formation were grouped in 16 different classes. The majority of DNA adducts derived from ROS (22%), alkylation (13%), and aldehydes (19%). As shown in Figure 2F, the different sources of DNA adducts were grouped in nine large classes, however some overlap. The majority of DNA adducts derived from endogenous sources (24%), tobacco (24%) followed by heated, cooked and grilled food (15%).

### 3.3 Implementation of the DNA Adduct Database

The DNA adduct database has been posted on the online repository (The DNA adduct database in Excel format) as a downloadable .xlsx file in order to create a practical MS tool for DNA adduct identification by us and others. The updated database includes 582 entries, 279 of which have been previously found in the literature and 303 are suspected DNA adducts. It is possible to distinguish the two classes of adducts by noticing that the suspected ones are not linked to any reference. Furthermore, the structure of the suspected DNA adducts describe only a plausible position, and the position of the modifications on the nucleobase has not been specified in the name. The number of dT adducts among the suspected adducts is considerably smaller than those derived from dG, dA and dC, since many DNA adducts cannot be formed by conjugation or modification of dT (e.g., etheno-dT cannot be formed because of the lack of an amino group).

### 3.4 Preliminary Spectral Library and Searchable DNA Adduct Database

The DNA adduct database including the precursor ions [M + H]^+^ or [M]^+^ and the fragment ions deriving from -dR loss has been transformed into an interactive online database, for allowing search of monoisotopic masses [The DNA adduct database (online)]. In addition the spectra of 15 DNA adduct reference standards were acquired at three different collision energies and were used for retrieving the most significant fragments of the DNA adducts and uploading them in a searchable web datatable [The database of experimental fragments (online)]. The experimental MS/MS spectra and the *in silico* fragmentation spectra obtained with CFM-ID were compared and reported in the Supplementary Material (Supplementary Figures S1–S15). The comparison showed considerable overlap, indicating that the predicted fragments can be used for supporting DNA adduct annotation. Therefore, the predicted fragments were also reported in a separate datatable [The database of predicted fragments (online)].

## 4 Discussion

In this paper we introduce a newly curated and freely available database for DNA adductomics starting from an extensive literature search, and aiming to ultimately create a proper spectral library for the DNA adductomics community.

### 4.1 Data Curation and Harmonization

Following the results of the literature search, there was a clear necessity for data curation and harmonization of DNA adducts structures, names and sources as these were often found in disagreement across different publications.

The structures of DNA adducts have been mainly reported in their nucleobase form in the older papers, where acidic hydrolysis of DNA and other approaches led to the breakage of the bond between -dR and the nucleobase. However, since most recent approaches use enzymatic hydrolysis of DNA, the DNA adducts are reported here in their deoxyribonucleoside form (DNA adduct with -dR).

Different structures were proposed in the literature for adducts where the modification of the nucleobase occurred on an N sitting on a double bond of the heterocycle (e.g., N3 and N7 of dG; N1, N3, or N7 of dA; the N3 of dC). Some DNA adducts have been drawn with a positive charge (Tang et al., 2019), some in the zwitterionic form by adding a negative charge in the structure, and some in their neutral form by saturating the double bond (Essigmann et al., 1979; Li et al., 1992). Since both the zwitterionic and saturated forms are chemically unlikely, we have reported all such DNA adducts in their most likely form with a positive charge. This has to be taken into account when calculating and analyzing the charged adducts [M + H]^+^ and [M]^+^ by MS.

As shown in Supplementary Table S4, where a comparison of three previously reported DNA adduct databases (Hemeryck et al., 2015; Guo et al., 2017; Carrà et al., 2019a) has been carried out, different names were used for the same DNA adduct. In this work, a set of systematic rules was proposed for harmonizing names of DNA adducts as reported in Supplementary Table S1. However, when it was not practical to apply these rules, the name commonly accepted in the scientific community was kept together with an alternative name (e.g., Cro-1I-dG = 1,N^2^-(Me-OH-P)-dG, Cro-2II-dG = N^2^-Paraldol-dG). The complexity in finding an agreement between different names led us to also report the IUPAC nomenclature.

Besides names and structures, inconsistency was also found regarding the sources of DNA adducts (see Supplementary Table S4). In this database, the source was associated to DNA adducts based on literature reviews (Bakhiya and Appel, 2010; Turesky and Le Marchand, 2011; Wei and Yin, 2015; Hemeryck and Vanhaecke, 2016; Lee and Ryu, 2016; Stegelmeier et al., 2016; Yu et al., 2016; Alshannaq and Yu, 2017; Ma et al., 2019; Zhang et al., 2021; Eisenreich et al., 2021), or based on the specific publication where the adduct was identified. Both the causative genotoxin and its source have been reported (The DNA adduct database in Word format). Multiple sources were reported for some DNA adducts, since they may derive from different biological processes, foods or environmental factors. Several DNA adducts deriving from endogenous metabolic processes such as alkylation, oxidation and LPO may also be related to intake of different foods, or to various environmental exposures. Furthermore, several exogenous compounds can derive from different sources, such as PAHs and HAAs from smoking, pollution or charcoal grilling of meat (Ma et al., 2019). Due to the complexity and multiplicity of DNA adduct sources, the .xlsx database (The DNA adduct database in Excel format) was simplified by reporting only the causative genotoxin. As a result, the DNA adduct database was harmonized in terms of structures, names and sources, finding consensus among the literature references, thereby establishing a new starting point for future DNA adductomics studies and for a community effort to complete the database.

### 4.2 Plausibility of DNA Adduct Identification

The chemical and biological plausibility of the DNA adducts reported in the literature are major determinants for addition into the current database. Some entries in the previous databases and in the literature include 1) a lack of structural characterization of the DNA adduct, 2) no specification of the technique used for detection, or 3) no specification of the biological sample where the DNA adduct was formed; all of these limitations may compromise the reliability of DNA adduct identifications. MS has gradually become the technique of choice in DNA adductomics, because of its sensitivity and selectivity, which facilitates the elucidation of the chemical structure, especially when MS/MS and/or HRMS are employed (Villalta and Balbo, 2017). In this database, the majority of DNA adducts was identified by MS-based techniques, including low resolution MS, MS/MS and HRMS. However, DNA adducts identified by NMR, and DNA adducts identified by fluorescence, UV, immunoassays, and ^32^P-postlabelling, after comparison with reference standards, were included as unequivocally identified. The only included DNA adducts that are not confirmed with reference standards are those identified by HRMS/MS, since the accurate measure of both the precursor and fragment ions increases the reliability of identification. However, these DNA adducts have been marked as “tentatively identified”. Additional DNA adducts tentatively identified by low resolution MS/MS can be eventually added in the future, if detailed fragmentation patterns are provided (Chang et al., 2021).

The biological plausibility of the identified DNA adducts depends on the chosen experimental approach. The DNA adducts included in our database derive from: a) *in vitro* studies, b) *in vivo* animal studies, c) human studies, prioritizing c) > b) > a) as rating the relevance of the adduct for humans (i.e., quality of the evidence). The detection of DNA adducts *in vitro* does not necessarily reflect the *in vivo* DNA adductome, and not all DNA adducts found in animals are observable and pro-carcinogenic in humans (Cohen and Arnold, 2011). Therefore, we rated their biological plausibility as c) > b) > a) and it can be used as an extra degree of screening for DNA adduct identification. However, a) and b) can still be relevant for monitoring potential new DNA adducts in humans. Due to the difficulty in obtaining unlimited amounts of target tissues from humans, 73% of DNA adducts were found *in vitro* and in animals, rather than in humans. The DNA adducts reported in humans were mostly found in target tissues, however 22% of DNA adducts were found in surrogate samples, such as blood and urine. DNA adducts found in urine belong to the class of DNA adduct-repair products and they are both excreted in their deoxynucleoside (with -dR) form and nucleobase form (without -dR) (Cooke et al., 2018; Chao et al., 2021). However, in this database they were all reported in the form of their original DNA adduct (with -dR) for consistency. Future updates of the database should consider differentiation of DNA adducts and their related DNA adduct-repair products. In conclusion, the chemical and biological information provided in the database should be evaluated for the reliability and plausibility of the DNA adducts reported in the literature.

### 4.3 An MS Tool for Identification

The current DNA adduct database was created not only to give a comprehensive overview of existing DNA adducts, but to offer a tool for targeted and untargeted LC-MS analysis, screening and annotation of DNA adducts. The database was implemented with new potential DNA adducts, providing additional molecular formulas and masses to screen for identifying unknown DNA adducts. The.xlsx file containing the 582 DNA adducts can be uploaded to software for MS data handling and annotation such as MZmine (Pluskal et al., 2010) or XCMS (Smith et al., 2006), and can be also used as inclusion list in data dependent acquisition (DDA) approaches (Carrà et al., 2019a). Both ions and formulas have been included to enable the user to overcome different requirements in the software packages in terms of entry formats. In addition, this is the first database that provides specific structural information summarized in InChI, InChI key, SMILES and with a direct link to .chemdraw and.mol files. This information can be uploaded into any software for annotation and *in silico* fragmentation. The database has been made available in a data repository (https://gitlab.com/nexs-metabolomics/projects/dna_adductomics_database) under Creative Commons as CC-BY 4.0, and it represents the first publicly available MS tool for DNA adduct annotation.

### 4.4 Tandem Mass Spectrometry Spectra and *in Silico* Prediction

The number of known and possible DNA adducts is large. As a consequence, the annotation process can produce a large number of false positives. DNA adduct identification requires comparison of multiple orthogonal properties, including accurate mass and retention time in a well defined LC system, preferably matching with authentic reference standards. This process is costly and often impossible due to the lack of DNA adduct standards. However, including MS/MS mass spectra is one of the main approaches for improving the likelihood of correct feature annotation. In this database, we included the MS/MS spectra acquired from a total of 15 DNA adducts, with the purpose of building the first DNA adduct spectral library. However, due to the limited number of experimental MS/MS spectra available, *in silico* generation of MS/MS spectra is the second best option. For this, we chose to use CFM-ID as we provide here some evidence for a good fit with the included MS/MS spectra of 15 DNA adduct standards. However, due to a certain degree of uncertainty in all predictions, the approach proposed by Carra’ et al., could be used in the future for generating more accurate *in silico* spectra (Carrà et al., 2019b). The incorporation of fragment ions produced with CFM-ID, or other approaches, can be used as a screening tool for selecting the features that are most likely DNA adducts. However, if/when DNA adduct reference standards become available, we encourage all researchers working in the field to upload them in the current DNA adduct database, by following the contact information in the GitLab repository (https://gitlab.com/nexs-metabolomics/projects/dna_adductomics_database).

### 4.5 The New DNA Adduct Database

**FIGURE 3:**
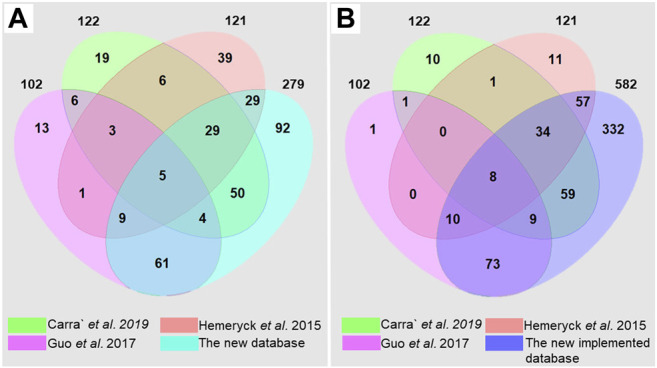
Venn diagrams obtained by comparison of the DNA adduct databases of (Carrà et al., 2019a) (Hemeryck et al., 2015), (Guo et al., 2017) and the current database, **(A)** built upon literature search and **(B)** after implementation with suspected DNA adducts.

The current database fills a critical gap in the DNA adductomics field. Previous works have gathered significant information from the literature for building DNA adduct databases (Hemeryck et al., 2015; Guo et al., 2017; Carrà et al., 2019a). Hemeryck et al., reported 121 different diet-related DNA adducts in their in-house database. These were mainly DNA adducts originating from DNA alkylation, oxidation and lipoxidation, listed along with their relevant origin (Hemeryck et al., 2015). Carra’ et al. created a database of known endogenous DNA adducts deriving from alkylation, LPO and ROS. A total of 122 DNA adducts were reported in their database, including information on adducts name, chemical formula, [M + H]^+^ mass, origin, literature reference and chemical structure (Carrà et al., 2019a). Finally, Guo et al. built a database including 102 known bulky hydrophobic DNA adducts, mainly deriving from HAAs, AAs, PAHs and others. The DNA adducts were reported with their structure, their formula, the monoisotopic mass of the precursor [M + H]^+^ and the major fragment [M + H-dR]^+^, and literature reference (Guo et al., 2017). This represents substantial initial work, but these databases include only a few classes of DNA adducts. The comprehensive database reported here (Supplementary Table S3 and The DNA adduct database in Word format) builds on previous works and reviews to include as many of the previously observed DNA adducts as possible. The literature search led to the identification of a total of 279 DNA adducts coming from different exogenous and endogenous exposures and especially focused on, but not limited to, diet and life-style habits. A comparison of the three previously mentioned databases and the current database has been carried out and reported in Figure 3A and in Supplementary Table S4 in the Supplementary materials.

Due to the fact that the different databases are focusing on different classes of DNA adducts, they show a relatively limited overlap with each other (Figure 3A). The overlap of the current database with the other three DNA adduct databases is instead considerable, and 92 additional DNA adducts have been retrieved from the literature. Fifty-four DNA adducts present in the previous databases are not included in the current database, since several DNA adducts were not found or were not identified at a sufficient level of confidence. Although relatively strict criteria were established for building the initial database (The DNA adduct database in Word format), some of the DNA adducts found in the literature but not sufficiently characterized were still included as suspected DNA adducts in the .xlsx database (The DNA adduct database in Excel format). In addition, all the possible combinations of genotoxicants with the four nucleobases were generated, by taking into consideration chemical plausibility. After implementation, a comprehensive database including 582 DNA adducts was obtained, with more than 300 new DNA adducts (Figure 3B), meaning that this database will help in the identification of new DNA adducts having masses not reported or predicted yet, ultimately expanding the current knowledge on DNA adductomics. Some of the adducts reported in the other three databases were still not reported since they belong to classes that we decided to exclude from the database. We believe that these classes deserve a separate systematic work in the future (Stornetta et al., 2015; Ma et al., 2018; Hu et al., 2019). There are very likely additional DNA adducts not retrieved from this literature search, or that were not yet included because of insufficiently characterization. These adducts along with cyclic adducts, crosslinks, phosphate conjugated DNA adducts (Stornetta et al., 2015; Yu et al., 2016; Ma et al., 2018; Hu et al., 2019) and DNA adduct-repair products (Cooke et al., 2018), can be future additions to the DNA adduct database by the authors or other contributors in the community. The DNA adductome changes over time due to our increasing understanding of DNA adducts and their importance in toxicity and cancer development. Also, the higher number of compounds in exposome databases compared to the lower number of DNA adducts suggests a need for systematic untargeted approaches by including more and more DNA adducts into the overall DNA adductome chemical space (Guo et al., 2017; Cooke et al., 2018; Wilson et al., 2019; Guidolin et al., 2021; Sousa et al., 2021). The database described here should be intended as a first version of the DNA adduct database and spectral library proposed by Guo et al., where thirty-six collaborators worldwide agreed to provide DNA standards to populate the DNA adductome database (Guo et al., 2020). The current database is open-source and is projected as a constantly developing tool where other authors can, upon authorization from the administrators, add new DNA adducts, or additional information on physico-chemical properties (e.g., recorded spectra) or biological information such as induced genomic mutations and disease relationship with associated references. Throughout its usage and further development, research groups should be able to benefit from this database as a major advantage for the investigation of DNA adducts.

## 5 Conclusion

In this paper we established a database to be used for screening of DNA adducts in biological samples using untargeted HRMS, and thus providing a resource for chemical annotation of the DNA adductome. The database was built by aggregating and curating existing DNA adduct databases, and integrating with DNA adducts found through an extensive literature search. The current database contains a systematic collection of DNA adducts, where names, structures and sources have been harmonized and manually curated. The database includes 279 adducts coming from 16 genotoxicant classes and nine sources or processes. Information on the source of the DNA adducts, the samples where they have been detected and the technique that has been used for identification, provides useful metadata to verify the chemical and biological plausibility of annotations. Implementation of the database with new combinations of genotoxicants and nucleobases generated 303 new entries, thereby providing a comprehensive database of 582 DNA adducts to support identification of unknown DNA adducts. Information on structure, molecular formula, monoisotopic mass, and *in silico* predicted fragments for all the 582 entries are provided along with authentic MS/MS spectra for 15 DNA adduct reference standards. The database provides data with computer readable identifiers (SMILES, IUPAC, InChI, InChIKey) presented in publicly available interactive and searchable data tables, which can be easily updated with new entries, new spectra and more detailed metadata in the repository (https://gitlab.com/nexs-metabolomics/projects/dna_adductomics_database). The full development of this database and its integration with MS/MS spectra and informatics tools will allow DNA adductomics to play a major role in systems toxicology, cancer genotoxicity, and cancer prevention.

## Data Availability

The datasets presented in this study can be found in the GitLab repository (https://gitlab.com/nexs-metabolomics/projects/dna_adductomics_database).
