# Supplementary material for: A Comprehensive Database for DNA Adductomics
Source: Front Chem. 2022 May 27;10:908572. doi: 10.3389/fchem.2022.908572 (PMC9184683; doi:10.3389/fchem.2022.908572)
Supplement: Supplementary file 2 [file DataSheet1.docx]

Supplementary Material

# Supplementary Figures and Tables

## Supplementary Figures


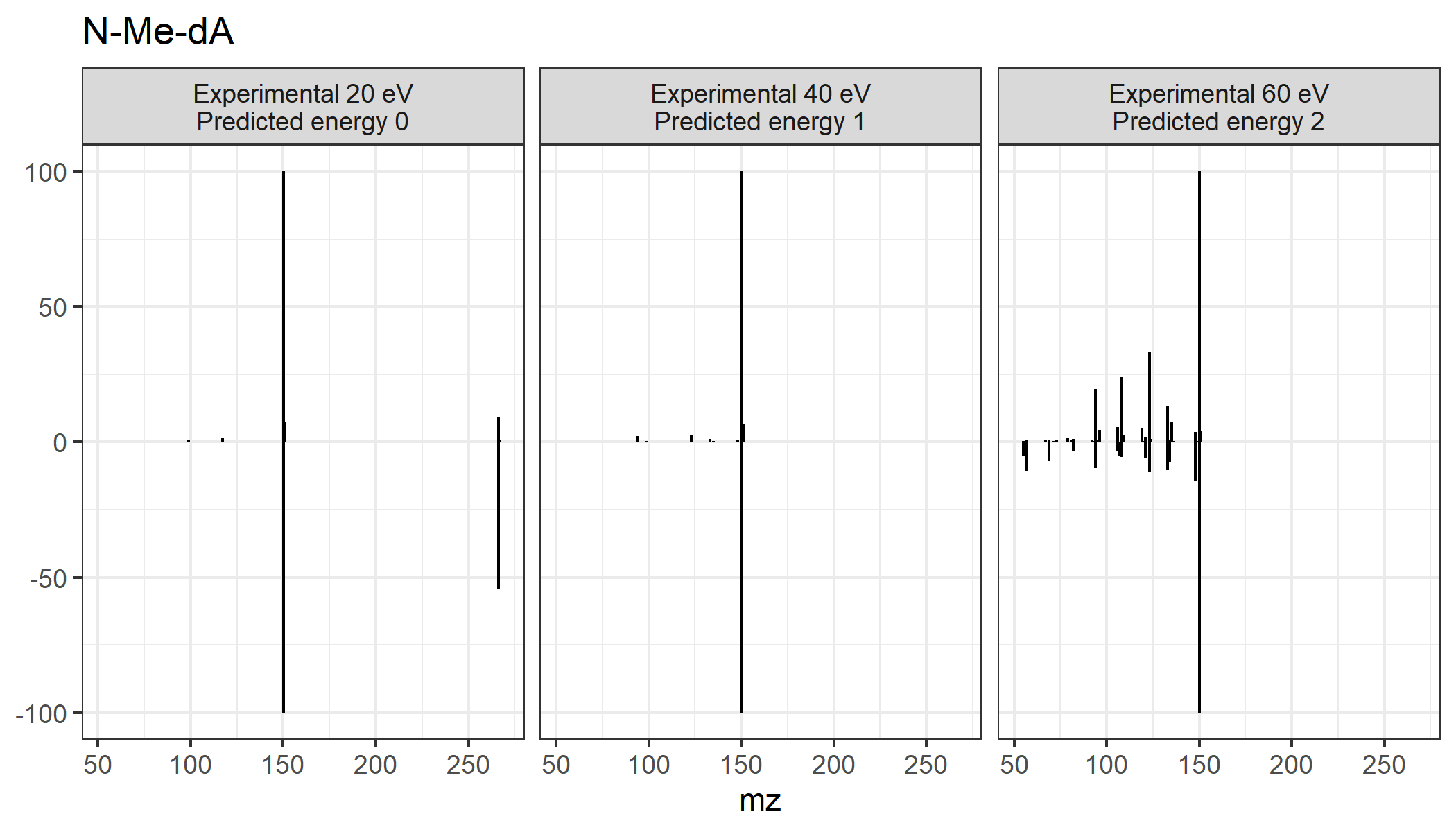


**Supplementary Figure 1.** Comparison of the experimental MS/MS spectra of N^6^-Methyl-dA obtained at 20, 40 and 60 eV, with the predicted fragmentation spectra generated by using CFM-ID.


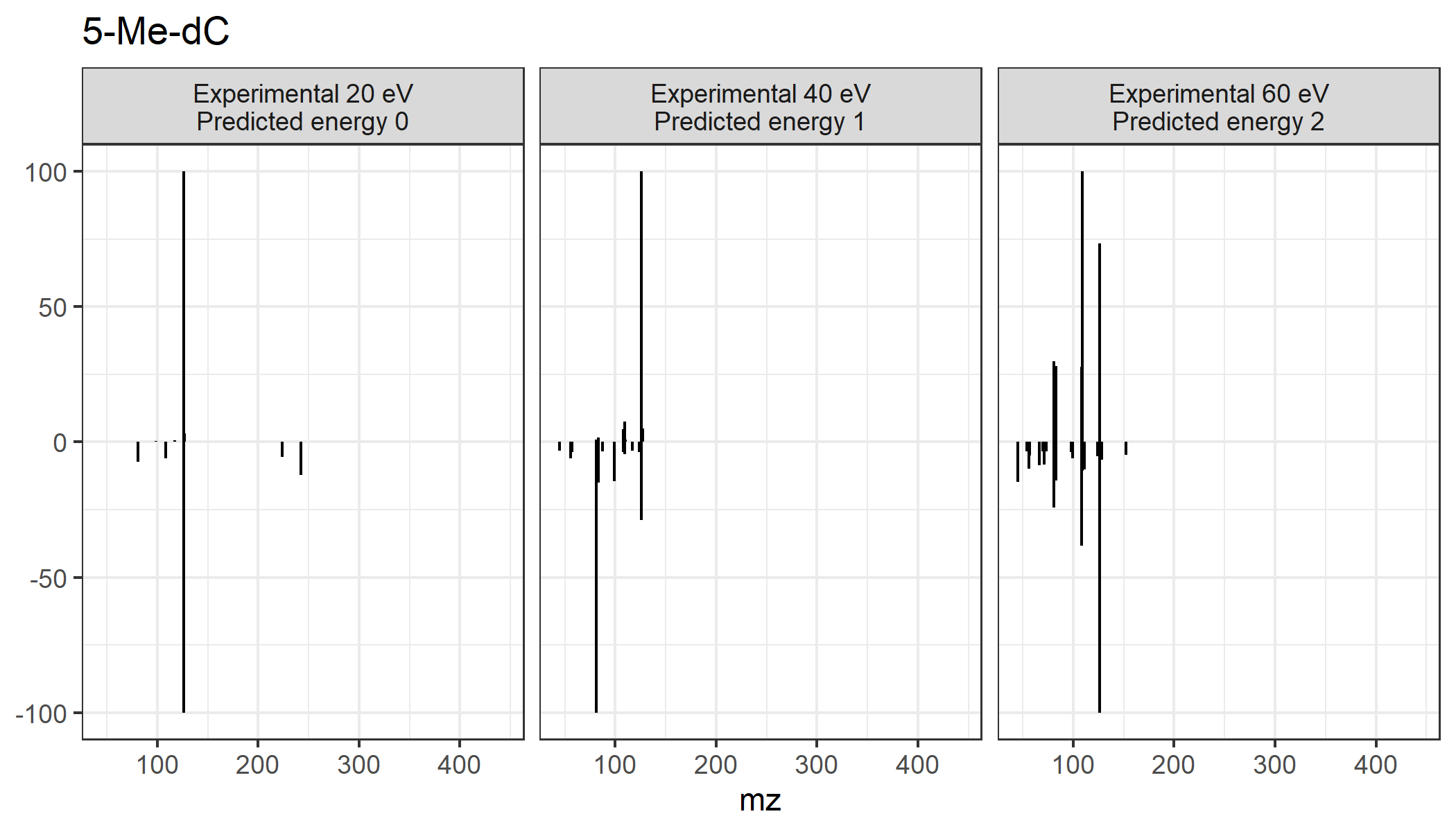
 **Supplementary Figure 2.** Comparison of the experimental MS/MS spectra of 5-Methyl-dC obtained at 20, 40 and 60 eV, with the predicted fragmentation spectra generated by using CFM-ID.


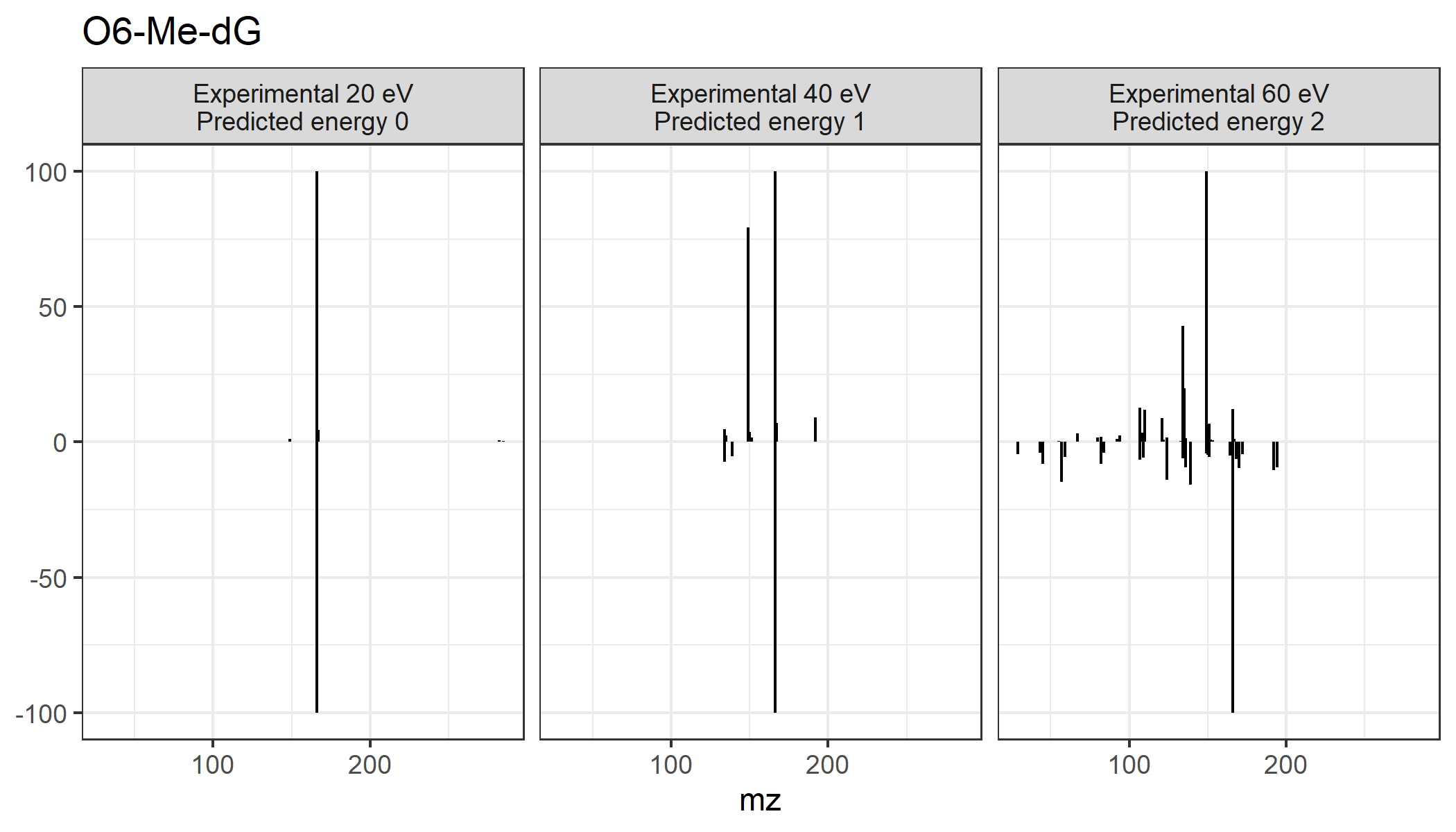
 **Supplementary Figure 3.** Comparison of the experimental MS/MS spectra of O^6^-Methyl-dG obtained at 20, 40 and 60 eV, with the predicted fragmentation spectra generated by using CFM-ID.


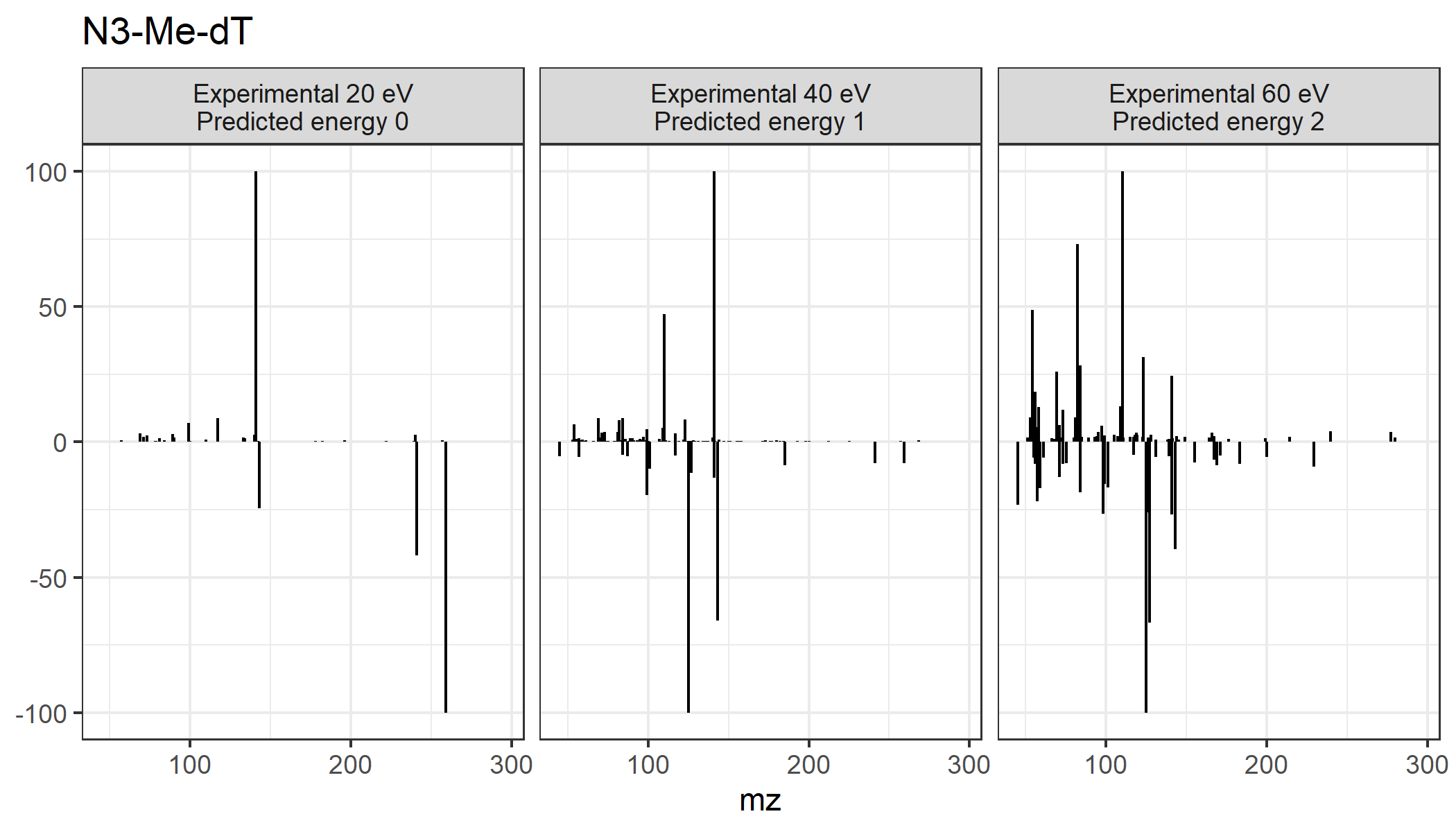
 **Supplementary Figure 4.** Comparison of the experimental MS/MS spectra of 3-Methyl-dT obtained at 20, 40 and 60 eV, with the predicted fragmentation spectra generated by using CFM-ID.


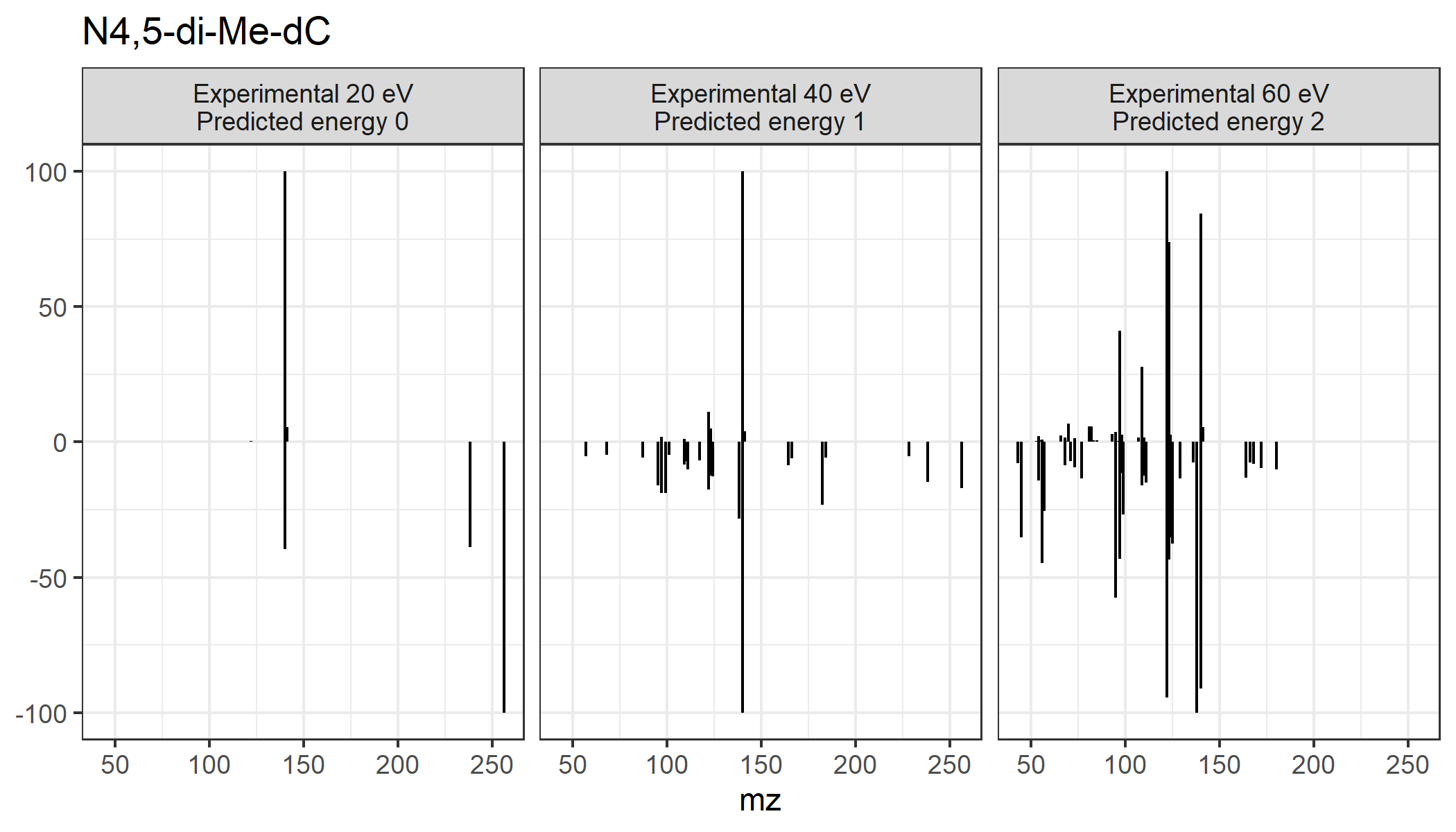
 **Supplementary Figure 5.** Comparison of the experimental MS/MS spectra of N^4^,5-DiMethyl-dC obtained at 20, 40 and 60 eV, with the predicted fragmentation spectra generated by using CFM-ID.


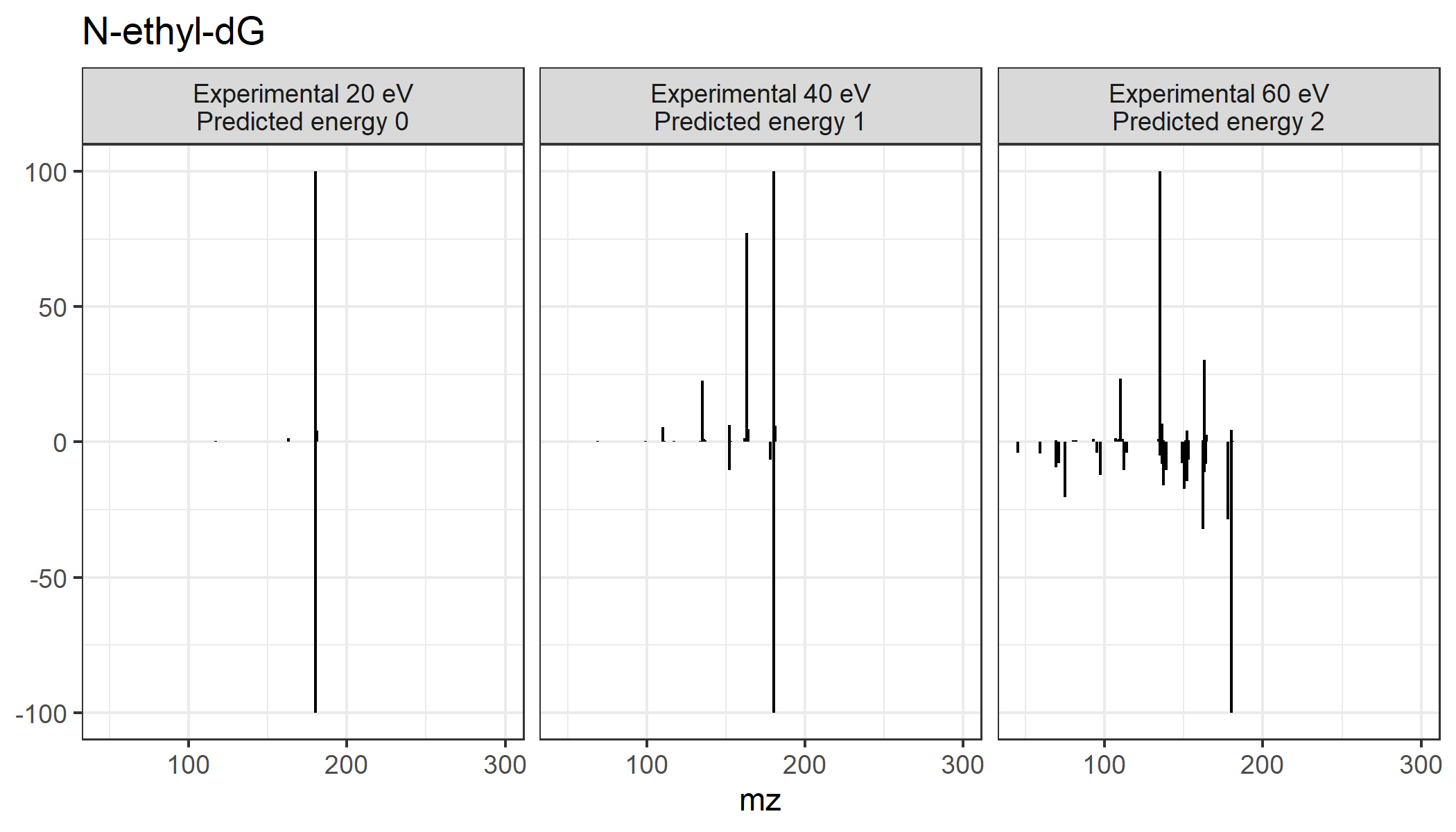
 **Supplementary Figure 6.** Comparison of the experimental MS/MS spectra of N^2^-ethyl-dG obtained at 20, 40 and 60 eV, with the predicted fragmentation spectra generated by using CFM-ID.


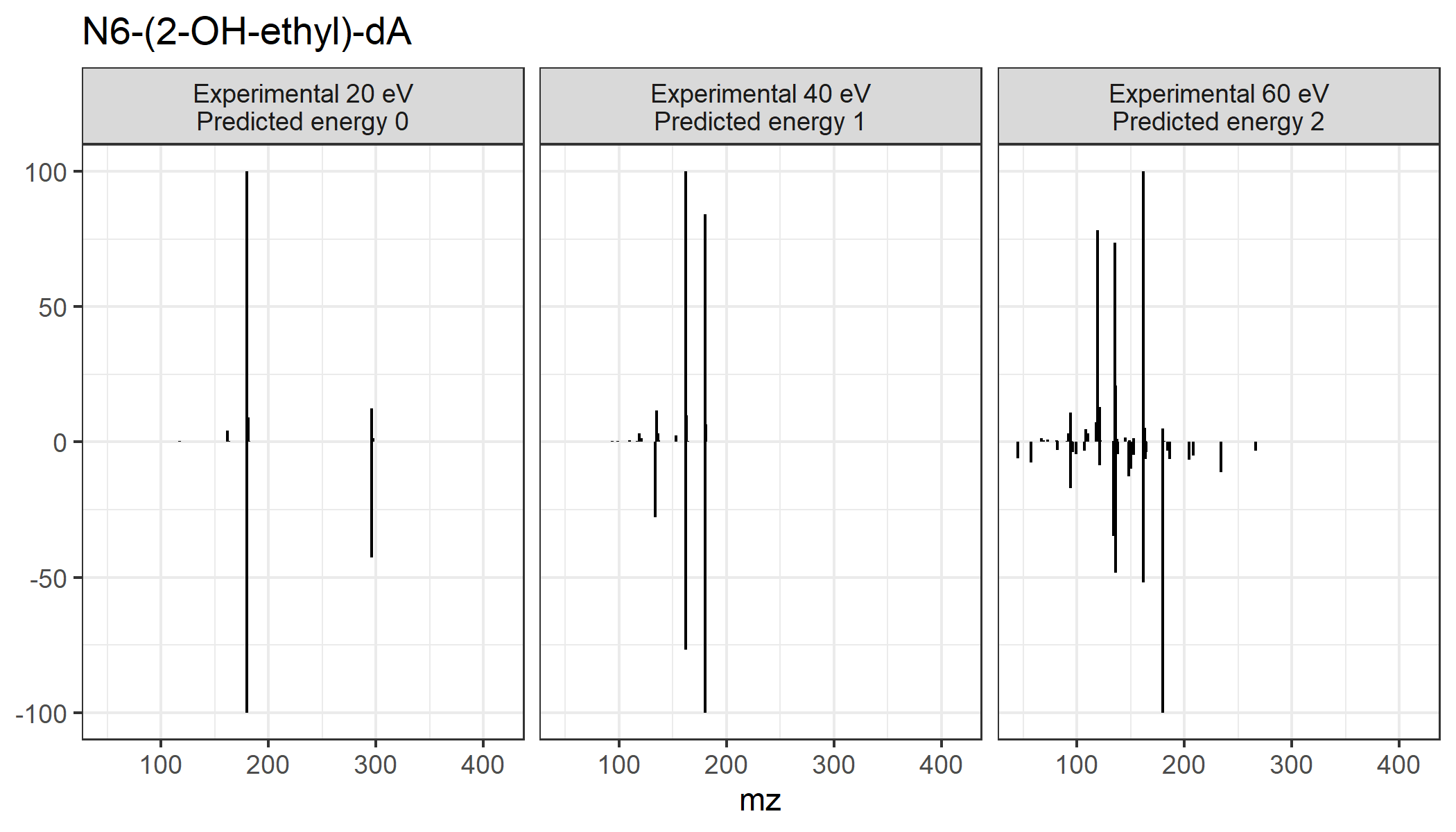
 **Supplementary Figure 7.** Comparison of the experimental MS/MS spectra of N^6^-(2-OH-ethyl)-dA obtained at 20, 40 and 60 eV, with the predicted fragmentation spectra generated by using CFM-ID.


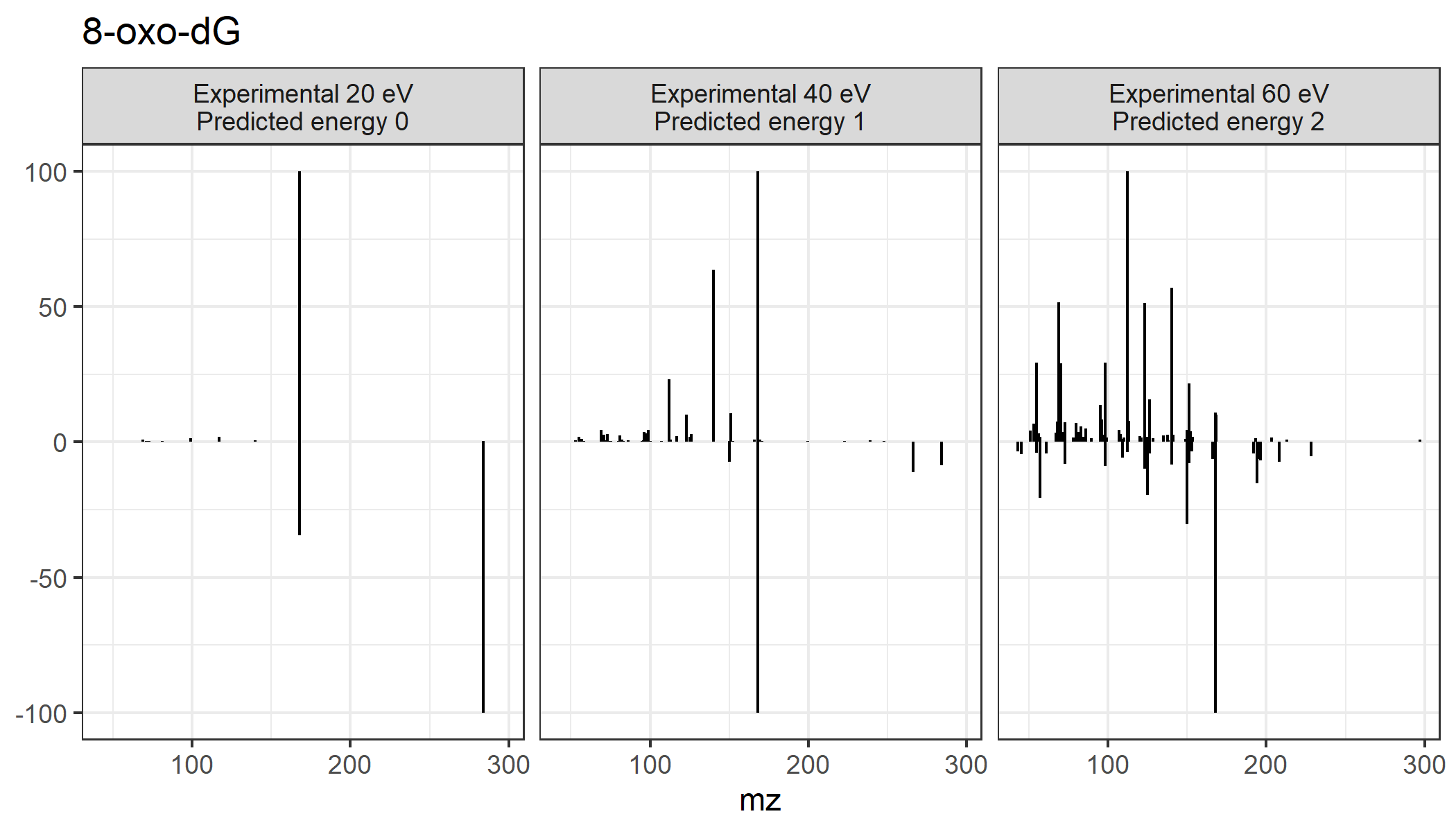
 **Supplementary Figure 8.** Comparison of the experimental MS/MS spectra of 8-oxo-dG obtained at 20, 40 and 60 eV, with the predicted fragmentation spectra generated by using CFM-ID.


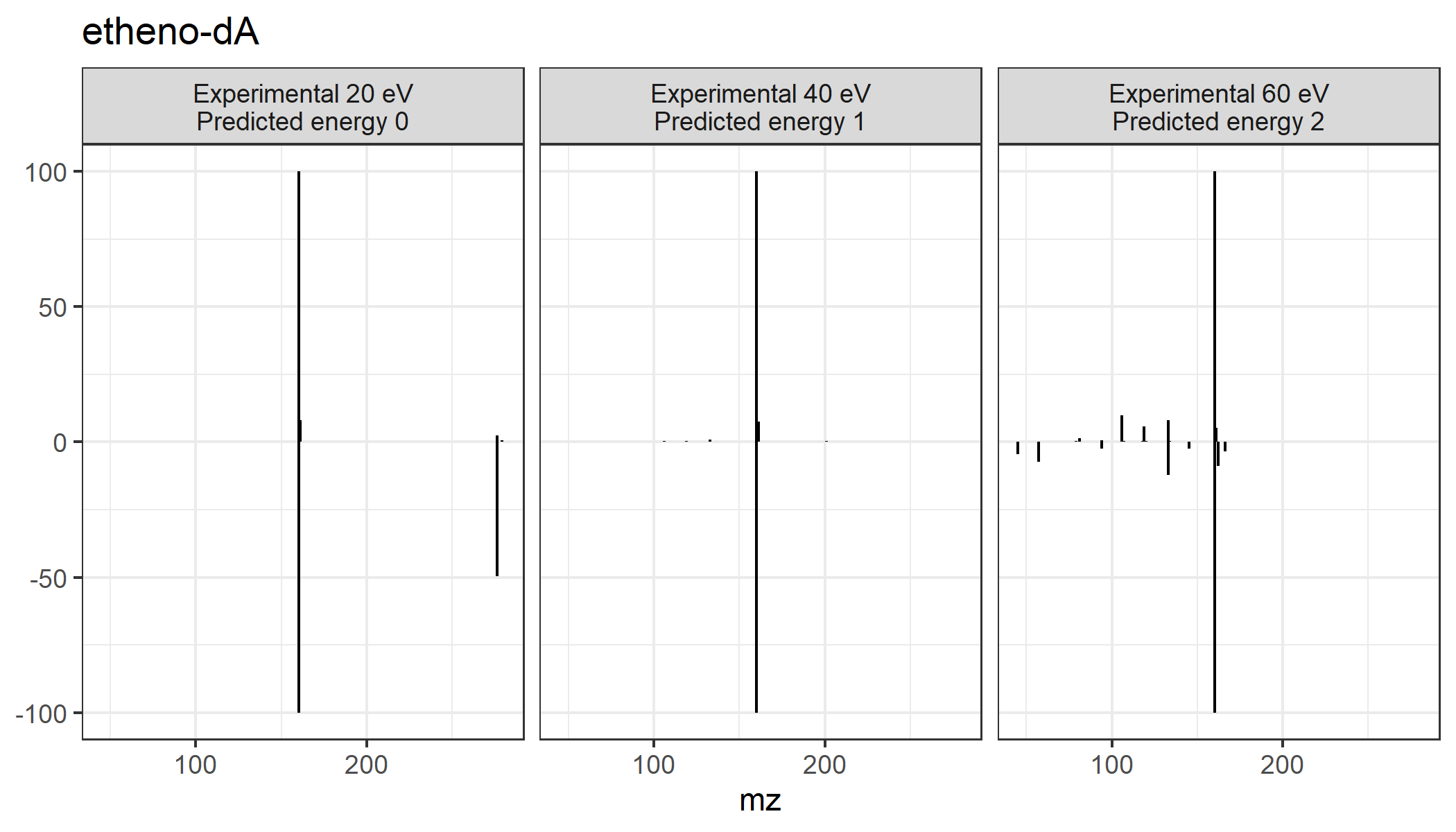
 **Supplementary Figure 9.** Comparison of the experimental MS/MS spectra of 1,N^6^-ε-dA obtained at 20, 40 and 60 eV, with the predicted fragmentation spectra generated by using CFM-ID.


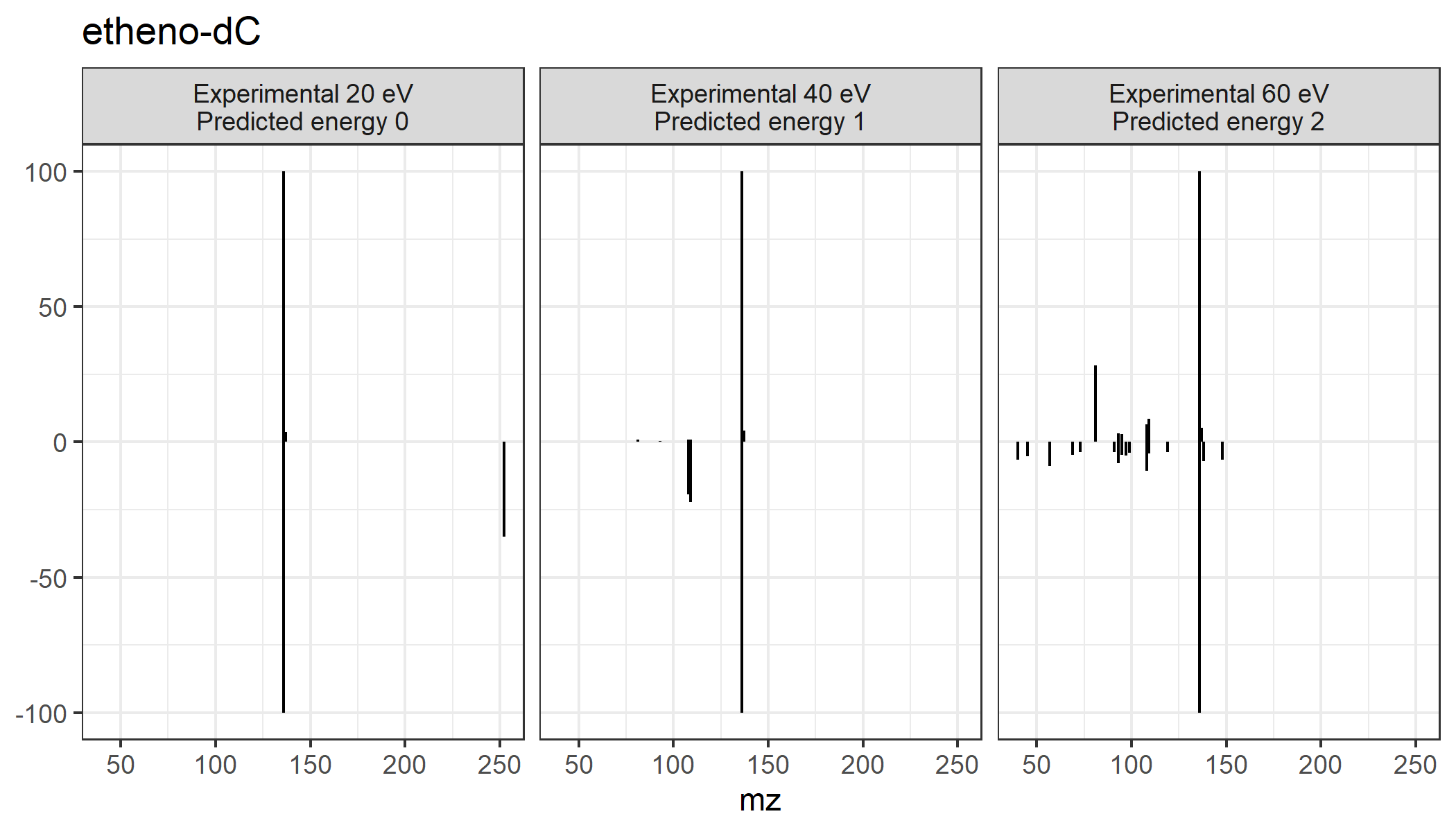
 **Supplementary Figure 10.** Comparison of the experimental MS/MS spectra of 3,N^4^-ε-dC obtained at 20, 40 and 60 eV, with the predicted fragmentation spectra generated by using CFM-ID.


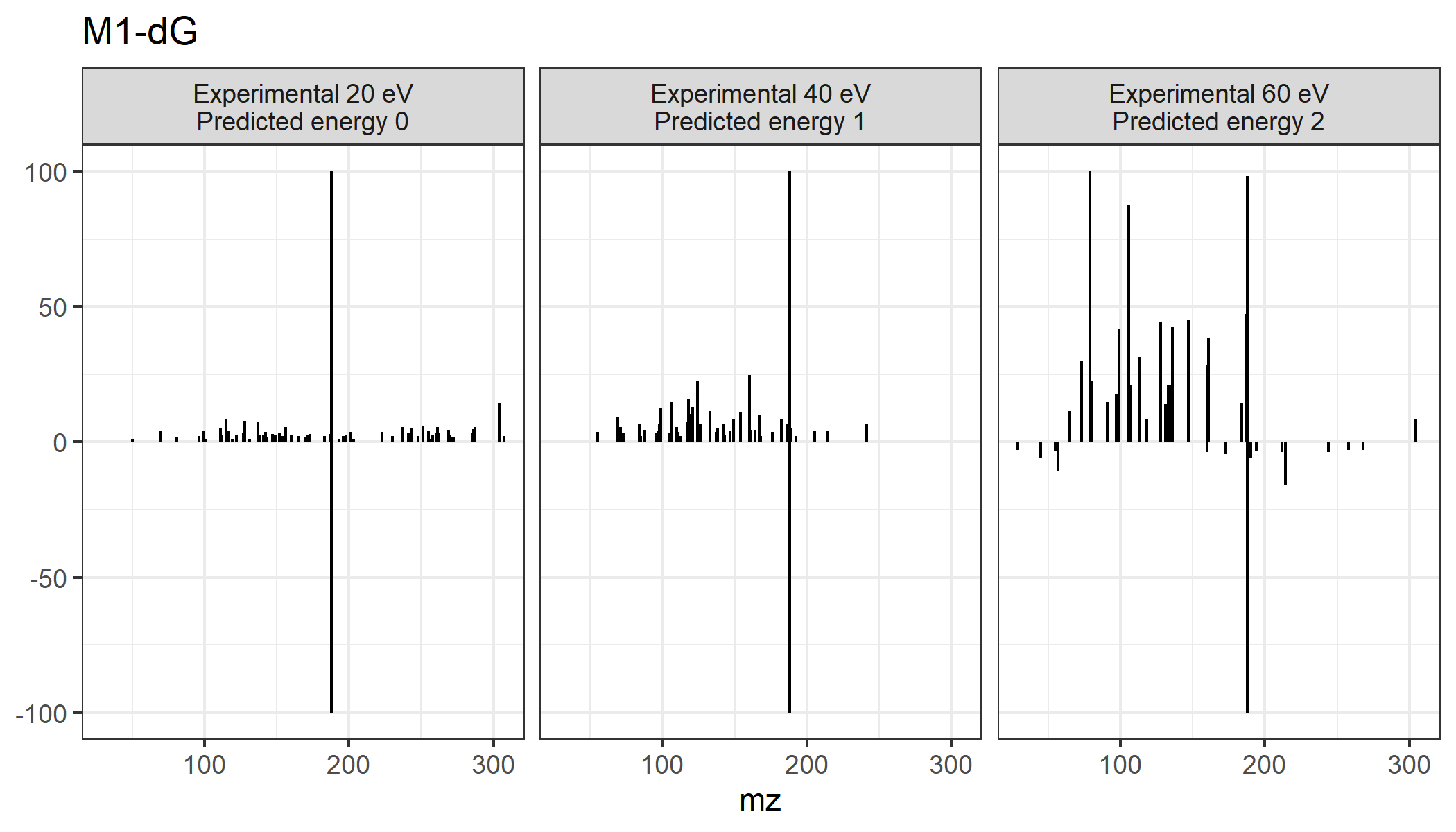
 **Supplementary Figure 11.** Comparison of the experimental MS/MS spectra of M1-dG obtained at 20, 40 and 60 eV, with the predicted fragmentation spectra generated by using CFM-ID.


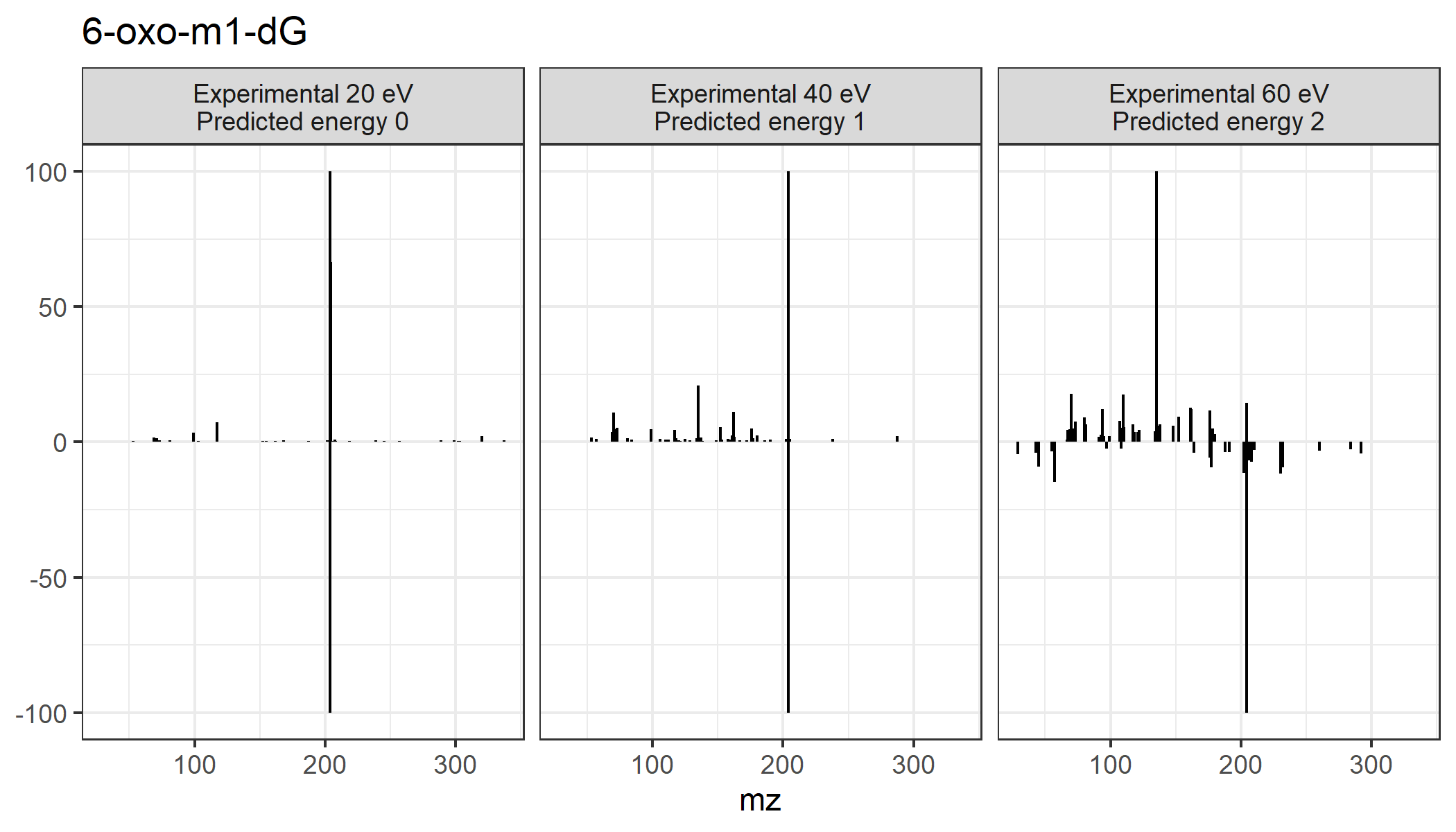
 **Supplementary Figure 12.** Comparison of the experimental MS/MS spectra of 6-Oxo-M1-dG obtained at 20, 40 and 60 eV, with the predicted fragmentation spectra generated by using CFM-ID.


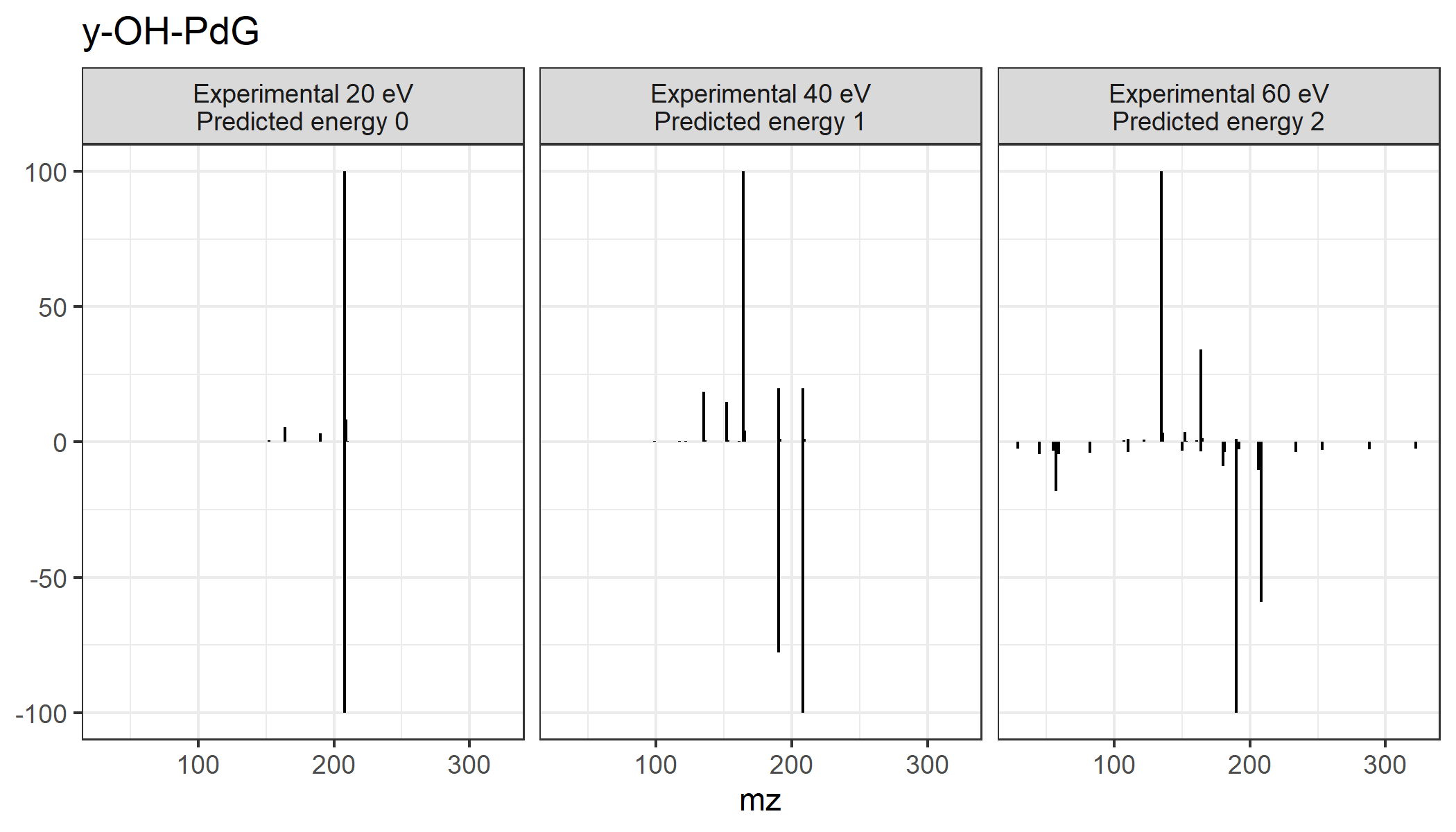


**Supplementary Figure 13.** Comparison of the experimental MS/MS spectra of 1,N^2^-γ-(OH-P-dG=Acr 1I-dG obtained at 20, 40 and 60 eV, with the predicted fragmentation spectra generated by using CFM-ID.


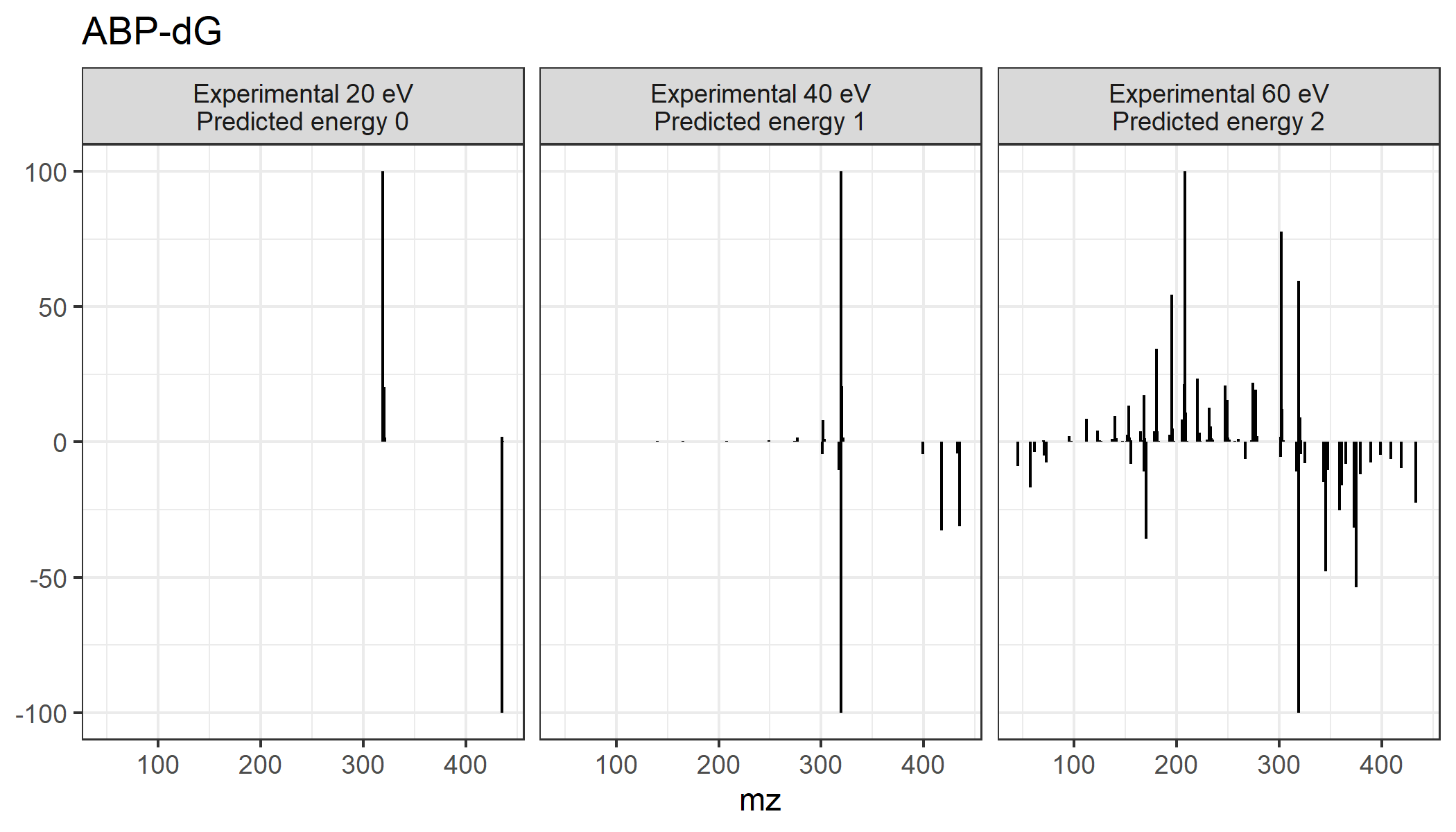
 **Supplementary Figure 14.** Comparison of the experimental MS/MS spectra of 8-ABP-dG obtained at 20, 40 and 60 eV, with the predicted fragmentation spectra generated by using CFM-ID.


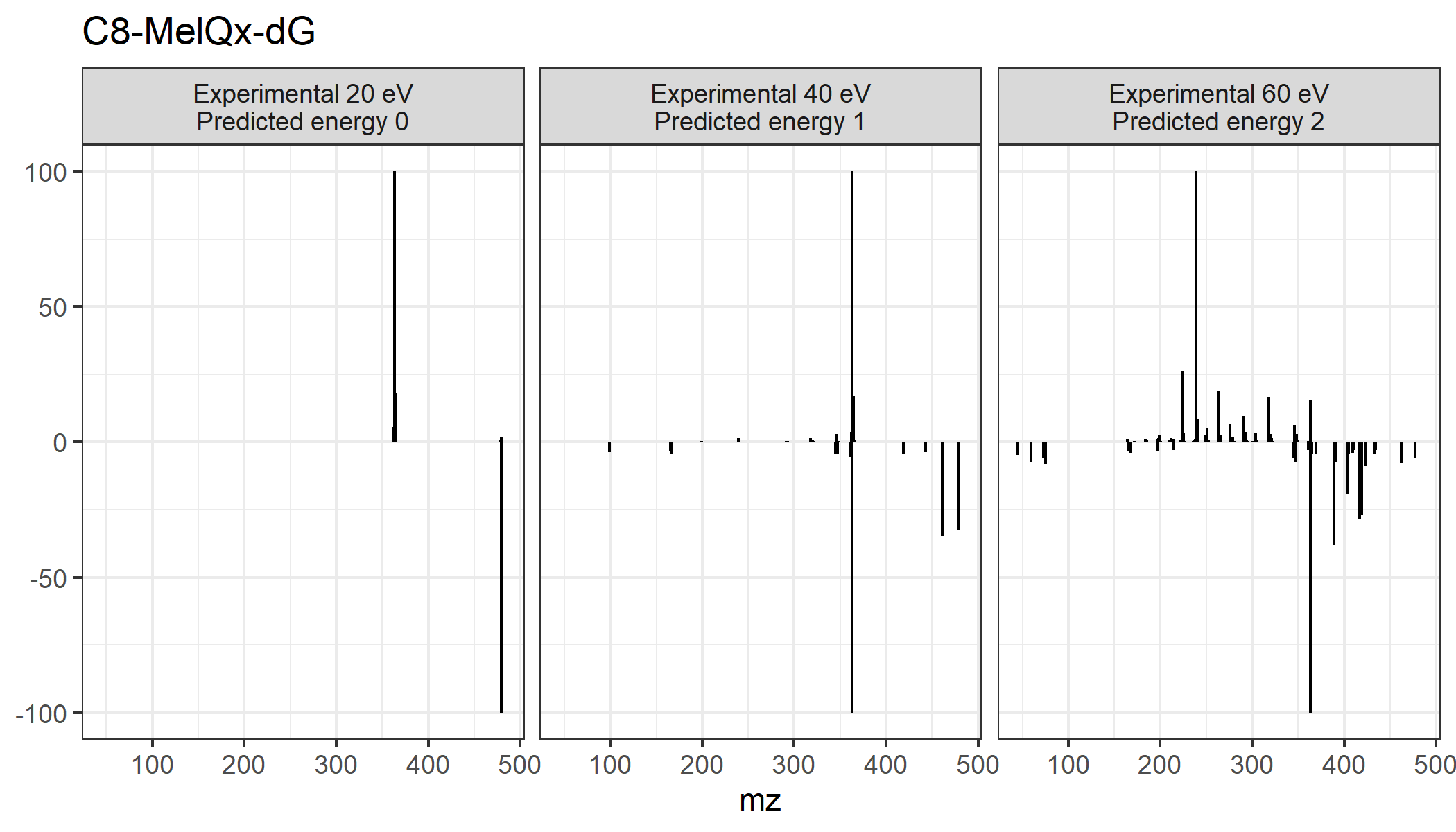
 **Supplementary Figure 15.** Comparison of the experimental MS/MS spectra of 8-MeIQx-dG obtained at 20, 40 and 60 eV, with the predicted fragmentation spectra generated by using CFM-ID.

## Supplementary Tables

**Supplementary Table S1:** Rules for assigning DNA adduct names.

|  | **Rule** | **Example** |
| --- | --- | --- |
| 1 | The nucleosides are always expressed at the end of the name, i.e. after the modification. | Me-dA, Me-dG, Me-dC, Me-dT, Me-dU |
| 2 | The different isomeric DNA adducts are reported by indicating the position where the modification occurs, as follows: i) if the substitution occurs on an atom within the heterocycle, a number is introduced as prefix, ii) if the substitution occurs on a N or O outside the heterocycle, the N or O with the position in the apex is introduced as prefix. | N^6^-Me-dA  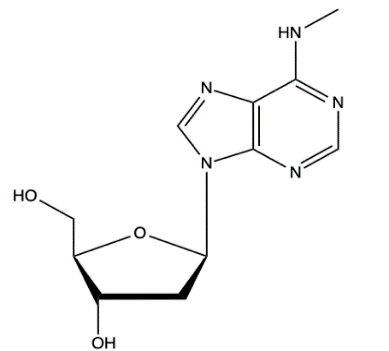  2-Me-dA   |
| 3 | The different isomeric forms of the modification are expressed in brackets | 8-(1-Hydroxy-ethyl)-dG  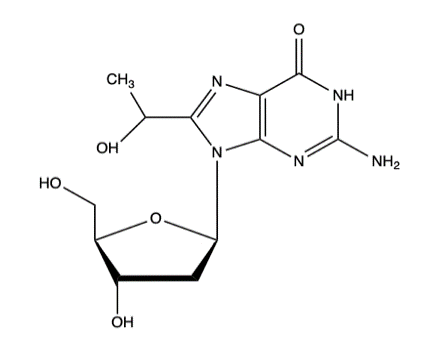  7-(2-Hydroxy-ethyl)-dG  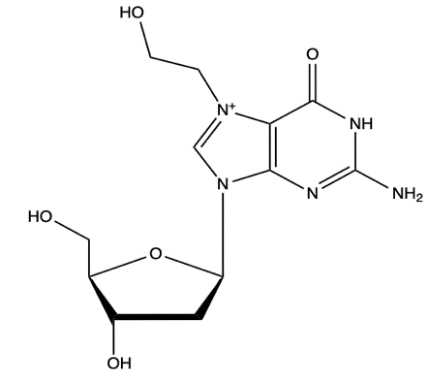 |
| 4 | The different atoms or positions in which a modification can bind to the nucleobase are expressed in the brackets. In particular if the modification binds through a N atom this is indicated as N´. | 8-(N’-2Methylaniline)-dA    N^6^-(2Methylaniline)-dA   |
| 5 | For the DNA adducts in which the core of the nucleobase has been modified, the original nucleobase is specified in the column “DNA adduct identity”.  Exceptions: AFB1-FAPY-dG, FAPY-dG and FAPY dA. | deoxyxanthosine, from dG; alloxan, from dC; hydroxy-methyl-hydantoin from dT; |
| 6 | The following abbreviations are used for all the DNA adducts: hydroxy (-OH), Methyl (-Me), Ethyl (-Et), Propyl (-Pr), Butyl (-But), benzyl (-Bz), Acetyl (-Ac), propano (P), Methoxy (MeO), Ethoxy (EtO). |  |
| 7 | When a nucleobase is modified by addition of a keto group (C=O) the prefix Oxo is introduced into the name. | oxo-dG  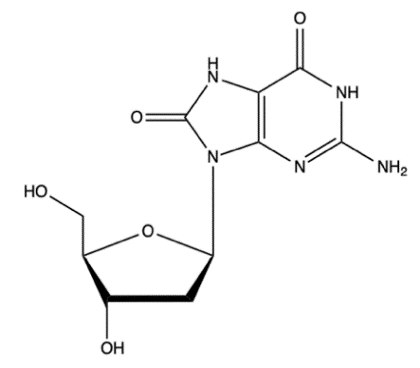 |
| 8 | When two identical groups are substituted on a nucleobase the prefix, -di is used. | Di-methyl-dC |
| 9 | When a double bond of the nucleobase is saturated by adding one or two extra H, the adduct is called hydro or dihydro, respectively. | 5-OH-5,6-di-hydro-dU  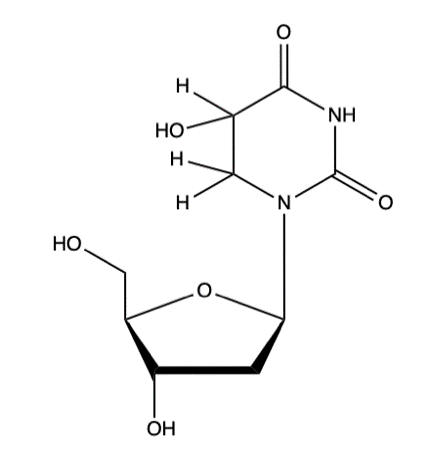 |
| 10 | The systematic rules do not apply when the name or relative abbreviation becomes excessively long, or when a name is commonly accepted in the literature. | Hydroxy-methyl-hydroxy-propano-dG= HMHP not OH-Me-OH-P-dG.  DiBenzo[a,h]anthracenediolepoxide=DB[a,h]ADE not Di-Bz[a,h]ADE.  Dimethylaniline-dG= DMA-dG not Di-Me-aniline-dG |
| 11 | For many DNA adducts the name is chosen according to the causative genotoxicant. | cis-butene dial-dG (cis-BDA-dG), malondialdehyde (M1-dG), oxo-hexenal (OHE-dG) |
| 12 | The adduct deriving from malondialdehyde, acrolein, crotonaldehyde, acetaldehyde and formaldehyde, are numbered according to how many units of aldehyde modify the nucleobase. | 1 unit of malondialdehyde M1-dG  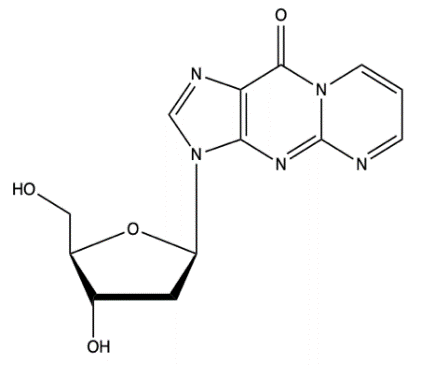  2 units malondialdehyde M2-dG  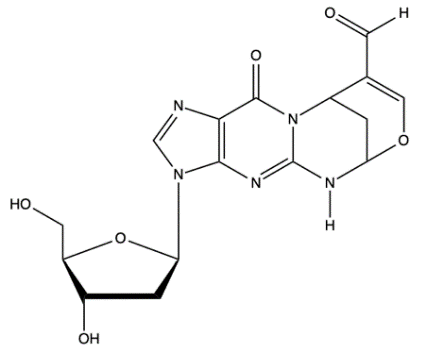  3 units malondialdehyde M3-dA  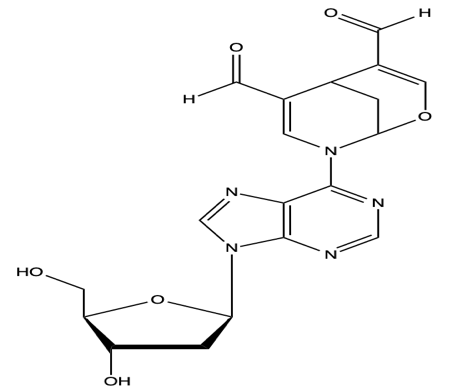 |
| 13 | When the modification from an aldehyde results in different isomers the adducts are differentiated with roman numbers. | M2AAI-dG  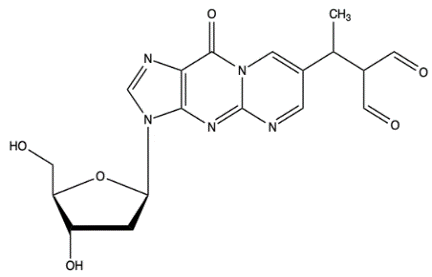  M2AAII-dG  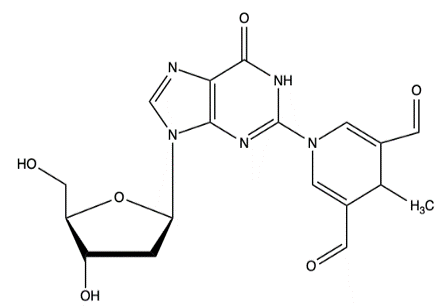  Both 2 units of malondialdehyde and one of acetaldehyde, but bound in different ways |
| 14 | The series of adducts deriving from Acrolein and Crotonaldehyde are named according to rule 12 and 13. However an alternative name is proposed since many names are commonly used in the literature. | Acr-1I-dG = 1,N^2^-γ-(OH-P)-dG  Cro-1I-dG = 1,N^2^-(Me-OH-P)-dG  Cro-2II-dG= N^2^-Paraldol-dG |
| 15 | When there is formation of an imidazole ring with two of the nitrogen atoms from the nucleobase the DNA adducts are called, “etheno”. | Etheno-dA  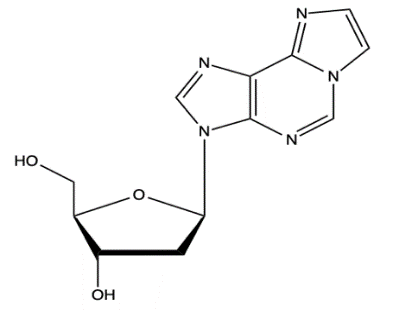 |
| 16 | The DNA adducts deriving from different α,β unsaturated aldehydes are called with the name of the original aldehyde, but an alternative name is kept (applying the systematic rules for etheno DNA adducts). | Name: ONE-dG  Alternative name: heptanone-etheno-dG  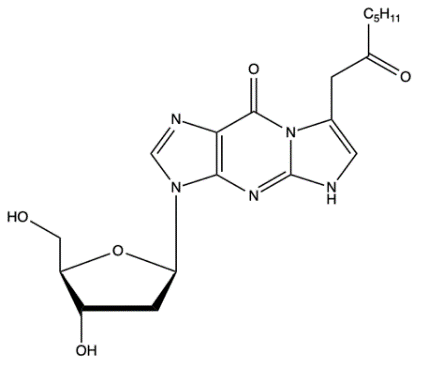 |
| 17 | For adducts that show tautomeric forms, the most reported stable tautomer is reported. | 8-oxo-dG not 8-OH-dG  glyoxal–dG not OH-acetyl-dG  M1-dG not oxopropenyl-dG |

**Supplementary Table S2 :** Keyword combinations in the literature search

| **Search Term** | **AND** | **AND** | **NOT** | **Limited to** | **Pubmed (All Fields)** | **Scopus  (Title, Abstract, Keywords)** | **Included in the table** | **nr of adducts** |
| --- | --- | --- | --- | --- | --- | --- | --- | --- |
| 1. Preliminary search of databases | | | | | | | | |
| Database OR Screening | DNA adduct |  |  | 2010-2020, English | 390 | 377 |  |  |
| 2. Extensive search of reviews | | | | | | | | |
| Cancer OR carcinogenesis | DNA adduct | Review |  | 2010-2020, English | 88 | 423 |  |  |
| 3. Literature search on genotoxicant classes/sources of DNA adducts | | | | | | | | |
| DNA adduct OR thymine OR thymidine OR cytosine OR deoxycytidine OR guanine OR deoxyguanosine OR adenine  OR deoxyadenosine | Mycotoxin | Cancer OR Carcinogensis | Review | English | 463 | 93 | 4 | 5 |
|  | Pyrrolizidine alkaloid |  |  | English | 25 | 32 | 1 | 4 |
|  | Aromatic amines |  |  | English | 229 | 265 | 23 | 50 |
|  | Furan |  |  | English | 189 | 184 | 3 | 7 |
|  | N-nitroso compound |  |  | English | 87 | 69 | 26 | 38 |
|  | Polycyclic aromatic hydrocarbons |  |  | English | 574 | 945 | 8 | 23 |
|  | Acrylamide |  |  | English | 125 | 199 | 1 | 4 |
|  | Aldehyde |  |  | English | 929 | 691 | 38 | 56 |
|  | Alcohol |  |  | English | 2042 | 1043 | 31 | 31 |
|  | Tobacco |  |  | English | 689 | 795 | 125 | 137 |
|  | Pollution |  |  | English | 717 | 314 | 58 | 125 |
|  | Heated food |  |  | English | 27 | 2 | 15 | 26 |
|  | Red meat OR processed meat |  |  | English | 161 | 61 | 35 | 50 |
|  | Herbs OR spices |  |  | English | 77 | 351 | 9 | 21 |

**Supplementary Table S3:** DNA adduct database built after literature search. The columns contain the following information about DNA adducts: 1) identity: the name proposed in the new database, the name abbreviation, the molecular formula, the monoisotopic mass and the alternative name when needed; 2) structure; 3) original genotoxicant and possible source; 4) matrix and type of study i.e. *in vitro*, *in vivo* (animal) or human study and relative reference; 5) analytical technique and relative reference.

|  | **DNA adduct identity^a^** | **DNA adduct structure** | **DNA adduct source^b^** | **Matrix and type of study** | **Analytical technique^c^** | |  |
| --- | --- | --- | --- | --- | --- | --- | --- |
| 1 | dI  deoxyinosine  C_10_H_12_N_4_O_4_  252.08585  From dA |  | RNS, ROS  Endogenous sources | [1] Hsu et al. 2009:  Human urine  [2]Pang et al. 2007: Mouse spleen, liver and kidney tissue  [3]Mangerich et al. 2012:  Mouse liver and colon tissue | [1] Hsu et al. 2009:  LC-MS/MS  [2]Pang et al. 2007:  LC-MS/MS  [3]Mangerich et al. 2012:  LC-MS/MS | |  |
| 2 | dX  deoxyxanthosine  C_10_H_12_N_4_O_5_  268.080771  From dG | 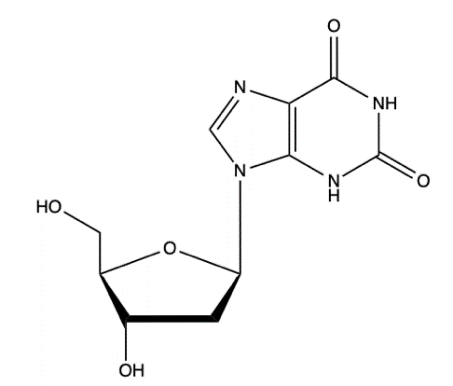 | RNS, ROS  Endogenous sources | [2]Pang et al. 2007:  Mouse spleen, liver and kidney tissue  [3]Mangerich et al. 2012:  Mouse liver and colon tissue | [2]Pang et al. 2007:  LC-MS/MS  [3]Mangerich et al. 2012:  LC-MS/MS | |  |
| 3 | dU  Deoxyuridine  C_18_H_12_N_2_O_5_  228.074623  From dC | 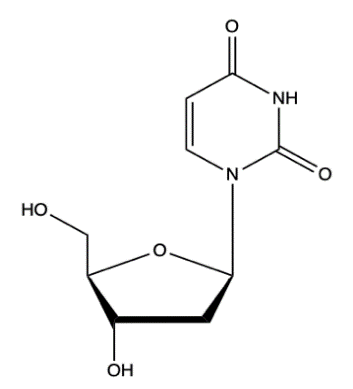 | RNS, ROS  Endogenous sources | [1] Hsu et al. 2009:  Human urine  [2]Pang et al. 2007:  Mouse spleen, liver and kidney tissue | [1] Hsu et al. 2009:  LC-MS/MS  [2]Pang et al. 2007:  LC-MS/MS | |  |
| 4 | 2-Amino-imidazolone  C_8_H_12_N_4_O_4_  228.085856  From dG | 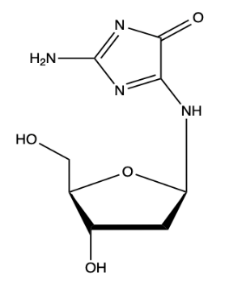 | RNS, ROS  Endogenous sources | [4]Cadet et al. 2004:  Oxidation of dG (*in vitro*) | [4]Cadet et al. 2004:  LC-MS and NMR | |  |
| 5 | Oxazolone  C_8_H_14_N_4_O_5_  246.096421  From dG |  | ROS  Endogenous sources, alcohol | [4]Cadet et al. 2004:  Oxidation of dG (*in vitro*) | [4]Cadet et al. 2004:  LC-MS and NMR | |  |
| 6 | Guanidinohydantoin  C_9_H_15_N_5_O_5_  273.107320  From dG | 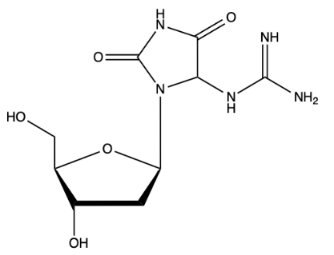 | ROS, LPO  Endogenous sources, red and processed meat, alcohol | [3]Mangerich et al. 2012:  Mouse liver and colon tissue | [3]Mangerich et al. 2012:  LC-MS/MS | |  |
| 7 | Guanidine-nitroimidazole  C_9_H_14_N_6_O_5_  286.102569  From dG | 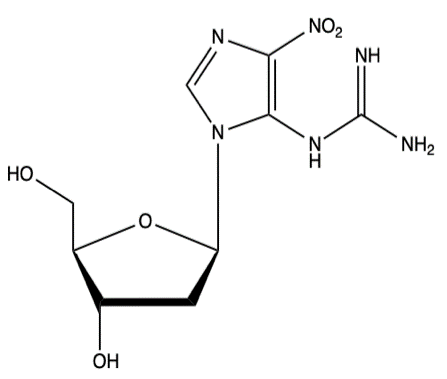 | RNS, ROS, LPO  Endogenous sources, tobacco | [5]Yun et al. 2011:  Calf thymus DNA | [5]Yun et al. 2011:  LC-MS/MS | |  |
| 8 | Spiroiminodihydantoin  C_10_H_13_N_5_O_6_  299.086585  From dG | 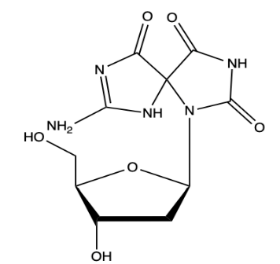 | ROS  Endogenous sources, alcohol | [3]Mangerich et al. 2012:  Mouse liver and colon tissue | [3]Mangerich et al. 2012:  LC-MS/MS | |  |
| 9 | Cyanuric acid  C_8_H_11_N_3_O_6_  245.064791  From dG |  | ROS  Endogenous sources, UV irradiation | [6]Raoul et al 1996:  UV irradiation of dG (*in vitro*) | [6]Raoul et al 1996:  LC-UV and NMR | |  |
| 10 | 5-OH-hydantoin  5-Hydroxyhydantoin  C_8_H_12_N_2_O_6_  232.069538  From dC | 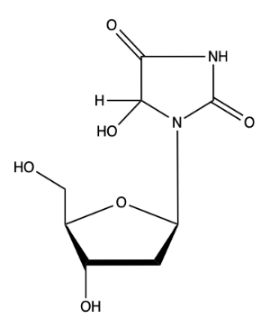 | ROS  Endogenous sources, UV irradiation, alcohol | [7]Girault et al 1996:  Ozonolysis dC (*in vitro*) | [7]Girault et al 1996:  LC-MS and NMR | | |
| 11 | Alloxan  C_9_H_10_N_2_O_7_  258.048803  From dC | 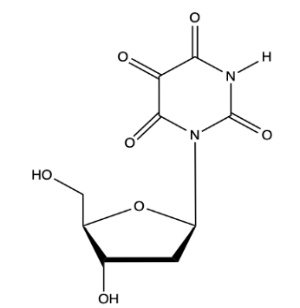 | ROS, LPO  Endogenous sources | [8]Dizdaroglu et al. 1993:  Calf thymus DNA | [8]Dizdaroglu et al. 1993:  GC-MS | | |
| 12 | 5,5-OH-Me-hydantoin  5,5-Hydroxy-methyl-hydantoin  C_9_H_14_N_2_O_6_  246.085188  From dT | 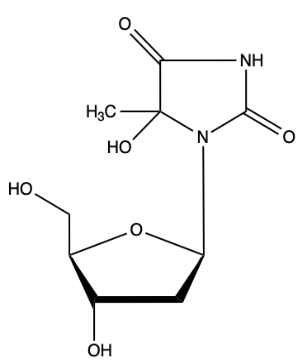 | ROS  Endogenous sources, alcohol, red and processed meat | [8]Dizdaroglu et al. 1993:  Calf thymus DNA  [9]Nackerdien et al. 1992:  Human leukemic cells | [8]Dizdaroglu et al. 1993:  GC-MS  [9]Nackerdien et al. 1992:  GC-MS | | |
| 13 | FAPY-dA  Diamino-formamidopyrimidine  C_10_H_15_N_5_O_4_  269.112405 | 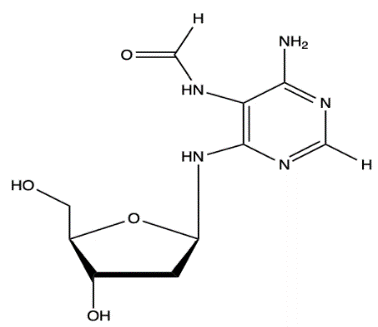 | ROS  Endogenous sources, UV irradiation | [10]Hu et al. 2005:  Mouse liver  [11]Carra` et al. 2019:  Mouse lung tissue | [10]Hu et al. 2005:  GC-MS  [11]Carra` et al. 2019:  2D-LC-HRMS/MS  (tentatively identified) | |  |
| 14 | FAPY-dG  Diamino-hydroxy-formamidopyrimidine  C_10_H_15_N_5_O_5_  285.107320 | 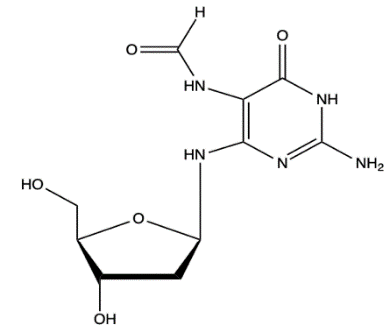 | ROS  Endogenous sources, UV irradiation | [10]Hu et al. 2005:  Mouse liver  [11]Carra` et al. 2019: Mouse lung tissue  [9]Nackerdien et al. 1992:  Human leukemic cells | [10]Hu et al. 2005:  GC-MS  [11]Carra` et al. 2019:  2D-LC-HRMS/MS  (tentatively identified)  [9]Nackerdien et al. 1992:  GC-MS | |  |
| 15 | 8-Nitro-dG  C_10_H_12_N_6_O_6_  312.081833 | 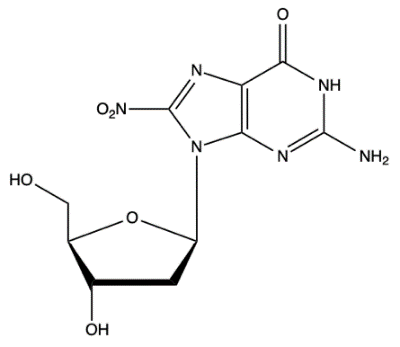 | RNS    Alkylation  Endogenous sources, tobacco | [5]Yun et al. 2011:  Calf thymus DNA | [5]Yun et al. 2011:  LC-MS/MS | |  |
| 16 | 8-Cl-dA  8-Chloro-dA  C_10_H_12_ClN_5_O_3_  285.06287 |  | ROS, halogenation products  Endogenous sources | [12]Badouard 2005:  Human leukemic cells (SKM-1) | [12]Badouard 2005:  LC-MS/MS | | |
| 17 | 8-Cl-dG  8-Chloro-dG  C_10_H_12_ClN_5_O_4_  301.05778 |  | ROS, halogenation products  Endogenous sources | [12]Badouard 2005:  Human leukemic cells (SKM-1) | [12]Badouard 2005:  LC-MS/MS | | |
| 18 | 5-Cl-dC  5-Chloro-dC  C_9_H_12_ClN_3_O_4_  261.05163 |  | ROS, halogenation products  Endogenous sources | [12]Badouard 2005:  Human blood  [3]Mangerich et al. 2012:  Mouse liver and colon tissue | [12]Badouard 2005:  LC-MS/MS  [3]Mangerich et al. 2012:  LC-MS/MS | | |
| 19 | 5-Cl-dU  5-Chloro-dU  C_9_H_11_ClN_2_O_5_  262.03565 |  | ROS, halogenation products  Endogenous sources | [13]Jiang et al 2003:  Rat tissue | [13]Jiang et al 2003:  GC-MS | | |
| 20 | 5-Br-dU  5-Bromo-dU  C_9_H_11_BrN_2_O_5_  305.98513 |  | ROS, halogenation products  Endogenous sources | [14]Henderson et al. 2001:  Human eosinophils | [14]Henderson et al. 2001:  LC-UV/MS and NMR | | |
| 21 | 8-Oxo-dA  C_10_H_13_N_5_O_4_  267.096755  8-OH-dA | 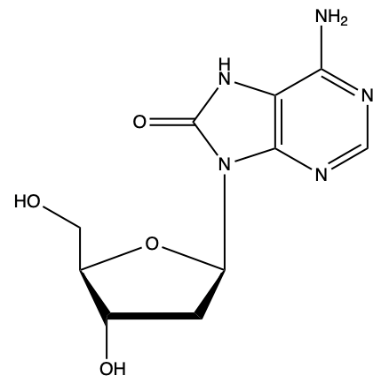 | ROS  Endogenous sources, UV irradiation, alcohol | [15]Ma et al. 2016:  Human retina  [16]Frelon et al.2000:  Calf thymus DNA  [11]Carra` et al. 2019:  Mouse lung tissue | [15]Ma et al. 2016:  LC- MS/MS  [16]Frelon et al.2000: LC-MS/MS  [11]Carra` et al. 2019:  2D-LC-HRMS/MS  (tentatively identified) | |  |
| 22 | 2-OH-dA  2-Hydroxy-dA  C_10_H_13_N_5_O_4_  267.096755 |  | ROS  Endogenous sources, UV irradiation | [9]Nackerdien et al. 1992:  Human leukemic cells  [11]Carra` et al. 2019:  Mouse lung tissue | [9]Nackerdien et al. 1992:  GC-MS  [11]Carra` et al. 2019:  2D-LC-HRMS/MS  (tentatively identified) | |  |
| 23 | 8-Oxo-dG  C_10_H_13_N_5_O_5_  283.091670  8-OH-dG | 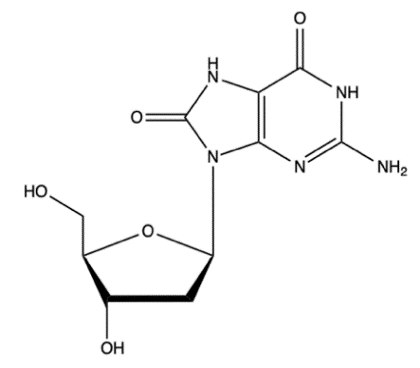 | ROS  Endogenous sources, alcohol, tobacco | [17]Cooke et al. 2018:  Human urine  [15]Ma et al. 2016:  Human retina  [18]Chou et al. 2010:  Human colon, kidney, liver, lung, pancreas, and spleen tissue | [17]Cooke et al. 2018:  LC-MS/MS  [15]Ma et al. 2016:  LC-MS/MS  [18]Chou et al. 2010:  LC-MS/MS | |  |
| 24 | 4-OH-8-oxo-dG  4-Hydroxy-8-oxo-dG  C_10_H_13_N_5_O_6_  299.086585 | 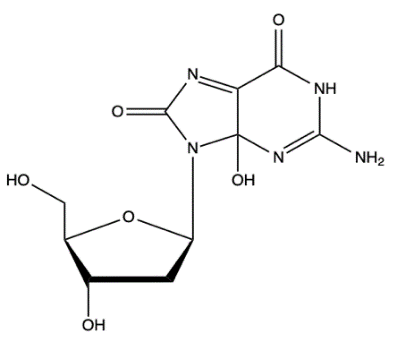 | ROS  Endogenous sources | [19]Ravanat, J. and Cadet, J. 1995:  Calf thymus DNA  [11]Carra` et al. 2019:  Mouse lung tissue | [19]Ravanat, J. and Cadet, J. 1995:  LC-UV/MS and NMR  [11]Carra` et al. 2019:  2D-LC-HRMS/MS  (tentatively identified) | |  |
| 25 | 5-OH-dC  5-Hydroxy-dC  C_9_H_13_N_3_O_5_  243.085522 | 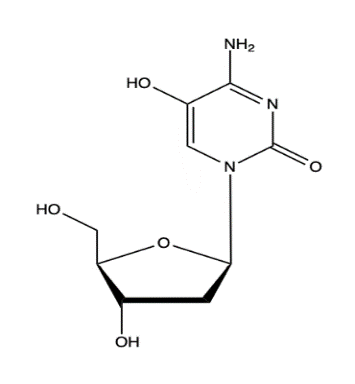 | ROS, LPO  Endogenous sources, alcohol, pollution | [20]Douki et al. 1996:  Calf thymus DNA | [20]Douki et al. 1996:  LC/GC-MS | |  |
| 26 | 5-OH-dU  5-Hydroxy-dU  C_9_H_12_N_2_O_6_  244.069538 | 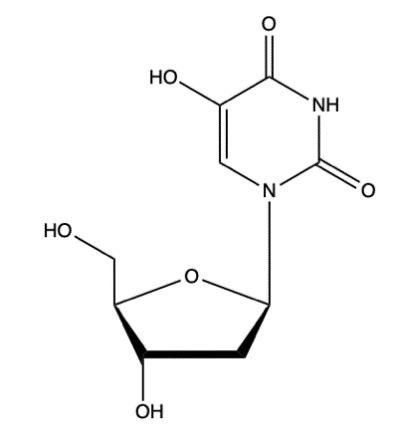 | ROS, LPO  Endogenous sources, UV irradiation, tobacco | [16]Frelon et al. 2000:  Calf Thymus DNA | [16]Frelon et al. 2000:  LC-MS/MS | |  |
| 27 | 5,6-Di-H-dT  5,6-Dihydro-dT  C_10_H_16_N_2_O_5_  244.105923 | 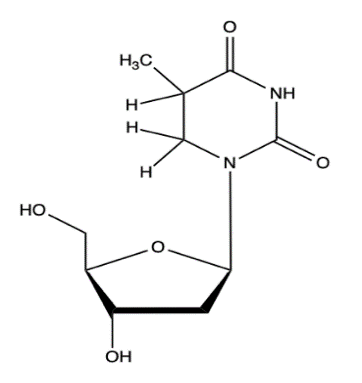 | ROS  Endogenous sources, alcohol, pollution | [8]Dizdaroglu et al. 1993:  Calf thymus DNA | [8]Dizdaroglu et al. 1993:  GC-MS | |  |
| 28 | 5,6-Di-H-dU  5,6-Dihydro-dU  C_9_H_14_N_2_O_5_  230.090273 | 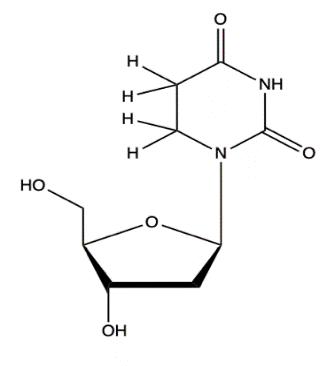 | ROS  Endogenous sources | [8]Dizdaroglu et al. 1993:  Calf thymus DNA | [8]Dizdaroglu et al. 1993:  GC-MS | |  |
| 29 | 5-OH-5,6-H-dC  5-Hydroxy-5,6-Dihydro-dC  C_9_H_15_N_3_O_5_  245.101172 |  | ROS  Endogenous sources | [11]Carra` et al. 2019:  Mouse lung tissue | [11]Carra` et al. 2019:  2D-LC-HRMS/MS  (tentatively identified) | |  |
| 30 | 5-OH-5,6-H-dU  5-Hydroxy-5,6-Dihydro-dU  C_9_H_14_N_2_O_6_  246.085188 | 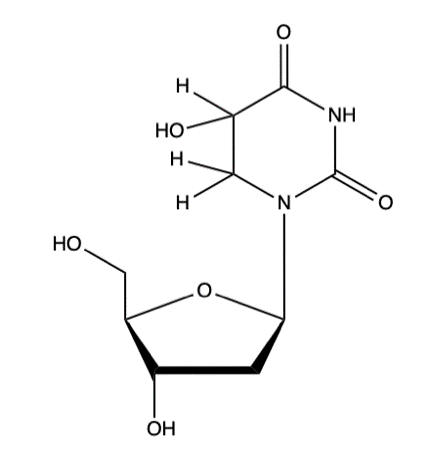 | ROS  Endogenous sources | [11]Carra` et al. 2019:  Mouse lung tissue | [11]Carra` et al. 2019:  2D-LC-HRMS/MS  (tentatively identified) | |  |
| 31 | Glycol-dT  C_10_H_16_N_2_O_7_  276.095753  5,6-Di-OH-5,6-Di-H-dT | 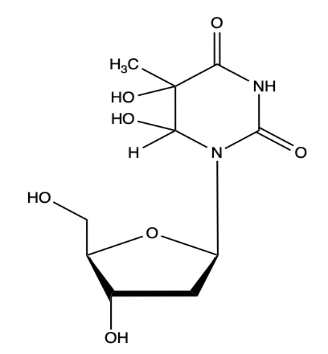 | ROS, LPO  Endogenous sources, alcohol, pollution | [8]Dizdaroglu et al. 1993:  Calf thymus DNA | [8]Dizdaroglu et al. 1993:  GC-MS | |  |
| 32 | Glycol-dU  C_9_H_14_N_2_O_7_  262.080103  5,6-Di-OH-5,6-Di-H-dU | 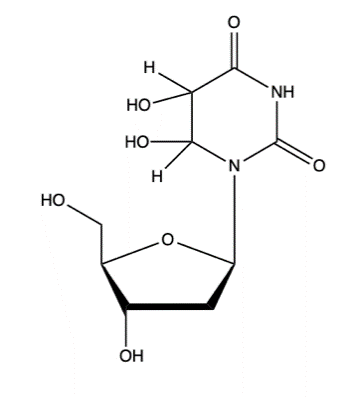 | ROS, LPO  Endogenous sources | [8]Dizdaroglu et al. 1993:  Calf thymus DNA | [8]Dizdaroglu et al. 1993:  GC-MS | |  |
| 33 | 3-Me-dA  3-Methyl-dA  C_11_H_16_N_5_O_3_^+^  266.125315 | 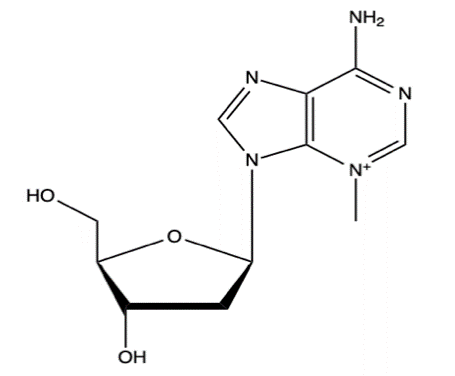 | NOC  Alkylation    Endogenous sources, red and processed meat, tobacco, pollution | [21] Tang et al. 2019:  Human kidney and liver cells  [17]Cooke et al. 2018:  Human urine  [22]Chang et al 2018:  Mouse liver tissue | [21]Tang et al. 2019:  LC-MS/MS  [17]Cooke et al. 2018:  LC-MS/MS  [22]Chang et al 2018:  LC-MS/MS | | |
| 34 | N^6^-Me-dA  N^6^-Methyl-dA  C_11_H_15_N_5_O_3_  265.117490 | 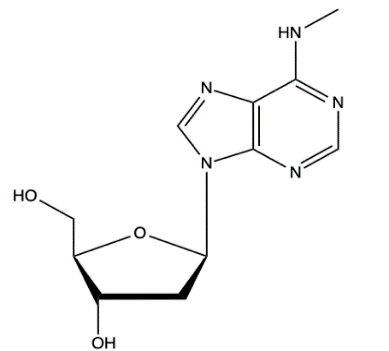 | NOC  Alkylation  Endogenous sources, red and processed meat, tobacco, pollution | [17]Cooke et al. 2018:  Human urine  [23] Lu et al. 2012:  Human cervical adenoma cells (Hela S3)  [11]Carra` et al. 2019:  Mouse lung tissue | [17]Cooke et al. 2018:  LC-MS/MS  [23] Lu et al. 2012:  LC-MS/MS  [11]Carra` et al. 2019:  2D-LC-HRMS/MS | | |
| 35 | 1-Me-dA  1-Methyl-dA  C_11_H_16_N_5_O_3_^+^  266.125315 | 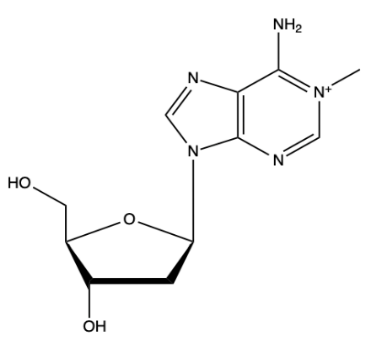 | NOC  Alkylation  Endogenous sources, red and processed meat, tobacco, pollution | [21] Tang et al. 2019:  Human kidney and liver cells  [1] Hsu et al. 2009:  Human urine  [22]Chang et al 2018:  Mouse liver tissue | [21] Tang et al. 2019:  LC-MS/MS  [1] Hsu et al. 2009:  LC-MS/MS  [22]Chang et al 2018:  LC-MS/MS | | |
| 36 | 7-Me-dA  7-Methyl-dA  C_11_H_16_N_5_O_3_^+^  266.125315 | 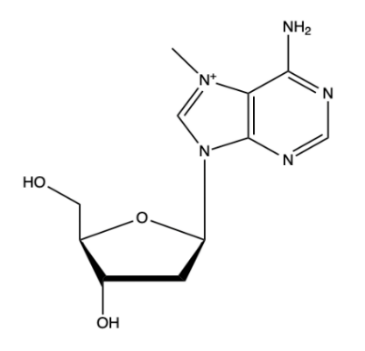 | NOC  Alkylation  Endogenous sources, red and processed meat, tobacco, pollution | [21] Tang et al. 2019:  Human kidney and liver cells | [21] Tang et al. 2019:  LC-MS/MS | | |
| 37 | 2-Me-dA  2-Methyl-dA  C_11_H_15_N_5_O_3_  265.117490 |  | NOC  Alkylation  Endogenous sources, red and processed meat, tobacco, pollution | [23] Lu et al. 2012:  Human cervical adenoma cells (Hela S3) | [23] Lu et al. 2012:  LC-MS/MS | | |
| 38 | O^6^-Me-dG  O^6^-Methyl-dG  C_11_H_15_N_5_O_4_  281.112405 | 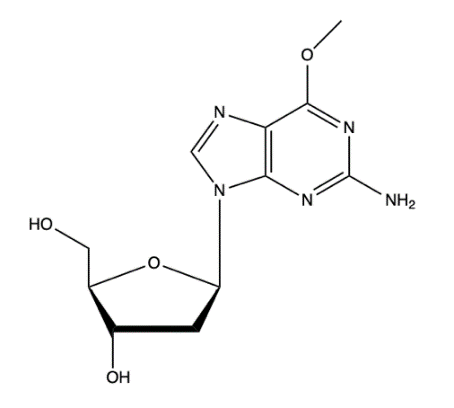 | NOC  Alkylation    Endogenous sources,  red and processed meat, tobacco, pollution | [24] Leu et al. 2015:  Human rectal tissue  [11]Carra` et al. 2019:  Mouse lung tissue  [22]Chang et al 2018:  Mouse liver tissue | [24] Leu et al. 2015:  Immunohistochemical method  [11]Carra` et al. 2019:  2D-LC-HRMS/MS  [22]Chang et al 2018:  LC-MS/MS | |  |
| 39 | 7-Me-dG  7-Methyl-dG  C_11_H_16_N_5_O_4_^+^  282.120230 | 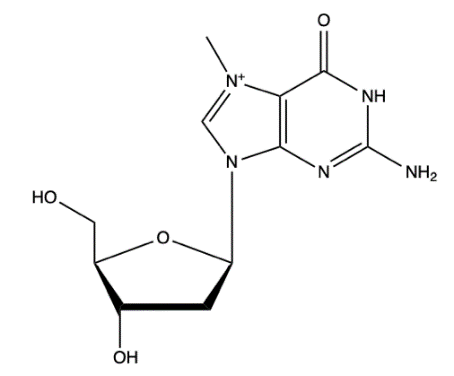 | NOC  Alkylation    Endogenous sources,  red and processed meat, tobacco, pollution | [17]Cooke et al. 2018:  Human urine  [21] Tang et al. 2019:  Human kidney and liver cells  [22]Chang et al 2018:  Mouse liver tissue | [17]Cooke et al. 2018:  LC-MS/MS  [21] Tang et al. 2019:  LC-MS/MS  [22]Chang et al 2018:  LC-MS/MS | |  |
| 40 | 1-Me-dG  1-Methyl-dG  C_11_H_15_N_5_O_4_  281.112405 | 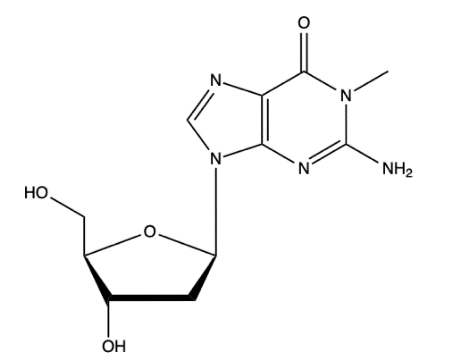 | NOC  Alkylation  Endogenous sources, red and processed meat, tobacco, pollution | [21] Tang et al. 2019:  Human kidney and liver cells | [21] Tang et al. 2019:  LC-MS/MS | |  |
| 41 | 5-Me-dC  5-Methyl-dC  C_10_H_15_N_3_O_4_  241.106257 | 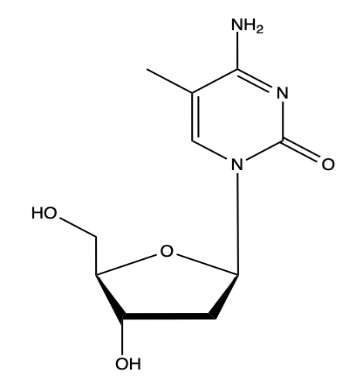 | Alkylation  Endogenous sources | [25]Liu et al. 2019:  Human liver tissue  [21] Tang et al. 2019:  Human kidney and liver cells | [25]Liu et al. 2019:  LC-MS/MS  [21] Tang et al. 2019:  LC-MS/MS | | |
| 42 | 3-Me-dC  3-Methyl-dC  C_10_H_15_N_3_O_4_  241.106257 |  | Alkylation | [1] Hsu et al. 2009:  Human urine  [22]Chang et al 2018: Mouse liver tissue | [1] Hsu et al. 2009:  LC-MS/MS  [22]Chang et al 2018:  LC-MS/MS | | |
| 43 | O^4^-Me-dT  O^4^-Methyl-dT  C_11_H_16_N_2_O_5_  256.105922 | 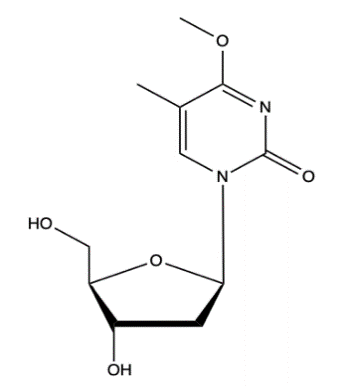 | Alkylation  Endogenous sources, tobacco, pollution | [11]Carra` et al. 2019:  Mouse lung tissue | [11]Carra` et al. 2019:  2D-LC-HRMS/MS  (tentatively identified) | | |
| 44 | 3-Et-dA  3-Ethyl-dA  C_12_H_18_N_5_O_3_^+^  280.140965 | 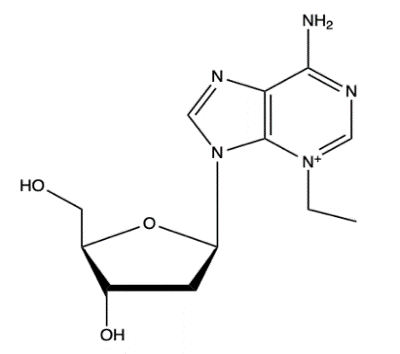 | NOC  Alkylation  Tobacco | [26] Chen, H. J. C. and Liu, Y. F. 2013:  Human blood | [26] Chen, H. J. C. and Liu, Y. F. 2013:  capLC-MS/MS | | |
| 45 | N^6^-Et-dA  N^6^-Ethyl-dA  C_12_H_17_N_5_O_2_  279.13314 |  | Acetaldehyde  Alkylation  Alcohol | [27]Guidolin 2021: Human oral cells (human study) | [27]Guidolin 2021:  nanoLC-HRMS/MS | |  |
| 46 | N^2^-Et-dG  N^2^-Ethyl-dG  C_12_H_17_N_5_O_4_  295.128055 | 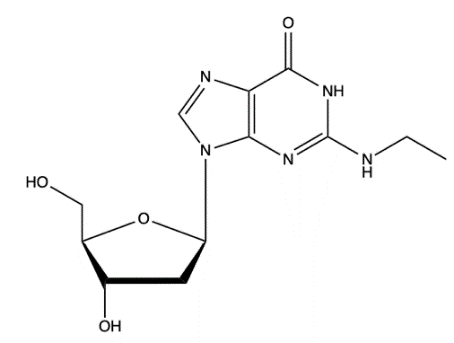 | Acetaldehyde  Alkylation  Alcohol, tobacco | [28] Matsuda et al. 2006:  Human blood  [27]Guidolin 2021:  Human oral cells (human study)  [11]Carra` et al. 2019:  Mouse lung tissue | [28] Matsuda et al. 2006:  LC-MS/MS  [27]Guidolin 2021:  nanoLC-HRMS/MS  [11]Carra` et al. 2019:  2D-LC-HRMS/MS | |  |
| 47 | 7-Et-dG  7-Ethyl-dG  C_12_H_18_N_5_O_4_  295.128055 |  | Alkylation | [29]Balbo et  al. 2011:  Human blood  [22]Chang et al 2018:  Mouse liver tissue | [29]Balbo et al. 2011:  nanoLC-HRMS/MS  [22]Chang et al 2018: LC-MS/MS | | |
| 48 | O^6^-Et-dG  O^6^-Ethyl-dG  C_12_H_17_N_5_O_4_  295.128055 |  | Alkylation | [22]Chang et al 2018:  Mouse liver tissue | [22]Chang et al 2018:  LC-MS/MS | | |
| 49 | N^2^,N^2^-DiMe-dG  N^2^,N^2^-Dimethyl-dG  C_12_H_17_N_5_O_4_  295.128055 | 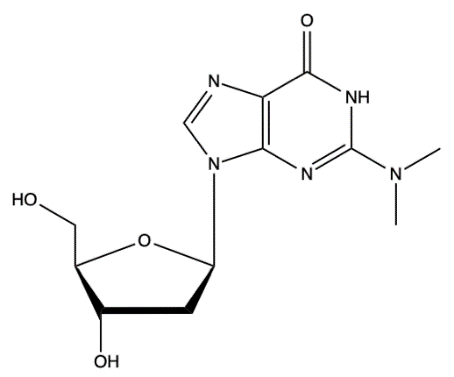 | Alkylation  Endogenous sources, tobacco, pollution | [1] Hsu et al. 2009:  Human urine | [1] Hsu et al. 2009:  LC-MS/MS | | |
| 50 | N^4^-Et-dC  N^4^-Ethyl-dC  C_11_H_17_N_3_O_4_  255.121907 |  | Acetaldehyde  Alkylation  Alcohol | [27]Guidolin 2021:  Human oral cells (human study) | [27]Guidolin 2021:  nanoLC-HRMS/MS | |  |
| 51 | O^4^-Et-dT  O^4^-Ethyl-dT  C_12_H_18_N_2_O_5_  270.121573 | 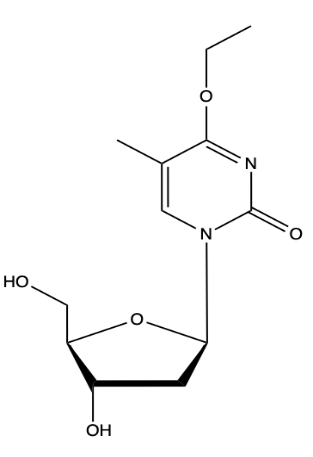 | NOC  Alkylation  Red and processed meat, alcohol, tobacco, pollution | [30] Huh et al. 1989:  Human liver tissue  [31] Chen et al. 2012:  Human blood  [32]Chen et al. 2014:  Human saliva | [30] Huh et al. 1989:  LC and immunochemical method  [31] Chen et al. 2012:  capLC-MS/MS  [32]Chen et al. 2014:  nanoLC-HRMS/MS | |  |
| 52 | O^2^-Et-dT  O^2^-Ethyl-dT  C_12_H_18_N_2_O_5_  270.121573 | 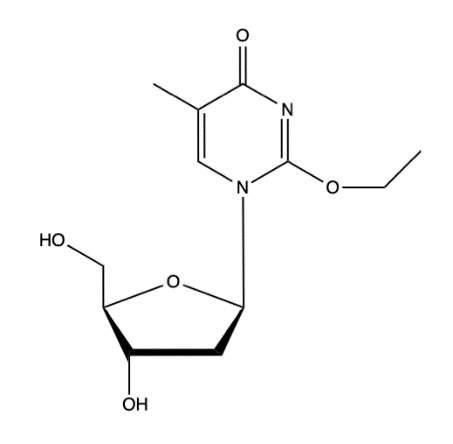 | NOC  Alkylation  Red and processed meat, alcohol, tobacco, pollution | [31]Chen et al. 2012:  Human blood  [32]Chen et al. 2014:  Human saliva | [31]Chen et al. 2012:  capLC-MS/MS  [32]Chen et al. 2014:  nanoLC-HRMS/MS | | |
| 53 | 3-Et-dT  3-Ethyl-dT  C_12_H_18_N_2_O_5_  270.121573 |  | NOC  Alkylation  Red and processed meat, alcohol, tobacco, pollution | [31] Chen et al. 2012:  Human blood  [32]Chen et al. 2014:  Human saliva | [31]Chen et al. 2012:  capLC-MS/MS  [32]Chen et al. 2014:  nanoLC-HRMS/MS | | |
| 54 | 7-Pr-dG  7-Propyl-dG  C_13_H_20_N_5_O_4_^+^  310.151530 | 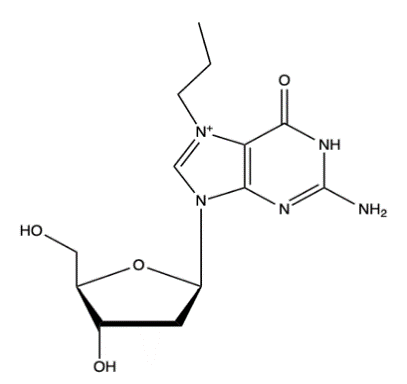 | ROS  Alkylation  Pollution | [22]Chang et al 2018:  Mouse liver tissue | [22]Chang et al 2018:  LC-MS/MS | | |
| 55 | O^6^-But-dG  O^6^-Butyl-dG  C_14_H_21_N_5_O_4_  323.159355 | 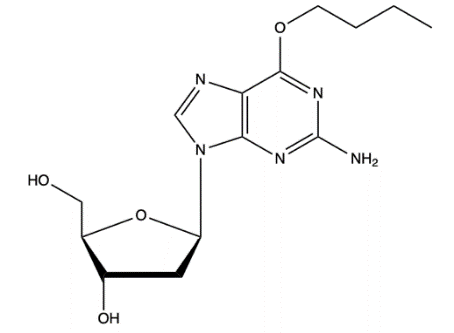 | NOC  Alkylation  Red and processed meat, tobacco, pollution | [33] Bonfanti et al. 1990:  Rat liver tissue | [33] Bonfanti et al. 1990:  Immunochemical method, GC-MS and NMR | | |
| 56 | N^6^-OH-Me-dA  N^6^-Hydroxy-methyl-dA  C_11_H_15_N_5_O_4_  281.112405 | 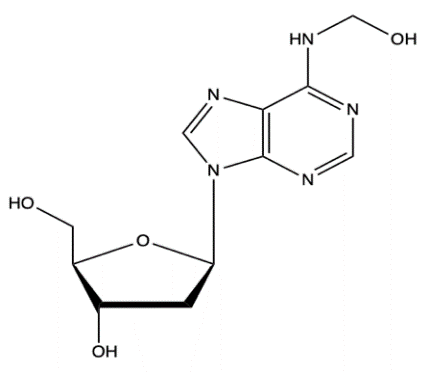 | Formaldehyde, LPO  Alkylation,  Endogenous sources, some foods, tobacco, pollution | Lu et al. 2012:[23]  Human cervical adenoma cells (Hela S3)  [34]Chang et al. 2021:  Calf Thymus DNA | Lu et al. 2012: [23]  LC-MS/MS  [34]Chang et al. 2021: LC-HRMS/MS (tentatively identified) | | |
| 57 | N^2^-OH-Me-dG  N^2^-Hydroxy-methyl-dG  C_11_H_15_N_5_O_5_  297.107320 | 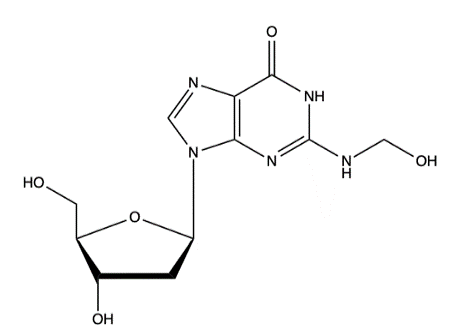 | Formaldehyde, LPO  Alkylation  Endogenous sources, some foods, tobacco, pollution | Lu et al. 2012:[23]  Human cervical adenoma cells (Hela S3)  [34]Chang et al. 2021:  Calf Thymus DNA | Lu et al. 2012:[23]  LC-MS/MS  [34]Chang et al. 2021: LC-HRMS/MS (tentatively identified) | | |
| 58 | 5-OH-Me-dC  5-Hydroxy-methyl-dC  C_10_H_15_N_3_O_5_  257.101172 | 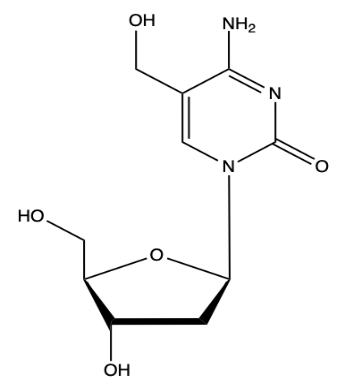 | LPO  Alkylation  Endogenous sources | [25]Liu et al. 2019:  Human liver tissue  Tang et al. 2019:[21]  Human kidney and liver cells | [25]Liu et al. 2019:  LC-MS/MS  Tang et al. 2019:[21]  LC-MS/MS | | |
| 59 | N^4^-OH-Me-dC  N^4^-Hydroxy-methyl-dC  C_10_H_15_N_3_O_5_  257.101172 |  | Formaldehyde, LPO  Endogenous sources | [34]Chang et al. 2021:  Calf Thymus DNA | [34]Chang et al. 2021:  LC-HRMS/MS (tentatively identified) | |  |
| 60 | 5-OH-Me-dU  5-Hydroxy-methyl-dU  C_10_H_14_N_2_O_6_  258.085188 | 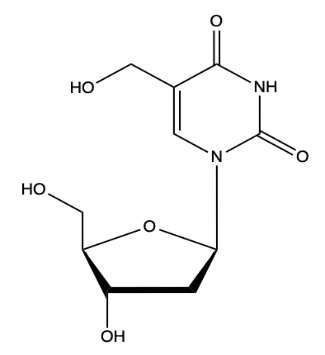 | ROS, LPO  Endogenous sources, UV irradiation, tobacco | [16]Frelon et al. 2000:  Calf Thymus DNA  [11]Carra` et al. 2019:  Mouse lung tissue | [16]Frelon et al. 2000:  LC-MS/MS  [11]Carra` et al. 2019:  2D-LC-HRMS/MS  (tentatively identified) | |  |
| 61 | 1-(2-OH-Et)-dA  1-(2-Hydroxy-ethyl)-dA  C_12_H_18_N_5_O_4_^+^  296.135880 | 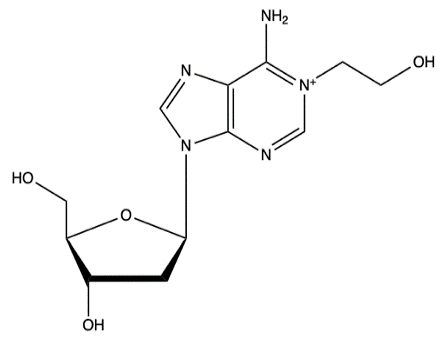 | LPO  Alkylation  Endogenous sources, tobacco, red and processed meat, pollution | [35]Li et al. 1992:  Calf thymus DNA | [35]Li et al. 1992:  LC-MS | | |
| 62 | 3-(2-OH-Et)-dA  3-(2-Hydroxy-ethyl)-dA  C_12_H_18_N_5_O_4_^+^  296.135880 | 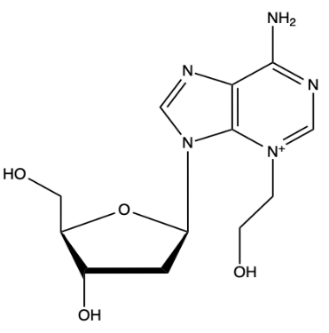 | LPO  Alkylation  Endogenous sources, tobacco, red and processed meat, pollution | [35]Li et al. 1992:  Calf thymus DNA | [35]Li et al. 1992:  LC-MS | | |
| 63 | N^6^-(2-OH-Et)-dA  N^6^-(2-Hydroxy-ethyl)-dA  C_12_H_17_N_5_O_4_  295.128055 | 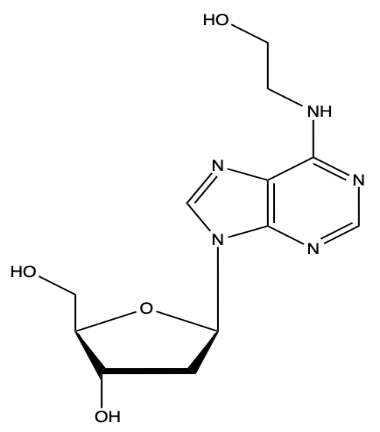 | LPO  Alkylation  Endogenous sources, tobacco, red and processed meat, pollution | [35]Li et al. 1992:  Calf thymus DNA | [35]Li et al. 1992:  LC-MS | | |
| 64 | 8-(1-OH-Et)-dG  8-(1-Hydroxy-ethyl)-dG  C_12_H_17_N_5_O_5_  311.122970 | 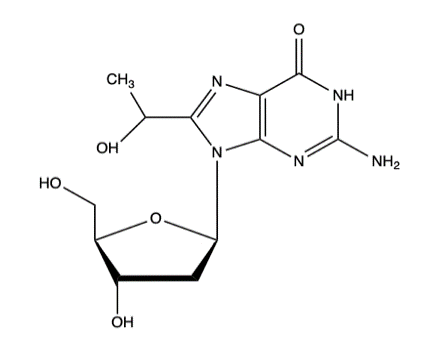 | LPO  Alkylation  Endogenous sources, alcohol | [36]Nakao et al. 2002:  Rat liver tissue | [36]Nakao et al. 2002:  LC-MS/MS | | |
| 65 | 7-(2-OH-Et)-dG  7-(2-Hydroxy-ethyl)-dG  C_12_H_18_ N_5_O_5_^+^  312.130795 | 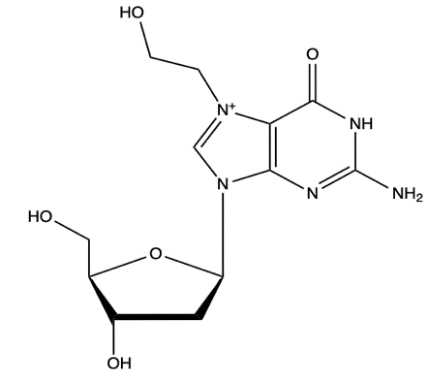 | LPO  Alkylation  Endogenous sources, tobacco, red and processed meat, pollution | [37]Wu et al. 1999:  Human blood | [37]Wu et al. 1999:  GC-MS | | |
| 66 | 3-(2-OH-Et)-dC  3-(2-Hydroxy-ethyl)-dC  C_11_H_18_N_3_O_5_^+^  272.124647 | 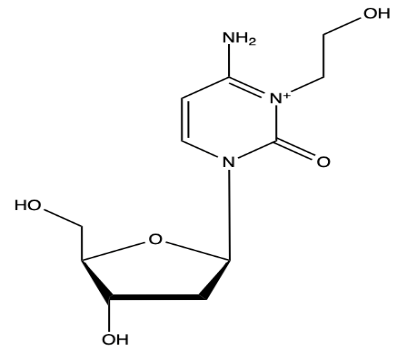 | LPO  Alkylation  Endogenous sources, tobacco, red and processed meat, pollution | [35]Li et al. 1992:  Calf thymus DNA | [35]Li et al. 1992:  LC-MS | | |
| 67 | 3-(2-OH-Et)-dT  3-(2-Hydroxy-ethyl)-dT  C_12_H_18_N_2_O_6_  286.116488 | 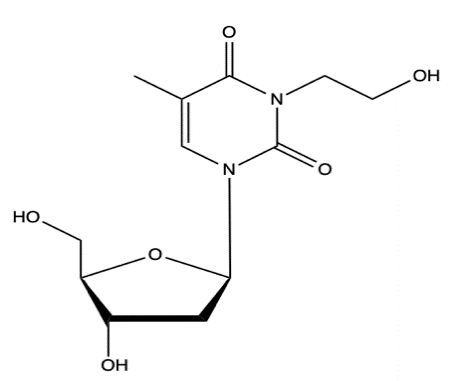 | LPO  Alkylation  Endogenous sources, tobacco, red and processed meat, pollution | [35]Li et al. 1992:  Calf thymus DNA | [35]Li et al. 1992:  LC-MS | | |
| 68 | 3-(2-OH-Et)-dU  3-(2-Hydroxy-ethyl)-dU  C_11_H_16_N_2_O_6_  272.100838 | 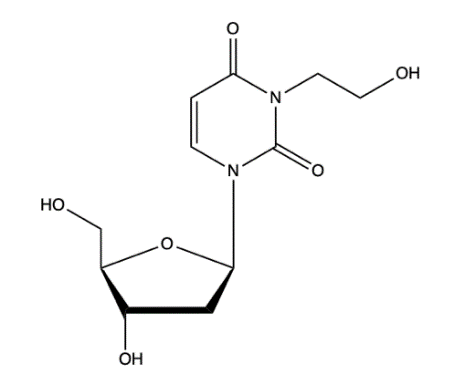 | LPO  Alkylation  Endogenous sources, tobacco, red and processed meat, pollution | [35]Li et al. 1992:  Calf thymus DNA | [35]Li et al. 1992:  LC-MS | | |
| 69 | N^6^-OHMe-N^6^-Me-dA  N^6^-Hydroxymethyl- N^6^-methyl-dA  C_12_H_17_N_5_O_4_  295.128055 |  | Formaldehyde, LPO  Alkylation  Endogenous sources | [34]Chang et al. 2021:  Calf Thymus DNA | [34]Chang et al. 2021:  LC-HRMS/MS (tentatively identified) | | |
| 70 | N^4^-OHMe-5-Me-dC  N^4^-Hydroxymethyl-5-Methyl-dC  C_11_H_17_N_3_O_5_  271.11682 |  | Formaldehyde, LPO  Alkylation  Endogenous sources | [34]Chang et al. 2021:  Calf Thymus DNA | [34]Chang et al. 2021:  LC-HRMS/MS (tentatively identified) | | |
| 71 | N^6^ -EtOMe-dA  N^6^-ethoxymethyl-dA  C_13_H_19_N_5_O_4_  309.14370 |  | Formaldehyde, LPO  Alkylation  Endogenous sources | [34]Chang et al. 2021:  Calf Thymus DNA | [34]Chang et al. 2021:  LC-HRMS/MS (tentatively identified) | |  |
| 72 | N^6^-OHMe-N^6^-EtOMeOH-dA  N^6^-hydroxymethyl-N^6^ -ethoxymethanol-dA  C_14_H_21_N_5_O_6_  355.14918 |  | Formaldehyde, LPO  Alkylation  Endogenous sources | [34]Chang et al. 2021:  Calf Thymus DNA | [34]Chang et al. 2021:  LC-HRMS/MS (tentatively identified) | |  |
| 73 | N^6^-(4-OH-But)-dA  N^6^-(4-Hydroxy-butyl)-dA  C_14_H_21_N_5_O_4_  323.159355 | 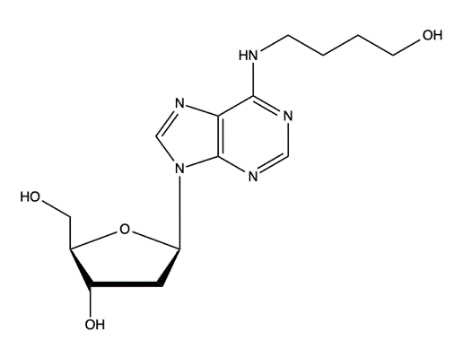 | N-nitroso-pyrrolidine  NOC  Tobacco, red and processed meat | [38]Wang et al. 2007:  Calf thymus DNA | [38]Wang et al. 2007:  LC-MS and NMR | |  |
| 74 | N^2^-(3-OH-But)-dG  N^2^-(3-Hydroxy-butyl)-dG  C_14_H_21_N_5_O_5_  339.154270 | 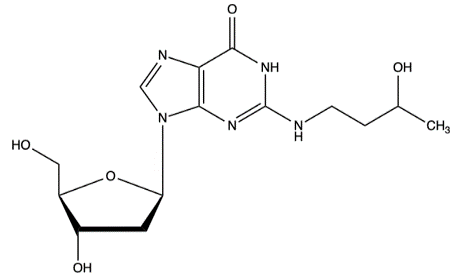 | Acetaldehyde, Crotonaldehyde, ROS, LPO  Endogenous sources,  alcohol, red and processed meat, tobacco, pollution | [39]Wang et al. 2001:  Calf thymus DNA | [39]Wang et al. 2001:  LC-MS/MS, UV and NMR | |  |
| 75 | N^4^-(4-OH-But)-dC  N^4^-(4-Hydroxy-butyl)-dC  C_13_H_21_N_3_O_5_  299.148122 | 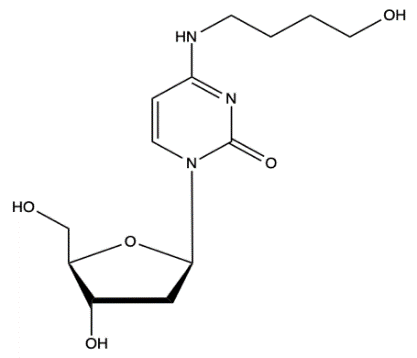 | N-nitroso-pyrrolidine  NOC  Tobacco, red and processed meat | [38]Wang et al. 2007:  Calf thymus DNA | [38]Wang et al. 2007:  LC-MS and NMR | |  |
| 76 | O^4^-(4-OH-But)-dT  O^4^-(4-Hydroxy-butyl)-dT  C_14_H_22_N_2_O_6_  314.147788 | 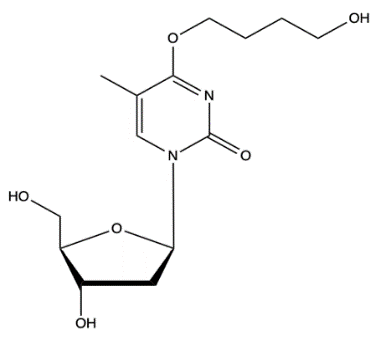 | N-nitroso-pyrrolidine  NOC  Tobacco, red and processed meat | [38]Wang et al. 2007:  Calf thymus DNA | [38]Wang et al. 2007:  LC-MS and NMR | |  |
| 77 | O^2^-(4-OH-But)-dT  O^2^-(4-Hydroxy-butyl)-dT  C_14_H_22_N_2_O_6_  314.147788 |  | N-nitroso-pyrrolidine  NOC  Tobacco, red and processed meat | [38]Wang et al. 2007:  Calf thymus DNA | [38]Wang et al. 2007:  LC-MS and NMR | | |
| 78 | 3-(4-OH-But)-dT  3-(4-Hydroxy-butyl)-dT  C_14_H_22_N_2_O_6_  314.147788 |  | N-nitroso-pyrrolidine  NOC  Tobacco, red and processed meat | [38]Wang et al. 2007:  Calf thymus DNA | [38]Wang et al. 2007:  LC-MS and NMR | | |
| 79 | 5-Formyl-dC  C_10_H_13_N_3_O_5_  255.08552 |  | ROS, Alkylation  Endogenous sources | [40]Ito et al: Mouse embryionic stem cells and tissues | [40]Ito et al: TLC-MS | | |
| 80 | 5-Formyl-dU  C_10_H_12_N_2_O_6_  256.06954 |  | ROS, Alkylation  Endogenous sources, UV irradiation | [16]Frelon et al. 2000:  Calf Thymus DNA | [16]Frelon et al. 2000:  LC-MS/MS | | |
| 81 | 5-Carboxy-dC  C_10_H_13_N_3_O_6_  271.080436 |  | Alkylation  Endogenous sources | [40]Ito et al: Mouse embryionic stem cells and tissues | [40]Ito et al: TLC-MS | | |
| 82 | N^6^-Carboxy-Me-dA  N^6^-Carboxymethyl-dA  C_12_H_15_N_5_O_5_  309.107320 | 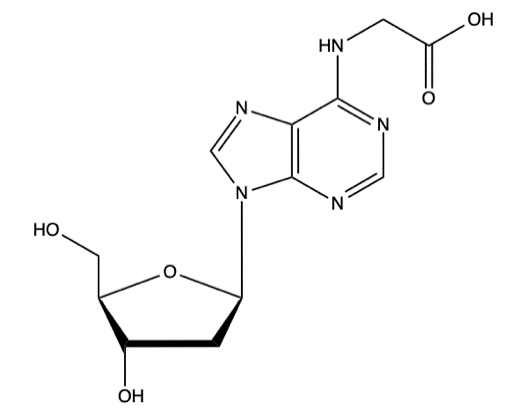 | NOC  Alkylation  Endogenous sources, red and processed meat, tobacco, pollution | [41] Wang, J. and Wang, Y. 2010:  Calf thymus DNA | [41] Wang, J. and Wang, Y. 2010:  LC-MS/MS | | |
| 83 | O^6^-Carboxy-Me-dG  O^6^-Carboxymethyl-dG  C_12_H_15_N_5_O_6_  325.102235 | 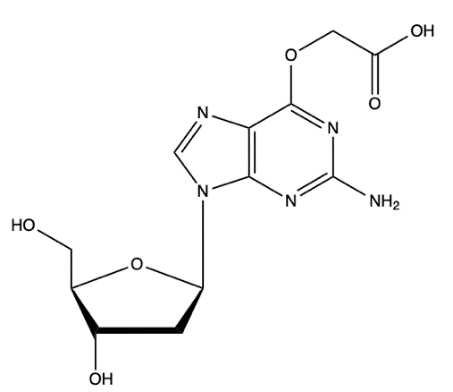 | NOC  Alkylation  Endogenous sources, red and processed meat, tobacco, pollution | [42] Lewin et al. 2006:  Human fecal samples | [42] Lewin et al. 2006: Immunohistochemical method | |  |
| 84 | N^2^-Carboxy-Me-dG  N^2^-Carboxymethyl-dG  C_12_H_15_N_5_O_6_  325.102235 | 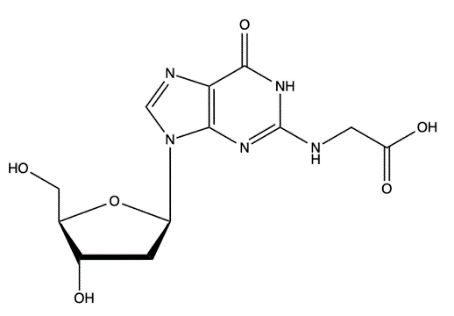 | ROS  Alkylation  Endogenous sources, tobacco and pollution | [43] Wang et al. 2010:  Human kidney cells  [11]Carra` et al. 2019:  Mouse lung tissue | [43] Wang et al. 2010:  LC-MS/MS  [11]Carra` et al. 2019:  2D-LC-HRMS/MS  (tentatively identified) | | |
| 85 | N^4^-Carboxy-Me-dC  N^4^-Carboxymethyl-dC  C_11_H_15_N_3_O_6_  285.096087 | 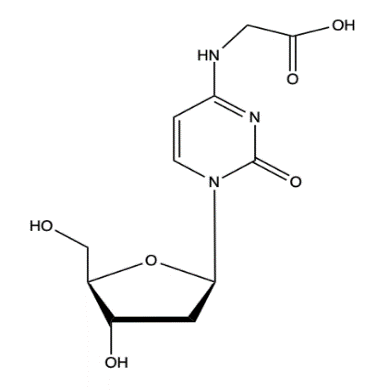 | NOC  Alkylation  Endogenous sources red and processed meat, tobacco, pollution | [41] Wang, J. and Wang, Y. 2010:  Calf thymus DNA | [41] Wang, J. and Wang, Y. 2010:  LC-MS/MS | | |
| 86 | O^4^-Carboxy-Me-dT  O^4^-Carboxymethyl-dT  C_12_H_16_N_2_O_7_  300.095753 | 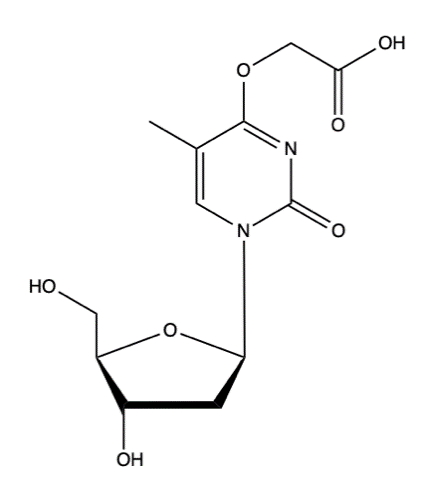 | NOC  Alkylation  Endogenous sources red and processed meat, tobacco, pollution | [44] Wang, J. and Wang, Y. 2009:  Calf thymus DNA | [44] Wang, J. and Wang, Y. 2009:  LC-MS/MS and NMR | | |
| 87 | 3-Carboxy-Me-dT  3-Carboxymethyl-dT  C_12_H_16_N_2_O_7_  300.095753 | 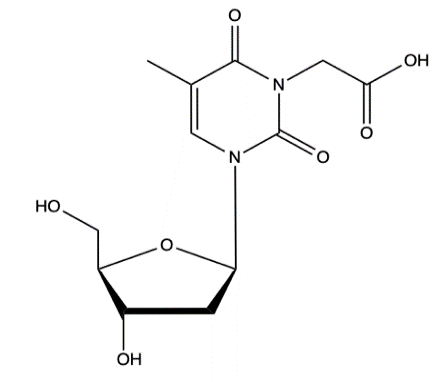 | NOC  Alkylation  Endogenous sources red and processed meat, tobacco, pollution | [44] Wang, J. and Wang, Y. 2009:  Calf thymus DNA | [44] Wang, J. and Wang, Y. 2009:  LC-MS/MS and NMR | | |
| 88 | N^2^-(1-Carboxy-Et)-dG  N^2^-(1-Carboxyethyl)-dG  C_13_H_17_N_5_O_6_  339.117885 |  | Glyoxal,  LPO, ROS  Alkylation  Endogenous sources, heated foods, pollution, and others | [45]Cheng et al 2020:  Human blood  [46]Yuan et al. 2008:  Human melanoma cells | [45]Cheng et al 2020:  nanoLC-HRMS/MS  [46]Yuan et al. 2008:  LC-HRMS/MS | |  |
| 89 | 7-(2-Carboxy-Et)-dG  7-(2-Carboxy-ethyl)-dG  C_13_H_18_N_5_O_6_^+^  340.125710 |  | NOC  Alkylation  Endogenous sources, red and processed meat, tobacco and pollution | [47] Cheng G. et al. 2010:  Human liver tissue  [45]Cheng et al 2020:  Human blood  [11]Carra` et al. 2019:  Mouse lung tissue | [47] Cheng G. et al. 2010:  LC-MS/MS  [45]Cheng et al 2020:  nanoLC-HRMS/MS  [11]Carra` et al. 2019:  2D-LC-HRMS/MS  (tentatively identified) | | |
| 90 | N^6^-(2-Carboxy-2-OH-Et)-dA  N^6^-(2-Carboxy-2-hydroxy-ethyl)-dA  C_13_H_17_N_5_O_6_  339.117885 | 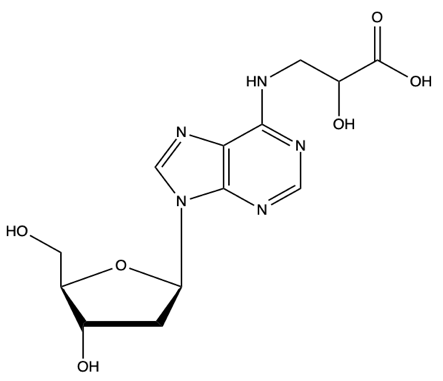 | Acrylamide  Alkylation  High temperature cooked starchy food | [48]Costa et al. 2003:  Salmon testis DNA | [48]Gamboa da Costa et al. 2003:  LC-MS/MS | |  |
| 91 | 1-(2-Carboxy-2-OH-Et)-dA  1-(2-Carboxy-2-hydroxy-ethyl)-dA  C_13_H_18_N_5_O_6_^+^  340.125710 | 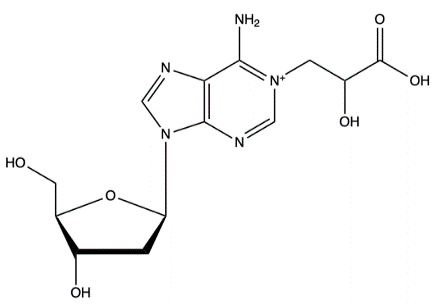 | Acrylamide  Alkylation  High temperature cooked starchy food | [48]Gamboa da Costa et al. 2003:  Salmon testis DNA | [48]Gamboa da Costa et al. 2003:  LC-MS/MS | |  |
| 92 | N^2^-(1-Carboxy-3-OH-Pr)-dG  N^2^-(1-Carboxy-3-hydroxy-propyl)-dG  C_14_H_19_N_5_O_7_  369.128450 | 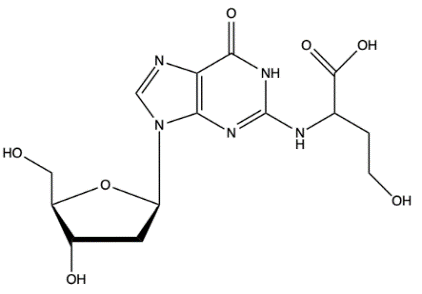 | ROS  Alkylation  Endogenous sources, fruit and vegetables | [49]Larisch et al. 1997: Incubation with dG (*In vitro*) | [49]Larisch et al. 1997:  LC-UV and NMR | | |
| 93 | 3-HMHP-dA  3-Hydroxymethyl-2-hydroxy-propane-dA  C_14_H_20_N_5_O_5_^+^  338.146444 | 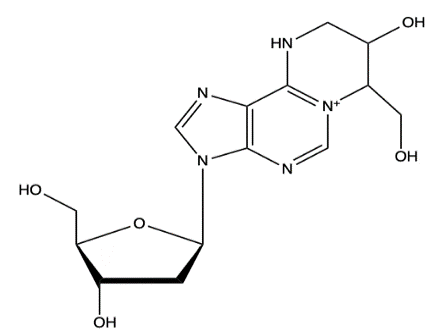 | 1,3-Butadiene, ROS  Alkylation  Endogenous sources,  tobacco, pollution | [50]Goggin et al. 2010:  Mouse liver tissue | [50]Goggin et al. 2010:  LC-MS/MS | | |
| 94 | 1-HMHP-dA  1-Hydroxymethyl-2-hydroxy-propane-dA  C_14_H_20_N_5_O_5_^+^  338.146444 | 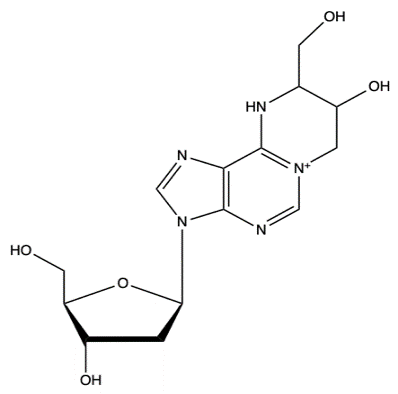 | 1,3-Butadiene, ROS  Alkylation  Endogenous sources,  tobacco, pollution | [50]Goggin et al. 2010:  Mouse liver tissue | [50]Goggin et al. 2010:  LC-MS/MS | | |
| 95 | 3HM-3HP-dG  3-Hydroxymethyl-3-hydroxypropane-dG  C_14_H_19_N_5_O_6_  353.133535  HMVK-dG | 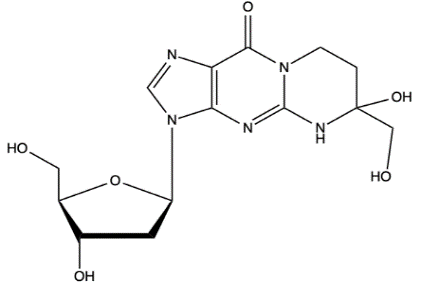 | 1,3-Butadiene, ROS  Alkylation  Endogenous sources,  tobacco, pollution | [51]Powley et al. 2003:  Calf thymus DNA | [51]Powley et al. 2003:  LC-MS/MS | | |
| 96 | 1,N^6^-ε-dA  1,N^6^-etheno-dA  C_12_H_13_N_5_O_3_  275.101840 | 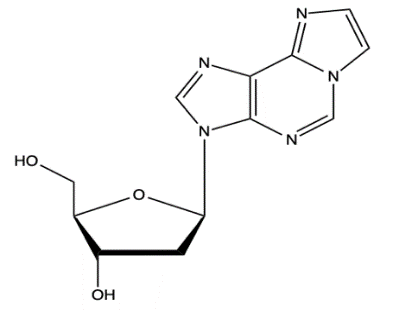 | ROS, LPO  Endogenous sources, alcohol, tobacco | [52]Paiano et al. 2020:  Human oral cells (human study)  [53]Bin et al. 2015: Human urine  [18]Chou et al. 2010:  Human colon, kidney, liver, lung, pancreas, and spleen tissue | [52]Paiano et al. 2020:  nanoLC-HRMS/MS  [53]Bin et al. 2015: LC-MS/MS  [18]Chou et al. 2010:  LC-MS/MS | |  |
| 97 | 1,N^2^-ε-dG  1,N^2^-etheno-dG  C_12_H_13_N_5_O_4_  291.096755 | 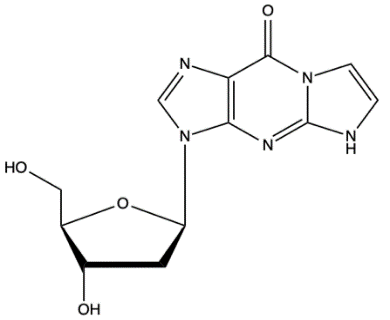 | ROS, LPO  Endogenous sources, alcohol | [54]Gonzalez-Reche et al. 2002:  Human urine  [2]Pang et al. 2007: Mouse spleen, liver and kidney tissue | [54]Gonzalez-Reche et al. 2002:  GC/LC-MS/MS  [2]Pang et al. 2007:  LC-MS/MS | |  |
| 98 | 3,N^2^-ε-dG  3,N^2^-etheno-dG  C_12_H_13_N_5_O_4_  291.096755 | 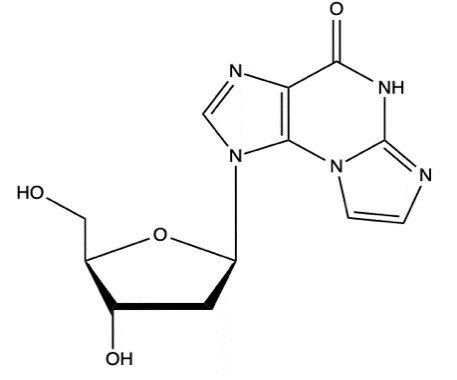 | ROS, LPO  Endogenous sources, alcohol | [54]Gonzalez-Reche et al. 2002:  Human urine | [54]Gonzalez-Reche et al. 2002:  GC/LC-MS/MS | |  |
| 99 | 3,N^4^-ε-dC  3,N^4^-Etheno-dC  C_11_H_13_N_3_O_4_  251.090607 |  | RNS, ROS, LPO  Endogenous sources, alcohol, tobacco | [52]Paiano et al. 2020:  Human oral cells (human study)  [53]Bin et al. 2015: Human urine | [52]Paiano et al. 2020:  nanoLC-HRMS/MS  [53]Bin et al. 2015: LC-MS/MS | |  |
| 100 | Oxo-ε-dG  Oxo-etheno-dG  C_12_H_13_N_5_O_5_  307.091670 | 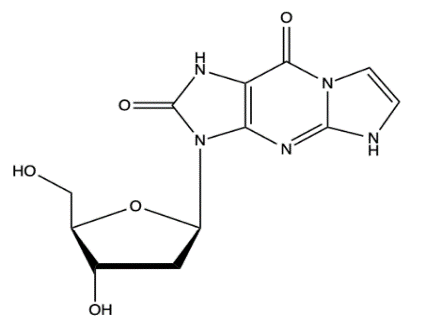 | ROS, LPO  Endogenous sources, alcohol | [11]Carra` et al. 2019:  Mouse lung tissue | [11]Carra` et al. 2019:  2D-LC-HRMS/MS  (tentatively identified) | |  |
| 101 | OHE-dA  Oxohexenal-dA  C_16_H_19_N_5_O_4_  345.143705  Butanone-ε-dA |  | Oxohexenal, LPO  Endogenous sources | [18]Chou et al. 2010: Human colon, liver and lung tissue | [18]Chou et al. 2010:  LC-MS/MS | |  |
| 102 | OHE-dG  Oxohexenal-dG  C_16_H_19_N_5_O_5_  361.138620  Butanone-ε-dG | 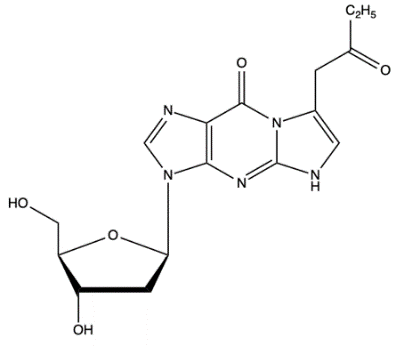 | Oxohexenal, LPO  Endogenous sources | [18]Chou et al. 2010: Human colon, liver and lung tissue | [18]Chou et al. 2010:  LC-MS/MS | |  |
| 103 | OHE-dC  Oxohexenal-dC  C_15_H_19_N_3_O_5_  321.132472  Butanone-ε-dC | 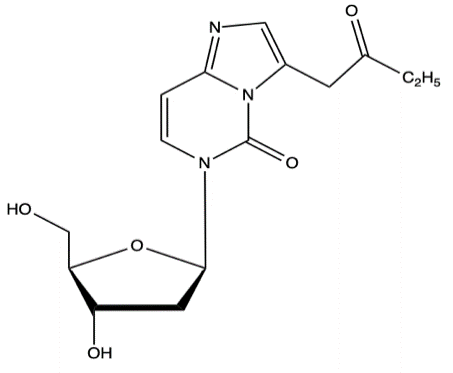 | Oxohexenal, LPO  Endogenous sources | [18]Chou et al. 2010:  Human colon, kidney, liver, lung, pancreas, and spleen tissue | [18]Chou et al. 2010:  LC-MS/MS | |  |
| 104 | OHE-5-Me-dC Oxohexenal-5-methyl-dC  C_16_H_21_N_3_O_5_  335.148122  Butanone-5-Me-ε-dC | 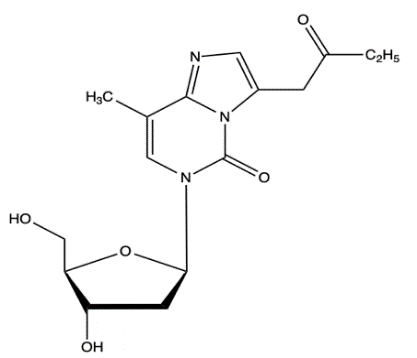 | Oxohexenal, LPO  Endogenous sources | [18]Chou et al. 2010:  Human colon, liver and lung tissue | [18]Chou et al. 2010:  LC-MS/MS | |  |
| 105 | ONE-dA  Oxononenal-dA  C_19_H_25_N_5_O_4_  387.190655  Heptanone-ε-dA |  | Oxononenal, LPO  Endogenous sources | [18]Chou et al. 2010:  Human colon, heart, kidney, liver, lung, pancreas, small intestine, and spleen tissue  [55]Guo et al 2018:  Human bladder tissue  [56]Guo et al 2017:  Human prostate tissue | [18]Chou et al. 2010:  LC-MS/MS  [55]Guo et al 2018:  nanoLC-HRMS/MS  [56]Guo et al 2017:  nanoLC-HRMS/MS (tentatively identified) | |  |
| 106 | ONE-dG  Oxononenal-dG  C_19_H_25_N_5_O_5_  403.185570  Heptanone-ε-dG |  | Oxononenal, LPO  Endogenous sources | [18]Chou et al. 2010:  Human colon, heart, kidney, liver, lung, pancreas, small intestine, and spleen tissue  [55]Guo et al 2018:  Human bladder tissue  [56]Guo et al 2017:  Human prostate tissue | [18]Chou et al. 2010:  LC-MS/MS  [55]Guo et al 2018:  nanoLC-HRMS/MS  [56]Guo et al 2017:  nanoLC-HRMS/MS (tentatively identified) | |  |
| 107 | ONE-dC  Oxononenal-dC  C_18_H_25_N_3_O_5_  363.179422  Heptanone-ε-dC |  | Oxononenal, LPO  Endogenous sources | [18]Chou et al. 2010:  Human colon, heart, kidney, liver, lung, pancreas, small intestine, and spleen tissue  [55]Guo et al 2018:  Human bladder tissue | [18]Chou et al. 2010:  LC-MS/MS  [55]Guo et al 2018:  nanoLC-HRMS/MS (tentatively identified) | |  |
| 108 | ONE-II-dA  Oxononenal-II-dA  C_19_H_27_N_5_O_5_  405.201220  Heptanone-OH-ε-dA |  | Oxononenal, LPO  Endogenous sources, pollution | [57]Lee et al. 2000:  Calf thymus DNA | [57]Lee et al. 2000:  LC-MS/MS and NMR | |  |
| 109 | ONE-II-dC  Oxononenal-II-dC  C_18_H_27_N_3_O_6_  381.189987  Heptanone-OH-ε-dC |  | Oxononenal, LPO  Endogenous sources, pollution | [58]Pollack et al. 2003:  Incubation with dC (*In vitro*) | [58]Pollack et al. 2003:  LC-MS/MS and NMR | |  |
| 110 | Oxo-heptanone-ε-dG  Oxo-heptanone-etheno-dG  C_19_H_25_N_5_O_6_  419.180485 |  | Aldehyde, LPO  Endogenous sources, pollution | [59]Knutson et al. 2009:  Oxidation of heptanone-etheno-dG (*In vitro*) | [59]Knutson et al. 2009:  LC-MS/MS | |  |
| 111 | DDE-I-dA  Decadienal-I-dA  C_19_H_27_N_5_O_5_  405.201220  Di-OH-heptane-ε-dA |  | Decadienal, LPO  Endogenous sources, pollution | [60]Carvalho et al. 1998:  Calf thymus DNA | [60]Carvalho et al. 1998:  LC-MS/MS and NMR | |  |
| 112 | DDE-II-dA  Decadienal-II-dA  C_20_H_29_N_5_O_6_  435.211785  Tri-OH-octane-ε-dA |  | Decadienal, LPO  Endogenous sources, pollution | [60]Carvalho et al. 1998:  Calf thymus DNA | [60]Carvalho et al. 1998:  LC-MS/MS and NMR | |  |
| 113 | DDE-I-dG  Decadienal-I-dG  C_19_H_27_N_5_O_6_  421.196135  Di-OH-heptane-ε-dG |  | Decadienal, LPO  Endogenous sources | [56]Guo et al 2017:  Human prostate tissue  [55]Guo et al 2018:  Human bladder tissue | [56]Guo et al 2017:  nanoLC-HRMS/MS (tentatively identified)  [55]Guo et al 2018:  nanoLC-HRMS/MS (tentatively identified) | |  |
| 114 | DDE-III-dG  Decadienal-III-dG  C_20_H_27_N_5_O_6_  433.196135  OH-octanone-ε-dG |  | Decadienal, LPO, ROS  Endogenous sources, pollution | [61]Loureiro et al. 2000:  Calf thymus DNA | [61]Loureiro et al. 2000:  LC-MS/MS and NMR | |  |
| 115 | DDE-I-dC  Decadienal-I-dC  C_18_H_24_N_3_O_6_  381.189987  Di-OH-heptane-ε-dC |  | Decadienal, LPO  Endogenous sources | [56]Guo et al 2017:  Human prostate tissue  [55]Guo et al 2018:  Human bladder tissue | [56]Guo et al 2017:  nanoLC-HRMS/MS (tentatively identified)  [55]Guo et al 2018:  nanoLC-HRMS/MS (tentatively identified) | |  |
| 116 | Hexenal-dG  C_16_H_23_N_5_O_5_  365.169920  1, N^2^-(propyl-P)-dG |  | Hexenal, LPO  Endogenous sources,  alcohol, fruit and vegetables | [62]Schuler, D. and Eder, E. 1999:  Rat esophagus, forestomach and liver tissue | [62]Schuler, D. and Eder, E. 1999:  ^32^P-postlabeling | |  |
| 117 | HNE-I-dG  Hydroxynonenal-I-dG  C_19_H_29_N_5_O_6_  423.211785  OH-hexyl-P-dG |  | Hydroxynonenal, LPO  Endogenous sources, alcohol | [11]Carra` et al. 2019:  Mouse lung tissue  [55]Guo et al 2018:  Human bladder tissue | [11]Carra` et al. 2019:  2D-LC-HRMS/MS  [55]Guo et al 2018:  nanoLC-HRMS/MS | |  |
| 118 | Acr-1I-dA  Acrolein-1I-dA  C_13_H_17_N_5_O_4_  307.128055  1,N^6^-(OH-P)-dA |  | Acrolein, LPO  Endogenous sources, pollution, tobacco, burnt fatty foods | [63]Pawlowicz et al. 2006:  Calf thymus DNA | [63]Pawlowicz et al. 2006:  LC-MS/MS | | |
| 119 | Acr-1II-dA  Acrolein-1II-dA  C_13_H_17_N_5_O_4_  307.128055  N^6^-(3-oxo-propyl)-dA |  | Acrolein, LPO  Endogenous sources, pollution, tobacco, burnt fatty foods | [63]Pawlowicz et al.2006:  Calf thymus DNA | [63]Pawlowicz et al. 2006:  LC-MS/MS | | |
| 120 | Acr-2I-dA  Acrolein-2I-dA  C_16_H_22_N_5_O_5_^+^  364.162095  Bis-OH-P-dA |  | Acrolein, LPO  Endogenous sources, pollution, tobacco, burnt fatty foods | [63]Pawlowicz et al. 2006:  Calf thymus DNA | [63]Pawlowicz et al. 2006: LC-MS/MS | | |
| 121 | Acr-1I-dG  Acrolein-1I-dG  C_13_H_17_N_5_O_5_  323.122970  1,N^2^-γ-(OH-P)-dG |  | Acrolein, LPO  Endogenous sources, pollution, tobacco, burnt fatty foods | [18]Chou et al. 2010:  Human colon, kidney, liver, lung, pancreas, and spleen tissue  [64]Yang et al 2019:  Human lung tissue  [52]Paiano et al. 2020:  Human oral cells (human study) | [18]Chou et al. 2010:  LC-MS/MS  [64]Yang et al 2019:  nanoLC-HRMS/MS  [52]Paiano et al. 2020:  nanoLC-HRMS/MS | | |
| 122 | Acr-1II-dG  Acrolein-1II-dG  C_13_H_17_N_5_O_5_  323.122970  1,N2-α-(OH-P)-dG |  | Acrolein, LPO  Endogenous sources, pollution, tobacco, burnt fatty foods | [64]Yang et al 2019:  Human lung tissue  [52]Paiano et al. 2020:  Human oral cells (Human study) | [64]Yang et al 2019:  nanoLC-HRMS/MS  [52]Paiano et al. 2020:  nanoLC-HRMS/MS | | |
| 123 | Acr-2II-dA  Acrolein-2II-dA  C_16_H_19_N_5_O_4_  345.143705 |  | Acrolein, LPO  Endogenous sources, pollution, tobacco, burnt fatty foods | [63]Pawlowicz et al. 2006: Calf thymus DNA  [11]Carra` et al. 2019:  Mouse lung tissue | [63]Pawlowicz et al. 2006: LC-MS/MS  [11]Carra` et al. 2019:  2D-LC-HRMS/MS  (tentatively identified) | | |
| 124 | Acr-1I-dC  Acrolein-1I-dC  C_12_H_18_N_3_O_5_^+^  284.12410  3,N^4^-(OH-P)-dC |  | Acrolein, LPO  Endogenous sources, pollution, tobacco, burnt fatty foods | [65]Pawlowicz et al. 2007: Calf thymus DNA | [65]Pawlowicz et al. 2007:  LC-MS/MS | | |
| 125 | Acr-2I-dC  Acrolein-2I-dC  C_15_H_22_N_3_O_6_^+^  340.150861  Bis-OH-P-dC |  | Acrolein, LPO  Endogenous sources, pollution, tobacco, burnt fatty foods | [65]Pawlowicz et al. 2007: Calf thymus DNA | [65]Pawlowicz et al. 2007:  LC-MS/MS | | |
| 126 | Acr-1I-dT  Acrolein-1I-dT  C_13_H_18_N_2_O_6_  298.116488  N^3^-(3-oxo-propyl)-dT |  | Acrolein, LPO  Endogenous sources, pollution, tobacco, burnt fatty foods | [66]Pawlowicz, A. J. and Kronberg, L. 2008:  Calf thymus DNA | [66]Pawlowicz, A. J. and Kronberg, L. 2008:  LC-MS | | |
| 127 | Acr-2I-dT  Acrolein-2I-dT  C_16_H_24_N_2_O_8_  372.153268 |  | Acrolein, LPO  Endogenous sources, pollution, tobacco, burnt fatty foods | [66]Pawlowicz, A. J. and Kronberg, L. 2008:  Incubation with dT (*in vitro*) | [66]Pawlowicz, A. J. and Kronberg, L. 2008:  LC-MS | | |
| 128 | Acr-2II-dT  Acrolein-2II-dT  C_16_H_22_N_2_O_7_  354.142703 |  | Acrolein, LPO  Endogenous sources, pollution, tobacco, burnt fatty foods | [66]Pawlowicz, A. J. and Kronberg, L. 2008:  Incubation with dT (*in vitro*) | [66]Pawlowicz, A. J. and Kronberg, L. 2008:  LC-MS | | |
| 129 | Acr-2III-dT  Acrolein-2III-dT  C_16_H_22_N_2_O_7_  354.142703 |  | Acrolein, LPO  Endogenous sources, pollution, tobacco, burnt fatty foods | [66]Pawlowicz, A. J. and Kronberg, L. 2008:  Incubation with dT (*in vitro*) | [66]Pawlowicz, A. J. and Kronberg, L. 2008:  LC-MS | | |
| 130 | Acr-2IV-dT  Acrolein-2IV-dT  C_16_H_20_N_2_O_6_  336.132138 |  | Acrolein, LPO  Endogenous sources, pollution, tobacco, burnt fatty foods | [66]Pawlowicz, A. J. and Kronberg, L. 2008:  Incubation with dT (*in vitro*)  [11]Carra` et al. 2019:  Mouse lung tissue | [66]Pawlowicz, A. J. and Kronberg, L. 2008:  LC-MS  [11]Carra` et al. 2019:  2D-LC-HRMS/MS  (tentatively identified) | | |
| 131 | Cro-1I-dG  Crotonaldehyde-1I-dG  C_14_H_19_N_5_O_5_  337.138620  1,N^2^-(Me-OH-P)-dG |  | Crotonaldehyde, LPO  Endogenous sources, pollution, tobacco, alcohol, burnt fatty foods | [28]Matsuda et al. 2006:  Human blood  [18]Chou et al. 2010:  Human colon, kidney, liver, lung, pancreas, and spleen tissue  [67]Nath et al 1998:  Gingival tissue | [28] Matsuda et al. 2006:  LC-MS/MS  [18]Chou et al. 2010:  LC-MS/MS  [67]Nath et al 1998:  LC-MS | |  |
| 132 | Cro-1II-dG  Crotonaldehyde-1II-  dG  C_14_H_20_N_5_O_5_^+^  338.146444  8,N^7^-(Me-OH-P)-dG |  | Crotonaldehyde, LPO  Endogenous sources, pollution, tobacco, alcohol, burnt fatty foods | [68]Eder, E. and Hoffman, C. 1992:  Incubation with dG (*in vitro*) | [68]Eder, E. and Hoffman, C. 1992:  LC-MS and NMR | | |
| 133 | Cro-2I-dG  Crotonaldehyde-2I-dG  C_18_H_26_N_5_O_6_^+^  408.188310  Bis-Me-OH-P-dG |  | Crotonaldehyde, LPO  Endogenous sources, pollution, tobacco, alcohol, burnt fatty foods | [68]Eder, E. and Hoffman, C. 1992:  Incubation with dG (*in vitro*) | [68]Eder, E. and Hoffman, C. 1992:  LC-MS and NMR | | |
| 134 | Cro-2II-dG  Crotonaldehyde-2II-dG  C_18_H_27_N_5_O_7_  425.191050  N^2^-Paraldol-dG |  | Crotonaldehyde, ROS, LPO  Endogenous sources, tobacco, alcohol | [69]Wang et al. 2000a:  Calf thymus DNA  [11]Carra` et al. 2019:  Mouse lung tissue | [69]Wang et al. 2000a:  LC-MS/MS and NMR  [11]Carra` et al. 2019:  2D-LC-HRMS/MS  (tentatively identified) | | |
| 135 | M1-dA  Malondialdehyde-1-dA  C_13_H_15_N_5_O_4_  305.112405  N^6^-Oxopropenyl-dA |  | Malondialdehyde, LPO, ROS  Endogenous sources | [11]Carra` et al. 2019:  Mouse lung tissue | [11]Carra` et al. 2019:  2D-LC-HRMS/MS  (tentatively identified) | |  |
| 136 | M3-dA  Malondialdehyde-3-dA  C_19_H_19_N_5_O_6_  413.133535 |  | Malondialdehyde, LPO, ROS  Endogenous sources | [70]Stone et al. 1990a: Incubation with dA (*In vitro*) | [70]Stone et al. 1990a:  LC-UV and NMR | | |
| 137 | M1-dG  Malondialdehyde-1-dG  C_13_H_13_N_5_O_4_  303.096755 |  | Malondialdehyde, LPO, ROS  Endogenous sources, tobacco,  red and processed meat | [71]Saieva et al. 2016:  Human blood  [72]Ma et al. 2014:  Human blood  [73]Leuratti et al 2002:  Human colon tissue | [71]Saieva et al. 2016:  ^32^P-postlabelling  [72]Ma et al. 2014:  nanoLC-HRMS/MS  [73]Leuratti et al 2002:  LC-FLNS | |  |
| 138 | M2-dG  Malondialdehyde-2-dG  C_16_H_17_N_5_O_6_  375.117885 |  | Malondialdehyde, LPO, ROS  Endogenous sources,  red and processed meat | [11]Carra` et al. 2019:  Mouse lung tissue | [11]Carra` et al. 2019:  2D-LC-HRMS/MS  (tentatively identified) | |  |
| 139 | M1-dC  Malondialdehyde-1-dC  C_12_H_15_N_3_O_5_  281.101172  N^4^-Oxopropenyl-dC |  | Malondialdehyde, LPO, ROS  Endogenous sources | [74]Stone et al. 1990b:  Incubation with dC (*In vitro*) | [74]Stone et al 1990b. LC-UV and NMR | | |
| 140 | M3-dC  Malondialdehyde-3-dC  C_18_H_19_N_3_O_7_  389.122302 |  | Malondialdehyde, LPO, ROS  Endogenous sources,  red and processed meat | [74]Stone et al. 1990b:  Incubation with dC (*In vitro*) | [74]Stone et al.1990b: LC-UV and NMR | | |
| 141 | N^2^-Ethylidene-dG  C_12_H_15_N_5_O_4_  293.112405 |  | Acetaldehyde, LPO  Alcohol | [75]Balbo et al. 2012:  Human blood  [76] Matsuda et al. 2007:  Mouse liver | [75]Balbo et al. 2012:  LC-MS/MS  [76] Matsuda et al. 2007:  LC-MS/MS | |  |
| 142 | N^2^-DiMe-dioxane-dG  N^2^-Dimethyldioxane-dG  C_16_H_23_N_5_O_6_  381.164835 |  | Acetaldehyde  Alcohol | [77]Wang et al. 2000b:  Calf thymus DNA | [77]Wang et al. 2000b:  LC-UV/MS and NMR | |  |
| 143 | M1AAI-dA  1Malonyl-acetaldehyde-dA  C_15_H_17_N_5_O_4_  331.128055 |  | Malondialdehyde, Acetaldehyde, LPO, ROS  Endogenous sources | [78]Pluskota-Karwatka et al. 2006:  Calf thymus DNA  [11]Carra` et al. 2019:  Mouse lung tissue | [78]Pluskota-Karwatka et al. 2006:  LC-MS  [11]Carra` et al. 2019:  2D-LC-HRMS/MS  (tentatively identified) | | |
| 144 | M2AAII-dA  Malonyl-2-acetaldehyde-II-dA  C_18_H_19_N_5_O_5_  385.138620 |  | Malondialdehyde, Acetaldehyde, LPO, ROS  Endogenous sources | [78]Pluskota-Karwatka et al. 2006:  Calf thymus DNA | [78]Pluskota-Karwatka et al. 2006:  LC-MS | | |
| 145 | M2AAI-dG  Malonyl-2-acetaldehyde-I-dG  C_18_H_19_N_5_O_6_  401.133535 |  | Malondialdehyde, Acetaldehyde, LPO, ROS  Endogenous sources | [78]Pluskota-Karwatka et al. 2006:  Calf thymus DNA | [78]Pluskota-Karwatka et al. 2006:  LC-MS | | |
| 146 | M2AAII-dG  Malonyl-2-acetaldehyde-II-dG  C_18_H_19_N_5_O_6_  401.133535 |  | Malondialdehyde, Acetaldehyde, LPO, ROS  Endogenous sources | [78]Pluskota-Karwatka et al. 2006:  Calf thymus DNA | [78]Pluskota-Karwatka et al. 2006:  LC-MS | | |
| 147 | M1AAI-dC  Malonyl-1-acetaldehyde-dC  C_14_H_17_N_3_O_5_  307.116822 |  | Malondialdehyde, Acetaldehyde, LPO, ROS  Endogenous sources | [79]Pluskota-Karwatka et al. 2002: Incubation with dC (*In vitro*) | [79]Pluskota-Karwatka et al. 2002:  LC-UV/MS | | |
| 148 | M2AAII-dC  Malonyl-2-acetaldehyde-II-dC  C_17_H_19_N_3_O_6_  361.127387 |  | Malondialdehyde, Acetaldehyde, LPO, ROS  Endogenous sources | [78]Pluskota-Karwatka et al. 2006:  Calf thymus DNA  [11]Carra` et al. 2019:  Mouse lung tissue | [78]Pluskota-Karwatka et al. 2006:  LC-MS  [11]Carra` et al. 2019:  2D-LC-HRMS/MS  (tentatively identified) | | |
| 149 | M1FAI-dC  Malonyl-1-formaldehyde-dC  C_13_H_15_N_3_O_5_  293.10117 |  | Malondialdehyde, Formaldehyde, LPO, ROS  Endogenous sources | [79]Pluskota-Karwatka et al. 2002: Incubation with dC (*In vitro*) | [79]Pluskota-Karwatka et al. 2002:  LC-UV/MS | | |
| 150 | 6-Oxo-M1-dG  6-Oxo-malondialdehyde-1-dG  C_13_H_13_N_5_O_5_  319.091670 |  | Malondialdehyde, LPO, ROS  Endogenous sources | [80]Otteneder et al. 2006:  Rat urine | [80]Otteneder et al. 2006:  LC-UV/MS | | |
| 151 | 2,6-diOxo-M1-dG  2,6-diOxo-malondialdehyde-1-dG  C_13_H_13_N_5_O_6_  335.086580 |  | Malondialdehyde, LPO ROS  Endogenous sources | [81]Knutson et al. 2007:  Rat liver cytosol and urine | [81]Knutson et al. 2007:  LC-MS/MS and NMR | |  |
| 152 | N^6^-OH-Ac-dA  N^6^-hydroxy-acetyl-dA  C_12_H_15_N_5_O_5_  309.107320 |  | Glyoxal, LPO, ROS  Endogenous sources, heated foods, pollution, and others | [82]Olsen et al. 2005:  Calf thymus DNA  [11]Carra` et al. 2019:  Mouse lung tissue | [82]Olsen et al. 2005:  LC-MS  [11]Carra` et al. 2019:  2D-LC-HRMS/MS  (tentatively identified) | |  |
| 153 | Glyoxal-dG  C_12_H_15_N_5_O_6_  325.102235 |  | Glyoxal, LPO, ROS  Endogenous sources, heated foods, pollution, and others | [82]Olsen et al. 2005:  Calf thymus DNA | [82]Olsen et al. 2005:  LC-MS | |  |
| 154 | 5-OH-Ac-dC  5-Hydroxy-acetyl-dC  C_11_H_15_N_3_O_6_  285.096087 |  | Glyoxal, LPO, ROS  Endogenous sources, heated foods, pollution, and others | [82]Olsen et al. 2005:  Incubatio with dC (*In vitro*) | [82]Olsen et al. 2005:  LC-MS | |  |
| 155 | 5-OH-Ac-dU  5-Hydroxy-acetyl-dU  C_11_H_14_N_2_O_7_  286.0801 |  | Glyoxal, LPO, ROS  Endogenous sources, heated foods, pollution, and others | [82]Olsen et al. 2005:  Incubatio with dC (*In vitro*) | [82]Olsen et al. 2005:  LC-MS and NMR | |  |
| 156 | Me-glyoxal-dG  Methyl-glyoxal-dG  C_13_H_17_N_5_O_6_  339.117885 |  | Me-Glyoxal, LPO, ROS  Endogenous sources, heated foods, pollution, and others | [83]Vaca et al. 1994:  Calf thymus DNA | [83]Vaca et al. 1994:  ^32^P-postlabelling | |  |
| 157 | 3-(2-Carbamoyl-2-OH-Et)-dA  3-(2-Carbamoyl-2-hydroxy-ethyl)-dA  C_13_H_19_N_6_O_5_^+^  339.141694 |  | Acrylamide    High temperature cooked starchy foods | [48]Gamboa da Costa et al. 2003:  Mouse liver, lung, and kidney tissue | [48]Gamboa da Costa et al. 2003:  LC-MS/MS | |  |
| 158 | 7-(2-Carbamoyl-2-OH-Et)-dG  7-(2-Carbamoyl-2-hydroxy-ethyl)-dG  C_13_H_19_N_6_O_6_^+^  355.136609 |  | Acrylamide  High temperature cooked starchy foods | [48]Gamboa da Costa et al. 2003:  Mouse liver, lung, and kidney tissue | [48]Gamboa da Costa et al. 2003:  LC-MS/MS | |  |
| 159 | cis-BDA-dA  cis-butene-dial-dA  C_14_H_17_N_5_O_5_  335.122970 |  | cis-butene-dial, Furan  Heated foods and others | [84]Byrns et al. 2006:  Bacterial and Calf Thymus DNA | [84]Byrns et al. 2006:  LC-MS/MS | |  |
| 160 | cis-BDA-dG  cis-butene-dial-dG  C_14_H_17_N_5_O_6_  351.117885 |  | cis-butene-dial,  Heated foods and others | [84]Byrns et al. 2006:  Incubation with dG (In vitro) | [84]Byrns et al. 2006:  LC-MS/MS | |  |
| 161 | cis-BDA-dC  cis-butene-dial-dC  C_13_H_17_N_3_O_6_  311.111737 |  | cis-butene-dial,  Heated foods and others | [84]Byrns et al. 2006:  Bacterial and calf thymus DNA | [84]Byrns et al. 2006:  LC-MS/MS | |  |
| 162 | N^6^-Furan-Me-dA  N^6^-Furan-methyl-dA  C_15_H_17_N_5_O_4_  331.128055 |  | Furfuryl alcohol  Furan  Heated foods and others | [85]Monien et al. 2011:  Mouse liver, lung and kidney tissue | [85]Monien et al. 2011:  LC-MS/MS | |  |
| 163 | N^2^-Furan-Me-dG  N^2^-Furan-methyl-dG  C_15_H_17_N_5_O_5_  347.122970 |  | Furfuryl alcohol  Furan  Heated foods and others | [85]Monien et al. 2011:  Mouse liver, lung and kidney tissue | [85]Monien et al. 2011:  LC-MS/MS | |  |
| 164 | N^6^-Formyl-furan-Me-dA  N^6^-Formyl-furan-methyl-dA  C_16_H_17_N_5_O_5_  359.122970  N^6^-((formylfuran-5-yl)methyl)-dA |  | Hydroxymethyl-furfural  Furan  Heated foods and others | [86]Monien et al. 2012:  Hamster lung ﬁbroblast V79 cells | [86]Monien et al. 2012:  LC -MS/MS | |  |
| 165 | N^2^-Formyl-furan-Me-dG  N^2^-Formyl-furan-methyl-dG  C_16_H_17_N_5_O_6_  375.117885  N^2^-((formylfuran-5-yl)methyl)-dG |  | Hydroxymethyl-furfural  Furan  Heated foods and others | [86]Monien et al. 2012:  Hamster lung ﬁbroblast V79 cells | [86]Monien et al. 2012:  LC -MS/MS | |  |
| 166 | 7-AFB1-dG  7-AflatoxinB1-dG  C_27_ H_26_N_5_O_11_^+^  596.162885 |  | Mycotoxin  Cereals, nuts, dried fruit, spices. | [87]Yu et al. 1996:  Human urine  [88] Woo et al. 2011:  Mouse liver tissue | [87]Yu et al. 1996:  LC-FLNS  [88] Woo et al. 2011:  LC-MS/MS | |  |
| 167 | AFB1-FAPY-dG  AflatoxinB1-diaminohydroxyformamidopyrimidine  C_27_H_27_N_5_O_12_  613.165625 |  | Mycotoxin  Cereals, nuts, dried fruit, spices. | [88] Woo et al. 2011:  Mouse liver tissue | [88] Woo et al. 2011:  LC-MS/MS | |  |
| 168 | 8-OTA-dG  8-OchratoxinA-dG  C_30_H_30_N_6_O_10_  634.202344 |  | Mycotoxin  Cereals, nuts, dried fruit, spices and others | [89] Faucet et al. 2004:  Pig and rat kidney tissue | [89] Faucet et al. 2004:  LC-MS | |  |
| 169 | 8-Oꞌ-OTA-dG  8-Oꞌ-OchratoxinA-dG  C_30_H_30_N_6_O_10_  634.202344 |  | Mycotoxin  Cereals, nuts, dried fruit, spices, and others | [89] Faucet et al. 2004:  Pig and rat kidney tissue | [89] Faucet et al. 2004:  LC-MS | |  |
| 170 | 7-OH-sterigmatocystin-dG  7-Hydroxysterigmatocystin-dG  C_28_H_26_N_5_O_11_^+^  608.162885 |  | Mycotoxin  Cereals, nuts, dried fruit, spices | [90] Essigmann et al. 1979:  Calf Thymus DNA | [90] Essigmann et al. 1979: LC-NMR | |  |
| 171 | N^6^-DHP-I-dA  DehydropyrrolizidineI-dA  C_18_H_22_N_6_O_4_  386.170254  N^6^-OH-dehydrosupinidine-II-dA |  | DHP, Pyrrolizidine alkaloids  Honey, cereals, herbs and spices, and others | [91] Zhao et al. 2012:  Calf thymus DNA | [91] Zhao et al. 2012:  LC-HRMS/MS | |  |
| 172 | N^6^-DHP-II-dA  DehydropyrrolizidineII-dA  C_18_H_22_N_6_O_4_  386.170254  N^6^-OH-dehydrosupinidine-II-dA |  | DHP, Pyrrolizidine alkaloids  Honey, cereals, herbs and spices, and others | [91] Zhao et al. 2012:  Calf thymus DNA | [91] Zhao et al. 2012:  LC-HRMS/MS | |  |
| 173 | N^2^-DHP-I-dG  DehydropyrrolizidineI-dG  C_18_H_22_N_6_O_5_  402.165169  N^2^-OH-dehydrosupinidineI-dG |  | DHP, Pyrrolizidine alkaloids  Honey, cereals, herbs and spices, and others | [91] Zhao et al. 2012:  Calf thymus DNA | [91] Zhao et al. 2012:  LC-HRMS/MS | |  |
| 174 | N^2^-DHP-II-dG  DehydropyrrolizidineII-dG  C_18_H_22_N_6_O_5_  402.165169  N^2^-OH-dehydrosupinidineII-dG |  | DHP, Pyrrolizidine alkaloids  Honey, cereals, herbs and spices, and others | [91] Zhao et al. 2012:  Calf thymus DNA | [91] Zhao et al. 2012:  LC-HRMS/MS | |  |
| 175 | N^2^-estragole-dG  C_20_H_23_N_5_O_5_  413.169920 |  | Estragole, Alkenylbenzene  Herbs and spices, flavours and fragrances | [92]Phillips et al. 1981:  Mouse liver tissue  [93]Wiseman et al. 1985:  Incubation with dG (*In vitro*) | [92]Phillips et al. 1981:  LC and NMR  [93]Wiseman et al. 1985:  LC and NMR | |  |
| 176 | N^6^-isoestragole-dA  C_20_H_23_N_5_O_4_  397.175005 |  | Isoestragole, Alkenylbenzene  Herbs and spices, flavours and fragrances | [92]Phillips et al. 1981:  Mouse liver tissue | [92]Phillips et al. 1981:  LC and NMR | |  |
| 177 | N^2^-isoestragole-dG  C_20_H_23_N_5_O_5_  413.169920 |  | Isoestragole, Alkenylbenzene  Herbs and spices, flavours and fragrances | [94]Paini et al. 2012:  Rat liver, lung and kidney tissue  [92]Phillips et al. 1981:  Mouse liver tissue | [94]Paini et al. 2012:  LC-MS/MS  [92]Phillips et al. 1981:  LC and NMR | |  |
| 178 | 7-isoestragole-dG  C_20_H_24_N_5_O_5_^+^  414.177745 |  | Isoestragole, Alkenylbenzene  Herbs and spices, flavours and fragrances | [93]Wiseman et al. 1985:  Incubation with dG (*In vitro*) | [93]Wiseman et al. 1985:  LC and NMR | |  |
| 179 | 8-isoestragole-dG  C_20_H_23_N_5_O_5_  413.169920 |  | Isoestragole, Alkenylbenzene  Herbs and spices, flavours and fragrances | [93]Wiseman et al. 1985:  Incubation with dG (*In vitro*) | [93]Wiseman et al. 1985:  LC and NMR | |  |
| 180 | N^2^-safrole-dG  C_20_H_21_N_5_O_6_  427.149185 |  | Safrole, Alkenylbenzene  Herbs and spices, flavours and fragrances | [93]Wiseman et al. 1985:  Mouse liver tissue | [93]Wiseman et al. 1985:  LC and NMR | |  |
| 181 | N^6^-isosafrole-dA  C_20_H_21_N_5_O_5_  411.154270 |  | Isosafrole, Alkenylbenzene  Herbs and spices, flavours and fragrances | [93]Wiseman et al. 1985:  Mouse liver tissue | [93]Wiseman et al. 1985:  LC and NMR | |  |
| 182 | N^2^-isosafrole-dG  C_20_H_21_N_5_O_6_  427.149185 |  | Isosafrole, Alkenylbenzene  Herbs and spices, flavours and fragrances | [95]Martati et al. 2014:  Rat liver tissue and human liver cells (HepG2) | [95]Martati et al. 2014:  LC-MS/MS | |  |
| 183 | 8-isosafrole-dG  C_20_H_22_N_5_O_6_^+^  427.149185 |  | Isosafrole, Alkenylbenzene  Herbs and spices, flavours and fragrances | [93]Wiseman et al. 1985:  Mouse liver tissue | [93]Wiseman et al. 1985:  LC and NMR | |  |
| 184 | 7-isosafrole-dG  C_20_H_21_N_5_O_6_  428.15646 |  | Isosafrole, Alkenylbenzene  Herbs and spices, flavours and fragrances | [93]Wiseman et al. 1985:  Mouse liver tissue | [93]Wiseman et al. 1985:  LC and NMR | |  |
| 185 | 7-OH-isosafrole-dG  7-Hydroxy-isosafrole-dG  C_20_H_24_N_5_O_7_^+^  446.167575 |  | Isosafrole, Alkenylbenzene  Herbs and spices, flavours and fragrances | [96]Shen et al. 2012:  Mouse urine | [96]Shen et al. 2012:  LC-MS/MS | |  |
| 186 | N^6^-Me-isoeugenol-dA  N^6^-Methyl-isoeugenol-dA  C_21_H_25_N_5_O_5_  427.185570 |  | Me-isoeugenol, Alkenylbenzene  Herbs and spices, flavours and fragrances | [97]Herrmann et al. 2012:  Herring sperm DNA and bacteria | [97]Herrmann et al. 2012:  LC–MS/MS | |  |
| 187 | N^2^-Me-isoeugenol-dG  N^2^-Methyl-isoeugenol-dG  C_21_H_25_N_5_O_6_  443.180485 |  | Me-isoeugenol, Alkenylbenzene  Herbs and spices, flavours and fragrances | [97]Herrmann et al. 2012:  Herring sperm and bacterial DNA | [97]Herrmann et al. 2012:  LC–MS/MS | |  |
| 188 | N^6^-AL-I-dA  N^6^-AristolactamI-dA  C_27_H_22_N_6_O_7_  542.154999 |  | Aristolochic acid  Herbs and spices | [56]Guo et al 2017:  Human kidney tissue  [98]Stiborová et al. 1994:  Rat fore-stomach tissue | [56]Guo et al 2017:  nanoLC-HRMS/MS  [98]Stiborová et al. 1994:  LC and ^32^P-postlabeling | |  |
| 189 | N^2^-AL-I-dG  N^2^-AristolactamI-dG  C_27_H_22_N_6_O_8_  558.149914 |  | Aristolochic acid  Herbs and spices | [98]Stiborová et al. 1994:  Rat fore-stomach tissue | [98]Stiborová et al. 1994:  LC and ^32^P-postlabeling | |  |
| 190 | N^6^-AL-II-dA  N^6^-AristolactamII-dA  C_26_H_20_N_6_O_6_  512.14443 |  | Aristolochic acid  Herbs and spices | [98]Stiborová et al. 1994:  Rat fore-stomach tissue | [98]Stiborová et al. 1994:  LC and ^32^P-postlabeling | |  |
| 191 | N^6^-POB-dA  N^6^-Pyridyl-oxobutyl-dA  C_19_H_22_N_6_O_4_  398.170251 |  | Nitrosamine, NOC, ROS  Tobacco, red and processed meat, beer and fish products | [99]Carlson et al 2018:  Rat lung and liver tissue | [99]Carlson et al 2018:  LC-MS/MS | |  |
| 192 | N^1^-POB-dI  N^1^-Pyridyl-oxobutyl-dI  C_19_H_21_N_5_O_5_  399.154270 |  | Nitrosamine, NOC, ROS  Tobacco, red and processed meat, beer and fish products | [99]Carlson et al 2018:  Calf Thymus DNA | [99]Carlson et al 2018:  LC-MS/MS | |  |
| 193 | O^6^-POB-dG  O^6^-Pyridyl-oxobutyl-dG  C_19_H_22_N_6_O_5_  414.165169 |  | Nitrosamine, NOC, ROS  Tobacco, red and processed meat, beer and fish products | [100]Lao et al. 2006:  Rat liver and lung tissue  [101]Stepanov, I. and Hecht, S. S. 2009:  Rat liver and lung tissue (mitocondrial DNA) | [100]Lao et al. 2006:  LC-MS/MS  [101]Stepanov, I. and Hecht, S. S. 2009:  LC-MS/MS | |  |
| 194 | 7-POB-dG  7-Pyridyl-oxobutyl-dG  C_19_H_23_N_6_O_5_^+^  415.172994 |  | Nitrosamine, NOC, ROS  Tobacco, red and processed meat, beer and fish products | [100]Lao et al. 2006:  Rat liver and lung tissue  [101]Stepanov, I. and Hecht, S. S. 2009:  Rat liver and lung tissue (mitocondrial DNA)  [102]Wang et al. 2003:  Calf thymus DNA | [100]Lao et al. 2006:  LC- MS/MS  [101]Stepanov, I. and Hecht, S. S. 2009:  LC-MS/MS  [102]Wang et al. 2003:  LC-MS | |  |
| 195 | O^2^-POB-dC  O^2^-Pyridyl-oxobutyl-dC  C_18_H_24_N_4_O_5_  376.17467 |  | Nitrosamine, NOC, ROS  Tobacco, red and processed meat, beer and fish products | [100]Lao et al. 2006:  Rat liver and lung tissue | [100]Lao et al. 2006:  LC-MS/MS | |  |
| 196 | 3-POB-dC  3-Pyridyl-oxobutyl-dC  C_18_H_25_N_4_O_5_^+^  377.18195 |  | Nitrosamine, NOC, ROS  Tobacco, red and processed meat, beer and fish products | [103]Michel et al 2017:  Calf Thymus DNA | [103]Michel et al 2017:  LC-MS/MS | |  |
| 197 | N^4^-POB-dC  N^4^-Pyridyl-oxobutyl-dC  C_18_H_24_N_4_O_5_  376.17467 |  | Nitrosamine, NOC, ROS  Tobacco, red and processed meat, beer and fish products | [103]Michel et al 2017:  Calf Thymus DNA | [103]Michel et al 2017:  LC-MS/MS | |  |
| 198 | O^2^-POB-dT  O^2^-Pyridyl-oxobutyl-dT  C_19_H_23_N_3_O_6_  389.158687 |  | Nitrosamine, NOC, ROS  Tobacco, red and processed meat, beer and fish products | [100]Lao et al. 2006:  Rat liver and lung tissue  [101]Stepanov, I. and Hecht, S. S. 2009:  Rat liver and lung tissue (mitocondrial DNA) | [100]Lao et al. 2006:  LC-MS/MS  [101]Stepanov, I. and Hecht, S. S. 2009:  LC-MS/MS | |  |
| 199 | O^4^-POB-dT  O4-Pyridyl-oxobutyl-dT  C_19_H_23_N_3_O_6_  389.158687 |  | Nitrosamine, NOC, ROS  Tobacco, red and processed meat, beer and fish products | [104]Leng et al. 2017:  Mammalian cells | [104]Leng et al. 2017:  nanoLC-MS/MS | |  |
| 200 | N^6^-PHB-dA  N^6^-Pyridyl-hydroxybutyl-dA  C_19_H_24_N_6_O_4_  400.18590 |  | Nitrosamine, NOC, ROS  Tobacco, red and processed meat, beer and fish products | [99]Carlson et al 2018:  Rat lung and liver tissue | [99]Carlson et al 2018:  LC-MS/MS | |  |
| 201 | O^6^-PHB-dG  O^6^-Pyridyl-hydroxy-butyl-dG  C_19_H_24_N_6_O_5_  416.180819 |  | Nitrosamine, NOC, ROS  Tobacco, red and processed meat, beer and fish products | [101]Stepanov, I. and Hecht, S. S. 2009:  Rat liver and lung tissue (mitocondrial DNA) | [101]Stepanov, I. and Hecht, S. S. 2009:  LC-MS/MS | |  |
| 202 | 7-PHB-dG  7-Pyridyl-hydroxy-butyl-dG  C_19_H_25_N_6_O_5_^+^  417.188644 |  | Nitrosamine, NOC, ROS  Tobacco, red and processed meat, beer and fish products | [101]Stepanov, I. and Hecht, S. S. 2009:  Rat liver and lung tissue (mitocondrial DNA)  [11]Carra` et al. 2019:  Mouse lung tissues | [101]Stepanov, I. and Hecht, S. S. 2009:  LC-MS/MS  [11]Carra` et al. 2019:  2D-LC-HRMS/MS  (tentatively identified) | |  |
| 203 | O^2^-PHB-dT  O^2^-Pyridyl-hydroxy-butyl-dT  C_19_H_25_N_3_O_6_  391.174337 |  | Nitrosamine, NOC, ROS  Tobacco, red and processed meat, beer and fish products | [101]Stepanov, I. and Hecht, S. S. 2009:  Rat liver and lung tissue (mitocondrial DNA)  [11]Carra` et al. 2019:  Mouse lung tissues | [101]Stepanov, I. and Hecht, S. S. 2009:  LC-MS/MS  [11]Carra` et al. 2019:  2D-LC-HRMS/MS  (tentatively identified) | |  |
| 204 | 8-(N'-APNH)-dG  8-(9-(4′-aminophenyl)-9H-pyrido[3,4-  b]indole)-dG  C_27_H_24_N_8_O_4_  524.192052  Aminophenylnorharman-dG |  | HAA  Tobacco, cooked meat, fish and poultry | [105]Totsuka et al. 2002:  Rat liver and colon tissue | [105]Totsuka et al. 2002:  ^32^P-postlabeling | | |
| 205 | N^6^-IQ-dA  N^6^-(2-amino-3-methylimidazo[4,5-f]quinolone)-dA  C_21_H_21_N_9_O_3_  447.176736 |  | HAA  Tobacco, cooked meat, fish and poultry | [106]Jamin et al. 2007: Incubation with dA (*In vitro*) | [106]Jamin et al. 2007:  LC-HRMS/MS (tentatively identified) | |  |
| 206 | N^2^-IQ-dG  N^2^-(2-amino-3-methylimidazo[4,5-f]quinolone)-dG  C_21_H_21_N_9_O_4_  463.171651 |  | HAA  Tobacco, cooked meat, fish and poultry | [107]Turesky et al. 1996a:  Rat colon, liver, and kidney tissue  [108]Turesky et al. 1996b:  Monkey liver, kidney, colon, heart and pancreas tissue | [107]Turesky et al. 1996a:  LC and ^32^P-postlabeling  [108]Turesky et al. 1996b:  ^32^P-postlabeling | |  |
| 207 | 8-(N'-IQ)-dG  8-(2-amino-3-methylimidazo[4,5-f]quinolone)-dG  C_21_H_21_N_9_O_4_  463.171651 |  | HAA  Tobacco, cooked meat, fish and poultry | [107]Turesky et al. 1996a:  Rat colon, liver, and kidney tissue  [108]Turesky et al. 1996b:  Monkey liver, kidney, colon, heart and pancreas tissue | [107]Turesky et al. 1996a:  LC and ^32^P-postlabeling  [108]Turesky et al. 1996b:  ^32^P-postlabeling | |  |
| 208 | 8-(N'-MeIQ)-dG  8-(2-amino-3,4-dimethylimidazo[4,5-f]quinolone)-dG  C_22_H_23_N_9_O_4_  477.187301 |  | HAA  Tobacco, cooked meat, fish and poultry | [109]Kim et al. 2016:  Mouse liver and colon tissue | [109]Kim et al. 2016:  LC-MS/MS | |  |
| 209 | 8-(N'-MeIQx)-dG  8-(2-amino-3,8-dimethylimidazo[4,5-f]quinoxaline)-dG  C_21_H_22_N_10_O_4_  478.182550 |  | HAA  Tobacco, cooked meat, fish and poultry | [110]Totsuka et al. 1996:  Human kidney, rectum and colon tissue  [111]Bessette et al. 2009:  Rat liver tissue | [110]Totsuka et al. 1996:  LC and ^32^P-postlabeling  [111]Bessette et al. 2009: capLC-MS/MS | |  |
| 210 | N^2^-MeIQx-dG  N^2^-(2-amino-3,8-dimethylimidazo[4,5-f]quinoxaline)-dG  C_21_H_22_N_10_O_4_  478.182550 |  | HAA  Tobacco, cooked meat, fish and poultry | [111]Bessette et al. 2009:  Rat liver tissue | [111]Bessette et al. 2009: capLC-MS/MS | |  |
| 211 | 8-(N'-DiMeIQx)-dG  8-(2-amino-3,4,8-trimethylimidazo[4,5-f]quinoxaline)-dG  C_22_H_24_N_10_O_4_  492.198200 |  | HAA  Tobacco, cooked meat, fish and poultry | [112]Frandsen et al. 1994:  Rat liver tissue, Calf Thymus DNA | [112]Frandsen et al. 1994:  LC and ^32^P-postlabelling | |  |
| 212 | 8-(N'-Trp-P-2)-dG  8-(2-amino-1-  methyl-5H-pyrido[4,3-b]indole)-dG  C_22_H_22_N_8_O_4_    462.176402 |  | HAA  Tobacco, Heated foods | [113]Hashimoto et al. 1982:  Rat liver tissue | [113]Hashimoto et al. 1982:  LC-UV | |  |
| 213 | 8-(N'-Glu-P-1)-dG  8-(2-amino-6-methyldiprido[1,2-a:3′,2′-d]imidazole)-dG  C_21_H_21_N_9_O_4_  463.171651 |  | HAA  Tobacco, Heated foods | [113]Hashimoto et al. 1982:  Rat liver tissue | [113]Hashimoto et al. 1982:  LC-UV | |  |
| 214 | 8-(N'-AαC)-dG  8-(2-Amino-9H-pyrido[2,3-b]indole)-dG  C_21_H_20_N_8_O_4_  448.160752 |  | HAA  Tobacco, cooked meat, fish and poultry | [109]Kim et al. 2016:  Mouse liver and colon tissue  [114]Frederiksen et al. 2004:  Calf thymus DNA | [109]Kim et al. 2016:  LC-MS/MS  [114]Frederiksen et al. 2004: LC-MS and ^32^P-postlabelling | |  |
| 215 | 8-(N'-MeAαC)-dG  8-(2-Amino-1-methyl-9H-pyrido[2,3-b]indole)-dG  C_22_H_22_N_8_O_4_  462.176402 |  | HAA  Tobacco, wine, cooked meat, fish and poultry | [114]Frederiksen et al. 2004: Rat liver, colon, and heart tissue, and calf thymus DNA | [114]Frederiksen et al. 2004: LC-MS and ^32^P-postlabelling | |  |
| 216 | 8-(N'-PhIP)-dG  8-(2-amino-1- methyl-6-phenylimidazo[4,5-b]pyridine)-dG  C_23_H_23_N_9_O_4_  489.187301 |  | HAA  Tobacco, cooked meat, fish and poultry | [115]Xiao et al 2016:  Human prostate tissue  [56]Guo et al 2017:  Human prostate tissue | [115]Xiao et al 2016:  nanoLC‐HRMS/MS  [56]Guo et al 2017:  nanoLC‐HRMS/MS | |  |
| 217 | 8-(N’-2MA)-dA  8-(N’-2Methylaniline)-dA  C_17_H_20_N_6_O_3_  356.159689 |  | AA  Pollution | [116]Jones et al. 2003:  Calf Thymus DNA  [55]Guo et al 2018:  Calf Thymus DNA | | [116]Jones et al. 2003:  LC-MS/MS  [55]Guo et al 2018:  nanoLC-HRMS/MS (tentatively identified) | |
| 218 | N^6^-(2-MA)-dA  N^6^-(2-Methylaniline)-dA  C_17_H_20_N_6_O_3_  356.159689 |  | AA  Pollution | [55]Guo et al 2018:  Calf Thymus DNA | | [55]Guo et al 2018:  nanoLC-HRMS/MS (tentatively identified) | |
| 219 | 8-(N’-4MA)-dA  8-(N’-4Methylaniline)-dA  C_17_H_20_N_6_O_3_  356.159689 |  | AA  Pollution | [116]Jones et al. 2003:  Calf Thymus DNA | | [116]Jones et al. 2003:  LC-MS/MS | |
| 220 | 8-(N’-2MA)-dG  8-(N’-2-Methylaniline)-dG  C_17_H_20_N_6_O_4_  372.154604 |  | AA  Tobacco, Pollution | [116]Jones et al. 2003:  Calf Thymus DNA  [55]Guo et al 2018:  Calf Thymus DNA | | [116]Jones et al. 2003:  LC-MS/MS  [55]Guo et al 2018:  nanoLC-HRMS/MS | |
| 221 | 8-(N’-4MA)-dG  8-(N’-4-Methylaniline)-dG  C_17_H_20_N_6_O_4_  372.154604 |  | AA  Tobacco, Pollution | [116]Jones et al. 2003:  Calf Thymus DNA | | [116]Jones et al. 2003:  LC-MS/MS | |
| 222 | 8-(N’-2CA)-dA  8-(N’-2-Chloroaniline)-dA  C_16_H_17_ClN_6_O_3_  376.10507 |  | AA  Pollution | [116]Jones et al. 2003:  Calf Thymus DNA | | [116]Jones et al. 2003:  LC-MS/MS | |
| 223 | 8-(N’-4-CA)-dA  8-(N’-4-Chloroaniline)-dA  C_16_H_17_ClN_6_O_3_  376.10507 |  | AA  Pollution | [116]Jones et al. 2003:  Calf Thymus DNA | | [116]Jones et al. 2003:  LC-MS/MS | |
| 224 | 8-(N’-2-CA)-dG  8-(N’-2-Chloroaniline)-dG  C_16_H_17_ClN_6_O_4_  392.099985 |  | AA  Pollution | [116]Jones et al. 2003:  Calf Thymus DNA | | [116]Jones et al. 2003:  LC-MS/MS | |
| 225 | 8-(N’-4-CA)-dG  8-(N’-4-Chloroaniline)-dG  C_16_H_17_ClN_6_O_4_  392.09998 |  | AA  Pollution | [116]Jones et al. 2003:  Calf Thymus DNA | | [116]Jones et al. 2003:  LC-MS/MS | |
| 226 | N^6^-(2,6-DMA)-dA  N^6^-(2,6-Dimethylaniline)-dA  C_18_H_22_N_6_O_3_  370.175339 |  | AA  Pollution | [55]Guo et al 2018:  Human bladder tissue  [116]Jones et al. 2003:  Calf Thymus DNA | | [55]Guo et al 2018:  nanoLC-HRMS/MS  [116]Jones et al. 2003:  LC-MS/MS | |
| 227 | 8-(N’-3,5-DMA)-dA  8-( N’-3,5-Dimethylaniline)-dA  C_18_H_22_N_6_O_3_  370.175339 |  | AA  Pollution | [117]Cui et al. 2007:  Calf thymus DNA | | [117]Cui et al. 2007:  LC-HRMS/MS and NMR | |
| 228 | N^6^-(3,5-DMA)-dA  N^6^-(3,5-Dimethylaniline)-dA  C_18_H_22_N_6_O_3_  370.175339 |  | AA  Pollution | [117]Cui et al. 2007:  Calf thymus DNA | | [117]Cui et al. 2007:  LC-HRMS/MS and NMR | |
| 229 | 8-(N’-2,4-DMA)-dA  8-(N’-2,4-Dimethylaniline)-dA  C_18_H_22_N_6_O_3_  370.175339 |  | AA  Pollution | [116]Jones et al. 2003:  Calf Thymus DNA | | [116]Jones et al. 2003:  LC-MS/MS | |
| 230 | 8-(N’-2,6-DMA)-dA  8-(N’-2,6-Dimethylaniline)-dA  C_18_H_22_N_6_O_3_  370.175339 |  | AA  Pollution | [116]Jones et al. 2003:  Calf Thymus DNA | | [116]Jones et al. 2003:  LC-MS/MS | |
| 231 | 8-(N’-3,5-DMA)-dG  8-(N’-3,5-Dimethylaniline)-dG  C_18_H_22_N_6_O_4_  386.170254 |  | AA  Pollution | [117]Cui et al. 2007:  Calf thymus DNA | | [117]Cui et al. 2007:  LC-HRMS/MS and NMR | |
| 232 | 8-(N’-2,4-DMA)-dG  8-(N’-2,4-Dimethylaniline)-dG  C_18_H_22_N_6_O_4_  386.170254 |  | AA  Pollution | [116]Jones et al. 2003:  Calf Thymus DNA | | [116]Jones et al. 2003:  LC-MS/MS | |
| 233 | 8-(N’-2,6-DMA)-dG  8-(N’-2,6-Dimethylaniline)-dG  C_18_H_22_N_6_O_4_  386.170254 |  | AA  Pollution | [116]Jones et al. 2003:  Calf Thymus DNA  [55]Guo et al 2018:  Calf Thymus DNA | | [116]Jones et al. 2003:  LC-MS/MS  [55]Guo et al 2018:  nanoLC-HRMS/MS | |
| 234 | 5-(N’-3,5DMA)-dC  5-(N’-3,5-Dimethylaniline)-dC  C_17_H_22_N_4_O_4_  346.164106 |  | AA  Pollution | [117]Cui et al. 2007:  Calf thymus DNA | | [117]Cui et al. 2007:  LC-HRMS/MS and NMR | |
| 235 | 8-(N'-Benzidine)-dG  C_22_H_23_N_7_O_4_  449.181153 |  | AA  Pollution | [118]Yamazoe et al. 1986:  Calf thymus DNA | | [118]Yamazoe et al. 1986:  LC-UV/MS and NMR | |
| 236 | 8-(N'-Ac-benzidine)-dG  8-(N'-Acetylbenzidine)-dG  C_24_H_25_N_7_O_5_  491.191718 |  | AA  Tobacco, cooked meat, fish and poultry | [119]Martin et al. 1982:  Rat and mouse liver tissue | | [119]Martin et al. 1982:  LC-UV/MS and NMR | |
| 237 | 8-(N'-ABP)-dA  8-(N'-Aminobiphenyl)-dA  C_22_H_22_N_6_O_3_  418.175339 |  | AA  Tobacco, heated foods, food preservation, cooked meat, pollution | [55]Guo et al 2018:  Human bladder tissue | | [55]Guo et al 2018:  nanoLC-HRMS/MS (tentatively identified) | |
| 238 | 8-(N'-ABP)-dG  8-(N'-Aminobiphenyl)-dG  C_22_H_22_N_6_O_4_  434.170254 |  | AA  Tobacco, heated foods, food preservation, cooked meat, pollution | [111]Bessette et al. 2009:  Liver cells  [55]Guo et al 2018:  Human bladder tissue | | [111]Bessette et al. 2009: capLC-MS/MS  [55]Guo et al 2018:  nanoLC-HRMS/MS | |
| 239 | N^2^-ABP-dG  N^2^-Aminobiphenyl-dG  C_22_H_22_N_6_O_4_  434.170254 |  | AA  Tobacco, heated foods, food preservation, cooked meat, pollution | [55]Guo et al 2018:  Human bladder tissue | | [55]Guo et al 2018:  nanoLC-HRMS/MS  (tentatively identified) | |
| 240 | N^2^-DMABP-dG  N^2^-Dimethyl-aminobiphenyl-dG  C_24_H_26_N_6_O_4_  462.201554 |  | AA  Tobacco, heated foods, Pollution | [120]Westra et al. 1985:  Rat liver and intestine tissue | | [120]Westra et al. 1985:  LC-UV/MS | |
| 241 | 8-(N'-DMABP)-dG  8-(N'-Dimethyl-aminobiphenyl)-dG  C_24_H_26_N_6_O_4_  462.201554 |  | AA  Tobacco, heated foods, food preservation, Pollution | [120]Westra et al. 1985:  Rat liver and intestine tissue | | [120]Westra et al. 1985:  LC-UV/MS | |
| 242 | 8-(N’-AB)-dG  8-(N’-Aminoazobenzene)-dG  C_22_H_22_N_8_O_4_  462.176402 |  | AA  Pollution | [121]Tullis et al. 1987:  Rat liver, spleen and kidney tissue | | [121]Tullis et al. 1987:  LC-UV/MS  and NMR | |
| 243 | N^6^-MAB-dA  N^6^-Methyl-aminoazobenzene-dA  C_23_H_24_N_8_O_3_  460.197137 |  | AA  Pollution | [121]Tullis et al. 1987:  Rat liver, spleen and kidney tissue | | [121]Tullis et al. 1987:  LC-UV/MS  and NMR | |
| 244 | N^2^-MAB-dG  N^2^-Methyl-aminoazobenzene-dG  C_23_H_24_N_8_O_4_  476.192052 |  | AA  Pollution | [121]Tullis et al. 1987:  Rat liver, spleen and kidney tissue | | [121]Tullis et al. 1987:  LC-UV/MS  and NMR | |
| 245 | 8-(N’-MAB)-dG  8-(N’-Methyl-aminoazobenzene)-dG  C_23_H_24_N_8_O_4_  476.192052 |  | AA  Pollution | [121]Tullis et al. 1987:  Rat liver, spleen and kidney tissue | | [121]Tullis et al. 1987:  LC-UV/MS  and NMR | |
| 246 | N^6^-NA-dA  N^6^-Naphthylamine-dA  C_20_H_20_N_6_O_3_  392.159689 |  | AA  Tobacco, heated foods, Pollution | [55]Guo et al 2018:  Calf thymus DNA | | [55]Guo et al 2018:  nanoLC-HRMS/MS | |
| 247 | N^2^-NA-dG  N^2^-Naphthylamine-dG  C_20_H_20_N_6_O_4_  408.154604 |  | AA  Tobacco, heated foods, Pollution | [55]Guo et al 2018:  Calf thymus DNA | | [55]Guo et al 2018:  nanoLC-HRMS/MS | |
| 248 | O^6^-(N'-NA)-dG  O^6^-(N’-Naphthylamine)-dG  C_20_H_20_N_6_O_4_  408.154604 |  | AA  Tobacco, heated foods, Pollution | [122]Kadlubar et al. 1978:  Calf thymus DNA | | [122]Kadlubar et al. 1978:  LC-UV/MS and NMR | |
| 249 | 8-(N’-NA)-dG  8-(N’-Naphthylamine)-dG  C_20_H_20_N_6_O_4_  408.154604 |  | AA  Tobacco, heated foods, Pollution | [55]Guo et al 2018:  Calf thymus DNA | | [55]Guo et al 2018:  nanoLC-HRMS/MS | |
| 250 | O^6^-NA-dG  O^6^-Naphthylamine-dG  C_20_H_20_N_6_O_4_  408.154604 |  | AA  Tobacco, heated foods, Pollution | [123]Kadlubar et al. 1980:  Calf thymus DNA | | [123]Kadlubar et al. 1980:  LC-UV/MS and NMR | |
| 251 | 8-(N'-AF)-dG  8-(N'-Aminofluorene)-dG  C_23_H_22_N_6_O_4_  446.170254 |  | AA  Pollution | [124]Levy et al 1989:  Mouse liver tissue | | [124]Levy et al 1989:  LC-UV and ^32^P-postlabeling | |
| 252 | N^2^-NAQI-dG  N^2^-Amino-naphthoquinoneimine-dG  C_20_H_18_N_6_O_5_  422.133869 |  | AA  Tobacco, heated foods, Pollution | [125]Yamazoe et al. 1985:  Dog bladder and liver tissue | | [125]Yamazoe et al. 1985:  LC and NMR | |
| 253 | N^6^-MP-dA  N^6^-Methylpyrene-dA  C_27_H_23_N_5_O_3_  465.180090 |  | PAH  Pollution,  tobacco, charred or grilled foods | [126]Monien et al. 2008:  Rat liver tissue | [126]Monien et al. 2008:  LC-MS/MS and ^32^P-postlabeling | |  |
| 254 | N^2^-MP-dG  N^2^-Methylpyrene-dG  C_27_H_23_N_5_O_4_  481.175005 |  | PAH  Pollution,  tobacco, charred or grilled foods | [126]Monien et al. 2008:  Rat liver tissue | [126]Monien et al. 2008:  LC-MS/MS and ^32^P-postlabeling | |  |
| 255 | 7-B[a]P-dA  7-Benzo[a]pyrene-dA  C_30_H_24_N_5_O_3_^+^  502.18737 |  | PAH  Pollution,  tobacco, charred or grilled foods | [127]Devanesan et al. 1992:  Calf Thymus DNA | [127]Devanesan et al. 1992:  LC-FLNS and ^32^P-postlabeling | |  |
| 256 | 7-B[a]P-dG  7-Benzo[a]pyrene-dG  C_30_H_24_N_5_O_4_^+^  518.18228 |  | PAH  Pollution,  tobacco, charred or grilled foods | [127]Devanesan et al. 1992:  Calf Thymus DNA | [127]Devanesan et al. 1992:  LC-FLNS and ^32^P-postlabeling | |  |
| 257 | 8-B[a]P-dG  8-Benzo[a]pyrene-dG  C_30_H_23_N_5_O_4_  517.17500 |  | PAH  Pollution,  tobacco, charred or grilled foods | [127]Devanesan et al. 1992:  Calf Thymus DNA | [127]Devanesan et al. 1992:  LC-FLNS and ^32^P-postlabeling | |  |
| 258 | 7-B[a]PDE- dA  7-Benzo[a]pyrene-diolepoxide-dA  C_30_H_28_N_5_O_6_^+^  554.203960 |  | PAH  Pollution,  tobacco, charred or grilled foods | [127]Devanesan et al. 1992:  Calf Thymus DNA | [127]Devanesan et al. 1992:  LC-FLNS and ^32^P-postlabeling | |  |
| 259 | N^6^-B[a]PDE-dA  N^6^-Benzo[a]pyrene-diolepoxide-dA  C_30_H_27_N_5_O_6_  553.196135 |  | PAH  Pollution,  tobacco, charred or grilled foods | [128]Singh et al. 2010:  Calf thymus DNA | [128]Singh et al. 2010:  LC-MS/MS | |  |
| 260 | N^2^-B[a]PDE-dG  N^2^-Benzo[a]pyrene-diolepoxide-dG  C_30_H_27_N_5_O_7_  569.191050 |  | PAH  Pollution,  tobacco, charred or grilled foods | [129]Villalta et al. 2017:  Human lung tissue  [128]Singh et al. 2010:  Calf thymus DNA | [129]Villalta et al. 2017:  nanoLC-HRMS/MS  [128]Singh et al. 2010:  LC-MS/MS | |  |
| 261 | 7-B[a]PDE-dG  7-Benzo[a]pyrene-diolepoxide-dG  C_30_H_28_N_5_O_7_^+^  570.198352 |  | PAH  Pollution,  tobacco, charred or grilled foods | [127]Devanesan et al. 1992:  Calf Thymus DNA | [127]Devanesan et al. 1992:  LC-FLNS and ^32^P-postlabeling | |  |
| 262 | N^4^-B[a]PDE-dC  N^4^-Benzo[a]pyrene-diolepoxide-dC  C_29_H_27_N_3_O_7_  529.184902 |  | PAH  Pollution,  tobacco, charred or grilled foods | [128]Singh et al. 2010:  Calf thymus DNA | [128]Singh et al. 2010:  LC-MS/MS | |  |
| 263 | N^6^-DB[a,l]PDE-dA  N^6^-Dibenzo[a,l ]pyrene- diolepoxide-dA  C_34_H_29_N_5_O_6_  603.211785 |  | PAH  Pollution,  tobacco, charred or grilled foods | [128]Singh et al. 2010:  Calf thymus DNA | [128]Singh et al. 2010:  LC-MS/MS | |  |
| 264 | N^2^-DB[a,l]PDE-dG  N^2^-Dibenzo[a,l ]pyrene- diolepoxide-dG  C_34_H_29_N_5_O_7_  619.206700 |  | PAH  Pollution,  tobacco, charred or grilled foods | [128]Singh et al. 2010:  Calf thymus DNA | [128]Singh et al. 2010:  LC-MS/MS | |  |
| 265 | N^4^-DB[a,l]PDE-dC  N^4^-Dibenzo[a,l ]pyrene- diolepoxide-dC  C_33_H_29_N_3_O_7_  579.200552 |  | PAH  Pollution,  tobacco, charred or grilled foods | [128]Singh et al. 2010:  Calf thymus DNA | [128]Singh et al. 2010:  LC-MS/MS | |  |
| 266 | N^6^-B[b]FDE-dA  N^6^-Benzo  [b]ﬂuoranthene- diolepoxide-dA  C_30_H_27_N_5_O_6_  553.196135 |  | PAH  Pollution,  tobacco, charred or grilled foods | [128]Singh et al. 2010:  Calf thymus DNA | [128]Singh et al. 2010:  LC-MS/MS | |  |
| 267 | N^2^-B[b]FDE-dG  N^2^-Benzo  [b]ﬂuoranthene- diolepoxide-dG  C_30_H_27_N_5_O_7_  569.191050 |  | PAH  Pollution,  tobacco, charred or grilled foods | [128]Singh et al. 2010:  Calf thymus DNA | [128]Singh et al. 2010:  LC-MS/MS | |  |
| 268 | N^4^-B[b]FDE-dC  N^4^-Benzo  [b]ﬂuoranthene- diolepoxide-dC  C_29_H_27_N_3_O_7_  529.184902 |  | PAH  Pollution,  tobacco, charred or grilled foods | [128]Singh et al. 2010:  Calf thymus DNA | [128]Singh et al. 2010:  LC-MS/MS | |  |
| 269 | N^6^-DB[a,h]ADE-dA  N^6^-Dibenzo[a,h]anthracene- diolepoxide-dA  C_32_H_29_N_5_O_6_  579.211785 |  | PAH  Pollution,  tobacco, charred or grilled foods | [128]Singh et al. 2010:  Calf thymus DNA | [128]Singh et al. 2010:  LC-MS/MS | |  |
| 270 | N^2^-DB[a,h]ADE-dG  N^2^-Dibenzo[a,h]anthracene-diolepoxide-dG  C_32_H_29_N_5_O_7_  595.206700 |  | PAH  Pollution,  tobacco, charred or grilled foods | [128]Singh et al. 2010:  Calf thymus DNA | [128]Singh et al. 2010:  LC-MS/MS | |  |
| 271 | N^4^-DB[a,h]ADE-dC  N^4^-Dibenzo[a,h]anthracene-diolepoxide-dC  C_31_H_29_N_3_O_7_  555.200552 |  | PAH  Pollution,  tobacco, charred or grilled foods | [128]Singh et al. 2010:  Calf thymus DNA | [128]Singh et al. 2010:  LC-MS/MS | |  |
| 272 | N^6^-(5-Me-CDE)-dA  N^6^-(5-Methyl-chrysene-diolepoxide)-dA  C_29_H_29_N_5_O_6_  543.211785 |  | PAH  Pollution,  tobacco, charred or grilled foods | [130]Reardon et al. 1987:  Calf thymus DNA | [130]Reardon et al. 1987:  LC-UV and NMR | |  |
| 273 | N^6^-(5-Me-CDE)-dG  N^6^-(5-Methyl-chrysene-diolepoxide)-dG  C_29_H_29_N_5_O_7_  559.206700 |  | PAH  Pollution,  tobacco, charred or grilled foods | [130]Reardon et al. 1987:  Calf thymus DNA | [130]Reardon et al. 1987:  LC-UV and NMR | |  |
| 274 | N^6^-(5,6-DiMe-CDE)-dA  N^6^-(5,6-Dimethyl-chrysene-diolepoxide)-dA  C_30_H_31_N_5_O_6_  557.227435 |  | PAH  Pollution,  tobacco, charred or grilled foods | [131]Szeliga et al. 1997:  Calf thymus DNA | [131]Szeliga et al. 1997:  LC-UV and NMR | |  |
| 275 | N^6^-(5,6-DiMe-CDE)--dG  N^6^-(5,6-Dimethyl-chrysene-diolepoxide)-dG  C_30_H_31_N_5_O_7_  573.222350 |  | PAH  Pollution,  tobacco, charred or grilled foods | [131]Szeliga et al. 1997:  Calf thymus DNA | [131]Szeliga et al. 1997:  LC-UV and NMR | |  |
| 276 | N^6^-(2-OHE1)-dA  N^6^-(2-Hydroxy-estrone)-dA  C_28_H_33_N_5_O_6_  535.24308 |  | Estrogen, ROS  Endogenous sources | [132]Stack et al. 1996:  Incubation with dG (*in vitro*) | [132]Stack et al. 1996:  LC-MS/MS and NMR | | |
| 277 | N^2^-(2-OHE1)-dG  N^2^-(2-Hydroxy-estrone)-dG  C_28_H_33_N_5_O_7_  551.23800 |  | Estrogen, ROS  Endogenous sources | [132]Stack et al. 1996:  Incubation with dG (*in vitro*) | [132]Stack et al. 1996:  LC-MS/MS and NMR | | |
| 278 | 7-(4-OHE1)-dG  7-(4-Hydroxy-estrone)-dG  C_28_H_34_N_5_O_7_^+^  552.24527 |  | Estrogen, ROS  Endogenous sources | [132]Stack et al. 1996:  Incubation with dG (*in vitro*) | [132]Stack et al. 1996:  LC-MS/MS and NMR | | |
| 279 | 7-(4-OHE2)-dG  7-(4-Hydroxy-estradiol)-dG  C_28_H_36_N_5_O_7_^+^  554.261475 |  | Estrogen, ROS  Endogenous sources | [132]Stack et al. 1996:  Incubation with dG (*in vitro*) | [132]Stack et al. 1996:  LC-MS/MS and NMR | | |

Notes:
a) For the DNA adducts in which the core of the nucleobase has been modified, the original nucleobase is specified in the column “DNA adduct identity” instead of the alternative name.

b) The name of the sources have been abbreviated as follows: aromatic amines (AA), dehydropyrrolizidine (DHP), heterocyclic aromatic amines HAAs (HAA), lipid peroxidation products (LPO), N-nitro compound (NOC), polycyclic aromatic hydrocarbons (PAH), reactive nitrogen species (RNS), reactive nitrogen species (ROS), smoking tobacco (tobacco).

c) The name of the techniques have been abbreviated as follows: capillary liquid chromatography (capLC), fluorescence spectrometry (FLNS), gas chromatography (GC), high resolution mass spectrometry (HRMS), liquid chromatography (LC), mass spectrometry (MS), tandem mass spectrometry (MS/MS), nano liquid chromatrography (nanoLC),nuclear magnetic resonance (NMR), thin layer chromatrography (TLC), Ultraviolet (UV).

**References:**

[1] Hsu WY, Chen WTL, Lin W De, Tsai FJ, Tsai Y, Lin CT, et al. Analysis of urinary nucleosides as potential tumor markers in human colorectal cancer by high performance liquid chromatography/electrospray ionization tandem mass spectrometry. Clin Chim Acta 2009;402:31–7. https://doi.org/10.1016/j.cca.2008.12.009.

[2] Pang B, Zhou X, Yu H, Dong M, Taghizadeh K, Wishnok JS, et al. Lipid peroxidation dominates the chemistry of DNA adduct formation in a mouse model of inflammation. Carcinogenesis 2007;28:1807–13. https://doi.org/10.1093/CARCIN/BGM037.

[3] Mangerich A, Knutson CG, Parry NM, Muthupalani S, Ye W, Prestwich E, et al. Infection-induced colitis in mice causes dynamic and tissue-specific changes in stress response and DNA damage leading to colon cancer. Proc Natl Acad Sci 2012;109:E1820–9. https://doi.org/10.1073/PNAS.1207829109.

[4] Cadet J, Berger M, Raoul S, Buchko GW, Joshi PC, Ravanat JL. 2,2-Diamino-4-[(3,5-di-O-acetyl-2-deoxy-β-D-erythro-pentofuranosyl)anrino]-5-(2H)-oxazolone: A Novel and Predominant Radical Oxidation Product of 3’,5’-Di-O-acetyl-2’-deoxyguanosine. J Am Chem Soc 1994;116:7403–4. https://doi.org/10.1021/ja00095a052.

[5] Hwa Yun B, Geacintov NE, Shafirovich V. Generation of Guanine-Thymidine Cross-links in DNA by Peroxynitrite/Carbon Dioxide n.d. https://doi.org/10.1021/tx200139c.

[6] Raoul S, Cadet J. Photosensitized reaction of 8-oxo-7,8-dihydro-2′-deoxyguanosine: Identification of 1-(2-deoxy-β-D-erythro-pentofuranosyl)cyanuric acid as the major singlet oxygen oxidation product. J Am Chem Soc 1996;118:1892–8. https://doi.org/10.1021/ja952347l.

[7] Girault I, Fort S, Molko D, Cadet J. Ozonolysis of 2’-deoxycytidine: Isolation and identification of the main oxidation products. Free Radic Res 1997;26:257–66. https://doi.org/10.3109/10715769709097804.

[8] Dizdaroglu M, Jaruga P. Mechanisms of free radical-induced damage to DNA. Free Radic Res 2012;46:382–419. https://doi.org/10.3109/10715762.2011.653969.

[9] Nackerdien Z, Olinski R, Dizdaroglu M. DNA base damage in chromatin of γirradiated cultured human cells. Free Radic Res 1992;16:259–73. https://doi.org/10.3109/10715769209049179.

[10] Hu J, De Souza-Pinto NC, Haraguchi K, Hogue BA, Jaruga P, Greenberg MM, et al. Repair of formamidopyrimidines in DNA involves different glycosylases: Role of the OGG1, NTH1, and NEIL1 enzymes. J Biol Chem 2005;280:40544–51. https://doi.org/10.1074/jbc.M508772200.

[11] Carrà A, Guidolin V, Dator RP, Upadhyaya P, Kassie F, Villalta PW, et al. Targeted High Resolution LC/MS3 Adductomics Method for the Characterization of Endogenous DNA Damage. Front Chem 2019;7:658. https://doi.org/10.3389/fchem.2019.00658.

[12] Badouard C, Masuda M, Nishino H, Cadet J, Favier A, Ravanat JL. Detection of chlorinated DNA and RNA nucleosides by HPLC coupled to tandem mass spectrometry as potential biomarkers of inflammation. J Chromatogr B 2005;827:26–31. https://doi.org/10.1016/J.JCHROMB.2005.03.025.

[13] Jiang Q, Blount BC, Ames BN. 5-Chlorouracil, a marker of DNA damage from hypochlorous acid during inflammation: A gas chromatography-mass spectrometry assay. J Biol Chem 2003;278:32834–40. https://doi.org/10.1074/jbc.M304021200.

[14] Henderson JP, Byun J, Mueller DM, Heinecke JW. The eosinophil peroxidase-hydrogen peroxide-bromide system of human eosinophils generates 5-bromouracil, a mutagenic thymine analogue. Biochemistry 2001;40:2052–9. https://doi.org/10.1021/bi002015f.

[15] Ma B, Jing M, Villalta PW, Kapphahn RJ, Montezuma SR, Ferrington DA, et al. Simultaneous determination of 8-oxo-2’-deoxyguanosine and 8-oxo-2’-deoxyadenosine in human retinal DNA by liquid chromatography nanoelectrospray-tandem mass spectrometry OPEN. Nat Publ Gr 2016. https://doi.org/10.1038/srep22375.

[16] Frelon S, Douki T, Ravanat JL, Pouget JP, Tornabene C, Cadet J. High-Performance Liquid Chromatography−Tandem Mass Spectrometry Measurement of Radiation-Induced Base Damage to Isolated and Cellular DNA. Chem Res Toxicol 2000;13:1002–10. https://doi.org/10.1021/TX000085H.

[17] Cooke MS, Hu CW, Chang YJ, Chao MR. Urinary DNA adductomics – A novel approach for exposomics. Environ Int 2018;121:1033–8. https://doi.org/10.1016/j.envint.2018.10.041.

[18] Chou PH, Kageyama S, Matsuda S, Kanemoto K, Sasada Y, Oka M, et al. Detection of lipid peroxidation-induced DNA adducts caused by 4-Oxo-2(E)-nonenal and 4-Oxo-2(E)-hexenal in human autopsy tissues. Chem Res Toxicol 2010;23:1442–8. https://doi.org/10.1021/tx100047d.

[19] Ravanatf J-L, Cadet J. Reaction of Singlet Oxygen with 2-Deoxyguanosine and DNA. Isolation and Characterization of the Main Oxidation Products. Chem Res Toxicol 1995;8:379–88.

[20] Douki T, Delateur T, Bianchini F, Cadet J. Observation and prevention of an artefactual formation of oxidized DNA bases and nucleosides in the GC-EIMS method. Carcinogenesis 1996;17:347–53. https://doi.org/10.1093/CARCIN/17.2.347.

[21] Tang Y, Wang Z, Li M, Zhang R, Zhang J. Simultaneous quantitation of 14 DNA alkylation adducts in human liver and kidney cells by UHPLC-MS/MS: Application to profiling DNA adducts of genotoxic reagents. J Pharm Biomed Anal 2019;166:387–97. https://doi.org/10.1016/J.JPBA.2019.01.034.

[22] Chang YJ, Cooke MS, Hu CW, Chao MR. Novel approach to integrated DNA adductomics for the assessment of in vitro and in vivo environmental exposures. Arch Toxicol 2018;92:2665–80. https://doi.org/10.1007/s00204-018-2252-6.

[23] Lu K, Craft S, Nakamura J, Moeller BC, Swenberg JA. Use of LC-MS/MS and Stable Isotopes to Differentiate Hydroxymethyl and Methyl DNA Adducts from Formaldehyde and Nitrosodimethylamine. Chem Res Toxicol 2012;25:664. https://doi.org/10.1021/TX200426B.

[24] Leu RK Le, Winter JM, Christophersen CT, Young GP, Humphreys KJ, Hu Y, et al. Butyrylated starch intake can prevent red meat-inducedO6-methyl-2-deoxyguanosine adducts in human rectal tissue: a randomisedclinical trial. Br J Nutr 2015;114:220. https://doi.org/10.1017/S0007114515001750.

[25] Liu J, Jiang J, Mo J, Liu D, Cao D, Wang H, et al. Global DNA 5-Hydroxymethylcytosine and 5-Formylcytosine Contents Are Decreased in the Early Stage of Hepatocellular Carcinoma. Hepatology 2019;69:196–208. https://doi.org/10.1002/HEP.30146.

[26] Chen HJC, Liu YF. Simultaneous quantitative analysis of N3-ethyladenine and N7-ethylguanine in human leukocyte deoxyribonucleic acid by stable isotope dilution capillary liquid chromatography-nanospray ionization tandem mass spectrometry. J Chromatogr A 2013;1271:86–94. https://doi.org/10.1016/j.chroma.2012.11.033.

[27] Guidolin V, Carlson ES, Carrà A, Villalta PW, Maertens LA, Hecht SS, et al. Identification of New Markers of Alcohol-Derived DNA Damage in Humans. Biomolecules 2021;11:1–20. https://doi.org/10.3390/BIOM11030366.

[28] Matsuda T, Yabushita H, Kanaly RA, Shibutani S, Yokoyama A. Increased DNA damage in ALDH2-deficient alcoholics. Chem Res Toxicol 2006;19:1374–8. https://doi.org/10.1021/tx060113h.

[29] Balbo S, Villalta PW, Hecht SS. Quantitation of 7-ethylguanine in leukocyte DNA from smokers and nonsmokers by liquid chromatography-nanoelectrospray-high resolution tandem mass spectrometry. Chem Res Toxicol 2011;24:1729–34. https://doi.org/10.1021/tx200262d.

[30] Hub NH, Satofa MS, Shiga J, Rajewsky MF, Kuroki T. Immunoanalytical Detection of O4-Ethylthymine in Liver DNA of Individuals with or Without Malignant Tumors. Cancer Res 1989;49:93–7.

[31] Chen H-JC, Wang Y-C, Lin W-P. Analysis of Ethylated Thymidine Adducts in Human Leukocyte DNA by Stable Isotope Dilution Nanoflow Liquid Chromatography− Nanospray Ionization Tandem Mass Spectrometry 2012. https://doi.org/10.1021/ac203405y.

[32] Chen HJC, Lee CR. Detection and simultaneous quantification of three smoking-related ethylthymidine adducts in human salivary DNA by liquid chromatography tandem mass spectrometry. Toxicol Lett 2014;224:101–7. https://doi.org/10.1016/J.TOXLET.2013.10.002.

[33] Bonfanti M, Magagnotti C, Galli A, Bagnati R, Moret M, Gariboldi P, et al. Determination of O6-Butylguanine in DNA by Immunoaffinity Extraction/Gas Chromatography-Mass Spectrometry. Cancer Res 1990;50.

[34] Chang YJ, Cooke MS, Chen YR, Yang SF, Li PS, Hu CW, et al. Is high resolution a strict requirement for mass spectrometry-based cellular DNA adductomics? Chemosphere 2021;274. https://doi.org/10.1016/J.CHEMOSPHERE.2021.129991.

[35] Li F, Segal A, Solomon JJ. In vitro reaction of ethylene oxide with DNA and characterization of DNA adducts. Chem Biol Interact 1992;83:35–54. https://doi.org/10.1016/0009-2797(92)90090-8.

[36] Nakao LS, Fonseca E, Augusto O. Detection of C8-(1-Hydroxyethyl)guanine in Liver RNA and DNA from Control and Ethanol-Treated Rats. Chem Res Toxicol 2002;15:1248–53. https://doi.org/10.1021/TX0255166.

[37] Wu KY, Scheller N, Ranasinghe A, Yen TY, Sangaiah R, Giese R, et al. A Gas Chromatography/Electron Capture/Negative Chemical Ionization High-Resolution Mass Spectrometry Method for Analysis of Endogenous and Exogenous N7-(2-Hydroxyethyl)guanine in Rodents and Its Potential for Human Biological Monitoring. Chem Res Toxicol 1999;12:722–9. https://doi.org/10.1021/TX990059N.

[38] Wang M, Lao Y, Cheng G, Shi Y, Villalta PW, Hecht SS. Identification of Adducts Formed in the Reaction of α-Acetoxy-N-nitrosopyrrolidine with Deoxyribonucleosides and DNA. Chem Res Toxicol 2007;20:625–33. https://doi.org/10.1021/TX600332P.

[39] Wang M, McIntee EJ, Cheng G, Shi Y, Villalta PW, Hecht SS. A Schiff Base Is a Major DNA Adduct of Crotonaldehyde. Chem Res Toxicol 2001;14:423–30. https://doi.org/10.1021/TX000234W.

[40] Ito S, Shen L, Dai Q, Wu SC, Collins LB, Swenberg JA, et al. Tet proteins can convert 5-methylcytosine to 5-formylcytosine and 5-carboxylcytosine. Science (80- ) 2011;333:1300–3. https://doi.org/10.1126/SCIENCE.1210597.

[41] Wang J, Wang Y. Synthesis and characterization of oligodeoxyribonucleotides containing a site-specifically incorporated N6-carboxymethyl-2’-deoxyadenosine or N4-carboxymethyl-2’-deoxycytidine. Nucleic Acids Res 2010;38:6774–84. https://doi.org/10.1093/nar/gkq458.

[42] Lewin MH, Bailey N, Bandaletova T, Bowman R, Cross AJ, Pollock J, et al. Red meat enhances the colonic formation of the DNA adduct O 6-carboxymethyl guanine: Implications for colorectal cancer risk. Cancer Res 2006;66:1859–65. https://doi.org/10.1158/0008-5472.CAN-05-2237.

[43] Wang H, Cao H, Wang Y. Quantification of N2-carboxymethyl-2′-deoxyguanosine in calf thymus DNA and cultured human kidney epithelial cells by capillary high-performance liquid chromatography - Tandem mass spectrometry coupled with stable isotope dilution method. Chem Res Toxicol 2010;23:74–81. https://doi.org/10.1021/tx900286c.

[44] Wang J, Wang Y. Chemical synthesis of oligodeoxyribonucleotides containing N3-and O 4-carboxymethylthymidine and their formation in DNA. Nucleic Acids Res 2009;37:336–45. https://doi.org/10.1093/nar/gkn946.

[45] Cheng G, Reisinger SA, Shields PG, Hatsukami DK, Balbo S, Hecht SS. Quantitation by liquid chromatography-nanoelectrospray ionization-high resolution tandem mass spectrometry of DNA adducts derived from methyl glyoxal and carboxyethylating agents in leukocytes of smokers and non-smokers. Chem Biol Interact 2020;327:109140. https://doi.org/10.1016/J.CBI.2020.109140.

[46] Yuan B, Cao H, Jiang Y, Hong H, Wang Y. Efficient and accurate bypass of N2-(1-carboxyethyl)-2′-deoxyguanosine by DinB DNA polymerase in vitro and in vivo. Proc Natl Acad Sci 2008;105:8679–84. https://doi.org/10.1073/PNAS.0711546105.

[47] Cheng G, Wang M, Villalta PW, Hecht SS. Detection of 7-(2′-Carboxyethyl)guanine but Not 7-Carboxymethylguanine in Human Liver DNA n.d. https://doi.org/10.1021/tx100062v.

[48] Gamboa Da Costa G, Churchwell MI, Hamilton LP, Von Tungeln LS, Beland FA, Marques MM, et al. DNA Adduct Formation from Acrylamide via Conversion To Glycidamide in Adult and Neonatal Mice 2003. https://doi.org/10.1021/tx034108e.

[49] Larisch B, Pischetsrieder M, Severin T. Formation of guanosine adducts from L-ascorbic acid under oxidative conditions. Bioorganic Med Chem Lett 1997;7:2681–6. https://doi.org/10.1016/S0960-894X(97)10056-7.

[50] Goggin M, Seneviratne U, Swenberg JA, Walker VE, Tretyakova N. Column switching HPLC-ESI+-MS/MS methods for quantitative analysis of exocyclic da adducts in the DNA of laboratory animals exposed to 1,3-butadiene. Chem Res Toxicol 2010;23:808–12. https://doi.org/10.1021/tx900439w.

[51] Powley MW, Jayaraj K, Gold A, Ball LM, Swenberg JA. 1,N2-Propanodeoxyguanosine Adducts of the 1,3-Butadiene Metabolite, Hydroxymethylvinyl Ketone. Chem Res Toxicol 2003;16:1448–54. https://doi.org/10.1021/tx030021h.

[52] Paiano V, Maertens L, Guidolin V, Yang J, Balbo S, Hecht SS. Quantitative Liquid Chromatography-Nanoelectrospray Ionization-High-Resolution Tandem Mass Spectrometry Analysis of Acrolein-DNA Adducts and Etheno-DNA Adducts in Oral Cells from Cigarette Smokers and Nonsmokers. Chem Res Toxicol 2020;33:2197–207. https://doi.org/10.1021/ACS.CHEMRESTOX.0C00223.

[53] Bin P, Shen M, Li H, Sun X, Niu Y, Meng T, et al. Increased levels of urinary biomarkers of lipid peroxidation products among workers occupationally exposed to diesel engine exhaust. Free Radic Res 2016;50:820–30. https://doi.org/10.1080/10715762.2016.1178738.

[54] Gonzalez-Reche LM, Koch HM, Weiß T, Müller J, Drexler H, Angerer J. Analysis of ethenoguanine adducts in human urine using high performance liquid chromatography–tandem mass spectrometry. Toxicol Lett 2002;134:71–7. https://doi.org/10.1016/S0378-4274(02)00165-0.

[55] Guo J, Villalta PW, Weight CJ, Bonala R, Johnson F, Rosenquist TA, et al. Targeted and Untargeted Detection of DNA Adducts of Aromatic Amine Carcinogens in Human Bladder by Ultra-Performance Liquid Chromatography-High-Resolution Mass Spectrometry. Chem Res Toxicol 2018;31:1382–97. https://doi.org/10.1021/acs.chemrestox.8b00268.

[56] Guo J, Villalta PW, Turesky RJ. Data-Independent Mass Spectrometry Approach for Screening and Identification of DNA Adducts. Anal Chem 2017;89:11728–36. https://doi.org/10.1021/acs.analchem.7b03208.

[57] Lee SH, Rindgen D, Bible RH, Hajdu E, Blair IA. Characterization of 2‘-Deoxyadenosine Adducts Derived from 4-Oxo-2-nonenal, a Novel Product of Lipid Peroxidation. Chem Res Toxicol 2000;13:565–74. https://doi.org/10.1021/TX000057Z.

[58] Pollack M, Oe T, Lee SH, Elipe MVS, Arison BH, Blair IA. Characterization of 2‘-Deoxycytidine Adducts Derived from 4-Oxo-2-nonenal, a Novel Lipid Peroxidation Product. Chem Res Toxicol 2003;16:893–900. https://doi.org/10.1021/TX030009P.

[59] Knutson CG, Rubinson EH, Akingbade D, Anderson CS, Stec DF, Petrova K V., et al. Oxidation and glycolytic cleavage of etheno and propano DNA base adducts. Biochemistry 2009;48:800–9. https://doi.org/10.1021/BI801654J.

[60] Carvalho VM, Di Mascio P, De Arruda Campos IP, Douki T, Cadet J, Medeiros MHG. Formation of 1,N6-Etheno-2‘-deoxyadenosine Adducts by trans,trans-2,4-Decadienal. Chem Res Toxicol 1998;11:1042–7. https://doi.org/10.1021/TX9800710.

[61] Loureiro APM, Di Mascio P, Gomes OF, Medeiros MHG. trans,trans-2,4-Decadienal-Induced 1,N2-Etheno-2‘-deoxyguanosine Adduct Formation. Chem Res Toxicol 2000;13:601–9. https://doi.org/10.1021/TX000004H.

[62] Schuler D, Eder E. Detection of 1,N2-propanodeoxyguanosine adducts of 2-hexenal in organs of Fischer 344 rats by a 32P-post-labeling technique. Carcinogenesis 1999;20:1345–50. https://doi.org/10.1093/CARCIN/20.7.1345.

[63] Pawłowicz AJ, Munter T, Zhao Y, Kronberg L. Formation of acrolein adducts with 2′-deoxyadenosine in calf thymus DNA. Chem Res Toxicol 2006;19:571–6. https://doi.org/10.1021/tx0503496.

[64] Yang J, Balbo S, Villalta PW, Hecht SS. Analysis of Acrolein-Derived 1, N 2 -Propanodeoxyguanosine Adducts in Human Lung DNA from Smokers and Nonsmokers. Chem Res Toxicol 2019;32:318–25. https://doi.org/10.1021/acs.chemrestox.8b00326.

[65] Pawłowicz AJ, Klika KD, Kronberg L. The structural identification and conformational analysis of the products from the reaction of acrolein with 2′-deoxycytidine, 1-methylcytosine and calf thymus DNA. European J Org Chem 2007:1429–37. https://doi.org/10.1002/EJOC.200600799.

[66] Pawłowicz AJ, Kronberg L. Characterization of adducts formed in reactions of acrolein with thymidine and calf thymus DNA. Chem Biodivers 2008;5:177–88. https://doi.org/10.1002/cbdv.200890009.

[67] Nath RG, Ocando JE, Guttenplan JB, Chung FL. 1,N2-propanodeoxyguanosine adducts: Potential new biomarkers of smoking-induced DNA damage in human oral tissue. Cancer Res 1998;58:581–4.

[68] Eder E, Hoffman C. Identification and Characterization of Deoxyguanosine-Crotonaldehyde Adducts. Formation of 7,8 Cyclic Adducts and 1,N2,7,8 Bis-Cyclic Adducts. Chem Res Toxicol 1992;5:802–8.

[69] Wang M, Mcintee EJ, Cheng G, Shi Y, Villalta PW, Hecht SS. Identification of Paraldol-Deoxyguanosine Adducts in DNA Reacted with Crotonaldehyde 2000. https://doi.org/10.1021/tx000095i.

[70] Stone K, Ksebati MB, Marnett LJ. Investigation of the Adducts Formed by Reaction of Malondialdehyde with Adenosine. Chem Res Toxicol 1990;3:33–8. https://doi.org/10.1021/tx00013a006.

[71] Saieva C, Peluso M, Palli D, Cellai F, Ceroti M, Selvi V, et al. Dietary and lifestyle determinants of malondialdehyde DNA adducts in a representative sample of the Florence City population. Mutagenesis 2016;31:475–80. https://doi.org/10.1093/mutage/gew012.

[72] Ma B, Villalta PW, Balbo S, Stepanov I. Analysis of a malondialdehyde-deoxyguanosine adduct in human leukocyte DNA by liquid chromatography nanoelectrospray-high-resolution tandem mass spectrometry. Chem Res Toxicol 2014;27:1829–36. https://doi.org/10.1021/tx5002699.

[73] Leuratti C, Watson MA, Deag EJ, Welch A, Singh R, Gottschalg E, et al. Detection of Malondialdehyde DNA Adducts in Human Colorectal Mucosa: Relationship with Diet and the Presence of Adenomas 1 n.d.

[74] Stone K, Uzieblo A, Marnett LJ. Studies of the reaction of malondialdehyde with cytosine nucleosides. Chem Res Toxicol 1990;3:467–72. https://doi.org/10.1021/tx00017a013.

[75] Balbo S, Meng L, Bliss RL, Jensen JA, Hatsukami DK, Hecht SS. Time course of DNA adduct formation in peripheral blood granulocytes and lymphocytes after drinking alcohol. Mutagenesis 2012;27:485–90. https://doi.org/10.1093/mutage/ges008.

[76] Matsuda T, Matsumoto A, Uchida M, Kanaly RA, Misaki K, Shibutani S, et al. Increased formation of hepatic N2 -ethylidene-2′-deoxyguanosine DNA adducts in aldehyde dehydrogenase 2-knockout mice treated with ethanol. Carcinogenesis 2007;28:2363–6. https://doi.org/10.1093/carcin/bgm057.

[77] Wang M, McIntee EJ, Cheng G, Shi Y, Villalta PW, Hecht SS. Identification of DNA Adducts of Acetaldehyde. Chem Res Toxicol 2000;13:1149–57. https://doi.org/10.1021/TX000118T.

[78] Pluskota-Karwatka D, Pawłowicz AJ, Kronberg L. Formation of malonaldehyde-acetaldehyde conjugate adducts in calf thymus DNA. Chem Res Toxicol 2006;19:921–6. https://doi.org/10.1021/tx060027h.

[79] Pluskota-Karwatka D, Le Curieux F, Munter T, Sjöholm R, Kronberg L. Identification of Conjugate Adducts Formed in the Reactions of Malonaldehyde-Acetaldehyde and Malonaldehyde-Formaldehyde with Cytidine. Chem Res Toxicol 2002;15:110–7. https://doi.org/10.1021/TX010122K.

[80] Otteneder MB, Knutson CG, Daniels JS, Hashim M, Crews BC, Remmel RP, et al. In vivo oxidative metabolism of a major peroxidation-derived DNA adduct, M1dG. Proc Natl Acad Sci U S A 2006;103:6665–9. https://doi.org/10.1073/PNAS.0602017103.

[81] Knutson CG, Akingbade D, Crews BC, Voehler M, Stec DF, Marnett LJ. Metabolism in vitro and in vivo of the DNA base adduct, M1G. Chem Res Toxicol 2007;20:550–7. https://doi.org/10.1021/TX600334X.

[82] Olsen R, Molander P, Øvrebø S, Ellingsen DG, Thorud S, Thomassen Y, et al. Reaction of Glyoxal with 2′-Deoxyguanosine, 2′-Deoxyadenosine, 2′-Deoxycytidine, Cytidine, Thymidine, and Calf Thymus DNA: Identification of DNA Adducts 2005. https://doi.org/10.1021/tx0496688.

[83] Vaca CE, Fang JL, Conradi M, Hou SM. Development of a 32P-postlabelling method for the analysis of 2’-deoxyguanosine-3’-monophosphate and DNA adducts of methylglyoxal. Carcinogenesis 1994;15:1887–94. https://doi.org/10.1093/CARCIN/15.9.1887.

[84] Byrns MC, Vu CC, Neidigh JW, Abad J-L, Jones RA, Peterson LA. Detection of DNA Adducts Derived from the Reactive Metabolite of Furan, cis-2-Butene-1,4-dial 2006. https://doi.org/10.1021/tx050302k.

[85] Monien BH, Herrmann K, Florian S, Glatt H. Metabolic activation of furfuryl alcohol: formation of 2-methylfuranyl DNA adducts in Salmonella typhimurium strains expressing human sulfotransferase 1A1 and in FVB/N mice. Carcinogenesis 2011;32:1533–9. https://doi.org/10.1093/CARCIN/BGR126.

[86] Monien BH, Engst W, Barknowitz G, Seidel A, Glatt H. Mutagenicity of 5-Hydroxymethylfurfural in V79 cells expressing human SULT1A1: Identification and mass spectrometric quantification of DNA adducts formed. Chem Res Toxicol 2012;25:1484–92. https://doi.org/10.1021/tx300150n.

[87] Yu MW, Lien JP, Liaw YF, Chen CJ. Effects of multiple risk factors for hepatocellular carcinoma on formation of aflatoxin B1-DNA adducts. Cancer Epidemiol Biomarkers Prev 1996;5:613–9.

[88] Woo LL, Egner PA, Belanger CL, Wattanawaraporn R, Trudel LJ, Croy RG, et al. Aflatoxin B1-DNA adduct formation and mutagenicity in livers of neonatal male and female B6C3F1 mice. Toxicol Sci 2011;122:38–44. https://doi.org/10.1093/toxsci/kfr087.

[89] Faucet V, Pfohl-Leszkowicz A, Dai J, Castegnaro M, Manderville RA. Evidence for covalent DNA adduction by ochratoxin A following chronic exposure to rat and subacute exposure to pig. Chem Res Toxicol 2004;17:1289–96. https://doi.org/10.1021/tx049877s.

[90] Essigmann JM, Barker LJ, Fowler KW, Francisco MA, Reinhold VN, Wogan GN. Sterigmatocystin-DNA interactions: identification of a major adduct formed after metabolic activation in vitro. Proc Natl Acad Sci U S A 1979;76:179. https://doi.org/10.1073/PNAS.76.1.179.

[91] Zhao Y, Xia Q, Gamboa Da Costa G, Yu H, Cai L, Fu PP. Full structure assignments of pyrrolizidine alkaloid DNA adducts and mechanism of tumor initiation. Chem Res Toxicol 2012;25:1985–96. https://doi.org/10.1021/tx300292h.

[92] Phillips DH, Miller JA, Miller EC, Adams B. Structures of the DMA Adducts Formed in Mouse Liver after Administration of the Proximate Hepatocarcinogen 1’-Hydroxyestragole1 1981.

[93] Wiseman RW, Fennell TR, Miller JA, Miller EC. Further Characterization of the DNA Adducts Formed by Electrophilic Esters of the Hepatocarcinogens 1′-Hydroxysafrole and 1′-Hydroxyestragole in Vitro and in Mouse Liver in Vivo, Including New Adducts at C-8 and N-7 of Guanine Residues. Cancer Res 1985;45.

[94] Paini A, Punt A, Scholz G, Gremaud E, Spenkelink B, Alink G, et al. In vivo validation of DNA adduct formation by estragole in rats predicted by physiologically based biodynamic modelling. Mutagenesis 2012;27:653–63. https://doi.org/10.1093/MUTAGE/GES031.

[95] Martati E, Boonpawa R, van den Berg JHJ, Paini A, Spenkelink A, Punt A, et al. Malabaricone C-containing mace extract inhibits safrole bioactivation and DNA adduct formation both in vitro and in vivo. Food Chem Toxicol 2014;66:373–84. https://doi.org/10.1016/J.FCT.2014.01.043.

[96] Shen LC, Chiang SY, Lin MH, Chung WS, Wu KY. In vivo formation of N7-guanine DNA adduct by safrole 2′,3′-oxide in mice. Toxicol Lett 2012;213:309–15. https://doi.org/10.1016/J.TOXLET.2012.07.006.

[97] Herrmann K, Engst W, Appel KE, Monien BH, Glatt H. Identification of human and murine sulfotransferases able to activate hydroxylated metabolites of methyleugenol to mutagens in Salmonella typhimurium and detection of associated DNA adducts using UPLC-MS/MS methods. Mutagenesis 2012;27:453–62. https://doi.org/10.1093/MUTAGE/GES004.

[98] Stiborová M, Fernando RC, Schmeiser HH, Frei E, Pafau W, Wiessler M. Characterization of DNA adducts formed by aristolochic acids in the target organ (forestomach) of rats by 32P-postlabelling analysis using different chromatographic procedures. Carcinogenesis 1994;15:1187–92. https://doi.org/10.1093/CARCIN/15.6.1187.

[99] Carlson ES, Upadhyaya P, Villalta PW, Ma B, Hecht SS. Analysis and Identification of 2′-Deoxyadenosine-Derived Adducts in Lung and Liver DNA of F-344 Rats Treated with the Tobacco-Specific Carcinogen 4-(Methylnitrosamino)-1-(3-pyridyl)-1-butanone and Enantiomers of its Metabolite 4-(Methylnitrosamino)-1-(3-pyridyl)-1-butanol. Chem Res Toxicol 2018;31:358–70. https://doi.org/10.1021/acs.chemrestox.8b00056.

[100] Lao Y, Villalta PW, Sturla SJ, Wang M, Hecht SS. Quantitation of Pyridyloxobutyl DNA Adducts of Tobacco-Specific Nitrosamines in Rat Tissue DNA by High-Performance Liquid Chromatography-Electrospray Ionization-Tandem Mass Spectrometry 2006. https://doi.org/10.1021/tx050351x.

[101] Stepanov I, Hecht SS. Mitochondrial DNA adducts in the lung and liver of F344 rats chronically treated with 4-(methylnitrosamino)-1-(3-pyridyl)-1-butanone and (S)-4-(methylnitrosamino)-1-(3-pyridyl)-1-butanol. Chem Res Toxicol 2009;22:406. https://doi.org/10.1021/TX800398X.

[102] Wang M, Cheng G, Sturla SJ, Shi Y, McIntee EJ, Villalta PW, et al. Identification of Adducts Formed by Pyridyloxobutylation of Deoxyguanosine and DNA by 4-(Acetoxymethylnitrosamino)-1-(3-pyridyl)-1-butanone, a Chemically Activated Form of Tobacco Specific Carcinogens. Chem Res Toxicol 2003;16:616–26. https://doi.org/10.1021/TX034003B.

[103] Michel AK, Zarth AT, Upadhyaya P, Hecht SS. Identification of 4-(3-Pyridyl)-4-oxobutyl-2′-deoxycytidine Adducts Formed in the Reaction of DNA with 4-(Acetoxymethylnitrosamino)-1-(3-pyridyl)-1-butanone: A Chemically Activated Form of Tobacco-Specific Carcinogens. ACS Omega 2017;2:1180–90. https://doi.org/10.1021/acsomega.7b00072.

[104] Leng J, Wang Y. Liquid Chromatography-Tandem Mass Spectrometry for the Quantification of Tobacco-Specific Nitrosamine-Induced DNA Adducts in Mammalian Cells. Anal Chem 2017;89:9124–30. https://doi.org/10.1021/acs.analchem.7b01857.

[105] Totsuka Y, Takamura-Enya T, Kawahara N, Nishigaki R, Sugimura T, Wakabayashi K. Structure of DNA Adduct Formed with Aminophenylnorharman, Being Responsible for the Comutagenic Action of Norharman with Aniline 2002. https://doi.org/10.1021/tx020007p.

[106] Jamin EL, Arquier D, Canlet C, Rathahao E, Tulliez J, Debrauwer L. New insights in the formation of deoxynucleoside adducts with the heterocyclic aromatic amines PhIP and IQ by means of ion trap MSn and accurate mass measurement of fragment ions. J Am Soc Mass Spectrom 2007;18:2107–18. https://doi.org/10.1016/J.JASMS.2007.09.008.

[107] Turesky RJ, Gremaud E, Markovic J, Snyderwine EG. DNA Adduct Formation of the Food-Derived Mutagen 2-Amino-3-methylimidazo[4,5-f]quinoline in Nonhuman Primates Undergoing Carcinogen Bioassay. Chem Res Toxicol 1996;9:403–8. https://doi.org/10.1021/TX950132J.

[108] Turesky RJ, Markovic J, Aeschlimann JM. Formation and Differential Removal of C-8 and N2-Guanine Adducts of the Food Carcinogen 2-Amino-3-methylimidazo[4,5-f]quinoline in the Liver, Kidney, and Colorectum of the Rat. Chem Res Toxicol 1996;9:397–402. https://doi.org/10.1021/TX950131R.

[109] Kim S, Guo J, O’Sullivan MG, Gallaher DD, Turesky RJ. Comparative DNA adduct formation and induction of colonic aberrant crypt foci in mice exposed to 2-amino-9H-pyrido[2,3-b]indole, 2-amino-3,4-dimethylimidazo[4,5-f]quinoline, and azoxymethane. Environ Mol Mutagen 2016;57:125–36. https://doi.org/10.1002/EM.21993.

[110] Totsuka Y, Fukutome K, Takahashi M, Takahashi S, Tada A, Sugimura T, et al. Presence of N2 -(deoxyguanosin-8-yl)-2-amino-3,8-dimethylimidazo[4,5- f ]quinoxaline (dG-C8-MeIQx) in human tissues. Carcinogenesis 1996;17:1029–34. https://doi.org/10.1093/CARCIN/17.5.1029.

[111] Bessette EE, Goodenough AK, Langouët S, Yasa I, Kozekov ID, Spivack SD, et al. Screening for DNA adducts by data-dependent constant neutral loss-triple stage mass spectrometry with a linear quadrupole ion trap mass spectrometer. Anal Chem 2009;81:809–19. https://doi.org/10.1021/AC802096P/SUPPL_FILE/AC802096P_SI_002.PDF.

[112] Frandsen H, Grivas S, Turesky RJ, Andersson R, Dragsted LO, Larsen JC. Formation of DNA adducts by the food mutagen 2-amino-3,4,8-trimethyl-3H-imidazo[4,5-f]quinoxaline (4,8-DiMeIQx) in vitro and in vivo. Identification of a N2-(2’-deoxyguanosin-8-yl)-4,8-DiMeIQx adduct. Carcinogenesis 1994;15:2553–8. https://doi.org/10.1093/CARCIN/15.11.2553.

[113] Hashimoto Y, Shudo K, Okamoto T. Modification of nucleic acids with muta-carcinogenic heteroaromatic amines in vivo: Identification of modified bases in DNA extracted from rats injected with 3-amino-1-methyl-5H-pyrido-[4,3-b]indole and 2-amino-6-methyldipyrido-[1,2-a:3′,2′-d]imidazole. Mutat Res Lett 1982;105:9–13. https://doi.org/10.1016/0165-7992(82)90200-7.

[114] Frederiksen H, Frandsen H, Pfau W. Syntheses of DNA adducts of two heterocyclic amines, 2-amino-3-methyl-9 H -pyrido[2,3- b ]indole (MeAαC) and 2-amino-9 H -pyrido[2,3- b ]indole (AαC) and identification of DNA adducts in organs from rats dosed with MeAαC. Carcinogenesis 2004;25:1525–33. https://doi.org/10.1093/CARCIN/BGH156.

[115] Xiao S, Guo J, Yun BH, Villalta PW, Krishna S, Tejpaul R, et al. Biomonitoring DNA adducts of cooked meat carcinogens in human prostate by nano liquid chromatography-high resolution tandem mass spectrometry: Identification of 2-amino-1-methyl-6-phenylimidazo[4,5-b]pyridine DNA adduct. Anal Chem 2016;88:12508–15. https://doi.org/10.1021/acs.analchem.6b04157.

[116] Jones CR, Sabbioni G. Identification of DNA adducts using HPLC/MS/MS following in vitro and in vivo experiments with arylamines and nitroarenes. Chem Res Toxicol 2003;16:1251–63. https://doi.org/10.1021/TX020064I.

[117] Cui L, Sun HL, Wishnok JS, Tannenbaum SR, Skipper PL. Identification of adducts formed by reaction of N-acetoxy-3,5- dimethylaniline with DNA. Chem Res Toxicol 2007;20:1730–6. https://doi.org/10.1021/tx700306c.

[118] Yamazoe Y, Roth RW, Kadlubar FF. Reactivity of benzidine diimine with DNA to form N-(deoxyguanosin-8-yl)-benzidine. Carcinogenesis 1986;7:179–82. https://doi.org/10.1093/CARCIN/7.1.179.

[119] Covalent Binding of Benzidine and N-Acetylbenzidine to DNA at the C-8 Atom of Deoxyguanosine in Vivo and in Vitro. Cancer Research 1982; 42:2678-2696

[120] Westra JG, Flammang TJ, Fullerton NF, Beland FA, Weis CC, Kadlubar FF. Formation of DNA adducts in vivo in rat liver and intestinal epithelium after administration of the carcinogen 3,2’-dimethyl-4-aminobiphenyl and its hydroxamic acid. Carcinogenesis 1985;6:37–44. https://doi.org/10.1093/CARCIN/6.1.37.

[121] Tullis DL, Dooley KL, Miller DW, Baetcke KP, Kadlubar FF. Characterization and properties of the DNA adducts formed from N-methyl-4-aminoazobenzene in rats during a carcinogenic treatment regimen. Carcinogenesis 1987;8:577–83. https://doi.org/10.1093/CARCIN/8.4.577.

[122] Kadlubar FF, Miller JA, Miller EC. Guanyl O6-Arylamination and O6-Arylation of DMA by the Carcinogen N-Hydroxy-1-naphthy lam Â¡ne1 n.d.

[123] Kadlubar FF, Unruh LE, Beland FA, Straub KM, Evans FE. In vitro reaction of the carcinogen, N-hydroxy-2-naphthylamine, with DNA at the C-8 and N2 atoms of guanine and at the N6 atom of adenine. Carcinogenesis 1980;1:139–50. https://doi.org/10.1093/CARCIN/1.2.139.

[124] Levy GN, Weber WW. 2-Aminofluorene-DNA adduct formation in acetylator congenic mouse lines. Carcinogenesis 1989;10:705–9. https://doi.org/10.1093/CARCIN/10.4.705.

[125] Yamazoe Y, Miller DW, Weis CC, Dooley KL, Zenser T V., Beland FA, et al. DNA adducts formed by ring-oxidation of the carcinogen 2-naphthylamine with prostaglandin H synthase in vitro and in the dog urothelium in vivo. Carcinogenesis 1985;6:1379–87. https://doi.org/10.1093/CARCIN/6.9.1379.

[126] Morden BH, Müller C, Engst W, Frank H, Seidel A, Glatt H. Time course of hepatic 1-methylpyrene DNA adducts in rats determined by isotope dilution LC-MS/MS and 32P-postlabeling. Chem Res Toxicol 2008;21:2017–25. https://doi.org/10.1021/TX800217D.

[127] Devanesan PD, RamaKrishna NVS, Todorovic R, Rogan EG, Cavalieri EL, Jeong H, et al. Identification and quantitation of benzo[a]pyrene-DNA adducts formed by rat liver microsomes in vitro. Chem Res Toxicol 2002;5:302–9. https://doi.org/10.1021/TX00026A024.

[128] Singh R, Teichert F, Seidel A, Roach J, Cordell R, Cheng MK, et al. Development of a targeted adductomic method for the determination of polycyclic aromatic hydrocarbon DNA adducts using online column-switching liquid chromatography/tandem mass spectrometry. Rapid Commun Mass Spectrom 2010;24:2329–40. https://doi.org/10.1002/RCM.4645.

[129] Villalta PW, Hochalter JB, Hecht SS. Ultrasensitive High-Resolution Mass Spectrometric Analysis of a DNA Adduct of the Carcinogen Benzo[a]pyrene in Human Lung. Anal Chem 2017;89:12735–42. https://doi.org/10.1021/acs.analchem.7b02856.

[130] Reardon DB, Prakash AS, Hilton BD, Roman JM, Pataki J, Harvey RG, et al. Characterization of 5-methylchrysene-1,2-dihydrodiol-3,4-epoxide-DNA adducts. Carcinogenesis 1987;8:1317–22. https://doi.org/10.1093/CARCIN/8.9.1317.

[131] Szeliga J, Hilton BD, Chmurny GN, Krzeminski J, Amin S, Dipple A. Characterization of DNA Adducts Formed by the Four Configurationally Isomeric 5,6-Dimethylchrysene 1,2-Dihydrodiol 3,4-Epoxides. Chem Res Toxicol 1997;10:378–85. https://doi.org/10.1021/TX960178S.

[132] Stack DE, Byun J, Gross ML, Rogan EG, Cavalieri EL. Molecular characteristics of catechol estrogen quinones in reactions with deoxyribonucleosides. Chem Res Toxicol 1996;9:851–9. https://doi.org/10.1021/tx960002q.
